# Supplementary material for: Protocol for a multinational risk-stratified randomised controlled trial in paediatric Crohn’s disease: methotrexate versus azathioprine or adalimumab for maintaining remission in patients at low or high risk for aggressive disease course
Source: BMJ Open. 2020 Jul 1;10(7):e034892. doi: 10.1136/bmjopen-2019-034892 (PMC7332179; doi:10.1136/bmjopen-2019-034892)
Supplement: Supplementary data [file bmjopen-2019-034892supp002.pdf]

Participating Sites – REDUCE-RISK in CD Study			
**Country**	**City**	**Site**	**Ethics committee**
**Belgium**	Brussels	Universitair Ziekenhuis	Commissie Ethiek, UZ **Brussel**
	Brussels	Clinique Saint Luc UCL	
	Liège	Clinique de l'Espérance	
	Brussels	HUDERF	
**Canada**	Toronto	SickKids	SickKids Research Ethics Board, **Toronto**
**Czech Republic**	Prague	FN Motol	Eticka Komise, **Plzen**<br>Eticka Komise, VFN **Praha**<br>Eticka Komise, FN Motol **Praha**
	Plzeň	FN Plzeň	
	Prague	First Medical Faculty	
**France**	Paris	Hôpital Necker Enfants Malades	CPP Hôpital Necker, **Paris**
	Paris	Hôpital Robert Debré	
	Paris	Hôpital Armand Trousseau	
	Le Havre	Hôpital Jacques Monod	
	Nancy	Hôpitaux de Brabois	
	Toulouse	Hôpital des Enfants	
	Tours	Hôpital Clocheville	
	Caen	CHU Caen Côte de Nacre	
	Marseille	Hôpital de la Timone	
**Germany**	Munich	Childrens Hospital	Ethikkommission LMU, **München**
	Ulm	Universitätsklinikum	
	Hannover	MHH Kinderklinik	
	Giessen	UKGM	
	Berlin	Charite Hospital	
**Greece**	Athens	Children's Hospital "AGIA SOFIA"	Ethics Committee, **Athens**
**Israel**	Jerusalem	Shaare Zedek Medical Center	Helsinki Committee, Schneider Medical Center, **Petah Tikva**<br>Ethics and Research Committe, Wolfson Medical Center, **Tel Aviv**<br>Institutional Review Board, SZMC, **Jerusalem**
	Tel Aviv	Wolfson Medical Center	
	Petah Tikva	Schneider Children's Medical Center	
	Ramat Gan	Sheba Medical Center	
	Haifa	Rambam Medical Center	
**Italy**	Rome	Università degli Studi di Roma La Sapienza	Comitato Etico dell'Universita' "SAPIENZA", **Roma**<br>Comitato Etico Regionale per la Sperimentazione Clinica della Regione Toscana Sezione, **Firenze**<br>Comitato Etico, Servizio Saintario Regionale, **Bologna**
	Bologna	Maggiore Hospital	
	Florence	Azienda Ospedaliero Universitaria	
	Parma	Azienda Ospedaliero	
	Rome	Opsedale Pediatrico Bambino Gesù	
**Netherlands**	Rotterdam	Erasmus Medical Center	Medische Etische Toetsings Commissie, Erasmus MC,

			**Rotterdam**
**Poland**	Warsaw	Centrum Zdrowia MDM	Bioethical Commission at the Institute of Polish Mother's Health Center, **Lodz**
	Białystok	Uniwersytecki Dziecięcy Szpital Kliniczny	
	Łódź	Instytut Centrum Zdrowia Matki Polki	
**United Kingdom**	Glasgow	The Royal Hospital for Children	North West - Liverpool East Research Ethics Committee, NHS, **Manchester**
	London	Royal London Children’s Hospital, Barts Health NHS Trust	
	Edinburgh	Sick Children’s Hospital	
	Birmingham	Children's Hospital	
	Oxford	John Radcliffe Hospital	

Supplementary Table 1 – Sites participating in REDUCE-RISK in CD
