# Supplementary material for: Protocol for a multinational risk-stratified randomised controlled trial in paediatric Crohn’s disease: methotrexate versus azathioprine or adalimumab for maintaining remission in patients at low or high risk for aggressive disease course
Source: BMJ Open. 2020 Jul 1;10(7):e034892. doi: 10.1136/bmjopen-2019-034892 (PMC7332179; doi:10.1136/bmjopen-2019-034892)
Supplement: Supplementary data [file bmjopen-2019-034892supp001.pdf]

INFORMED CONSENT FORM

Parents/Guardian Informed Consent Form for Participation of a Minor in a Clinical Trial

Risk-stratified randomized controlled trial in paediatric Crohn's Disease: Methotrexate versus azathioprine or adalimumab for maintaining remission in patients at low or at high risk for aggressive disease course, respectively – a treatment strategy.

Dear parents,

Your child's doctor, Dr…………………………, working at ………………………………….. Hospital, propose your child to participate in a clinical trial related to its disease.

It is important to read this note carefully before taking any decision. Do not hesitate to ask the physician all the questions you may have about it.

The participation of your child is based on volunteering. Therefore, your child can refuse to participate or stop its participation in the trial at any time, all of this without prejudice to the patient's right to receive the standard treatment.

If you refuse your child to participate, he/she will still receive the best medical support.

Purpose of the research and trial's objectives

Your child has just been diagnosed with Crohn's disease. The disease is caracterised by chronic inflammation of the digestive track (bowel/colon). This disease changes over between remission period and relapse period. There are efficient drugs able to prevent relapse and to maintain remission. In order to reduce the likelihood of long-term complications, induction treatment has already been prescribed to your child. This first treatment has to be followed up by a maintenance treatment that will be introduced to avoid the inflammation from returning. Consensus guidelines of ECCO / ESPGHAN (french and european IBD specialized organizations) recommend 3 efficient treatments: either immunosuppressive treatment with Thiopurines (azathioprine and 6-mercaptopurine), or Methotrexate or anti TNF (adalimumab).

So far, no clinical trial has been conducted to compare those 3 treatments in children with Crohn's disease and to answer the following question: "Which treatment is the most efficient, for which patient and/or in which situation?"

Progression of Crohn's disease is not the same for all patients. That's why this study will first classified all children in high and low risk groups based on more or less severe course of Crohn's disease. The lower risk group will be randomized (which is like tossing a coin) to receive either thiopurines or methotrexate as maintenance treatment. The high risk group will be randomized to receive either methotrexate or adalimumab. Results will show whether there is different efficiency between the 3 drugs for patients with a more or less severe disease.

Sponsor

PIBD-Net (www.pibd-net.org) is a global, international and non-profit organisation gathering physicians and researchers specialised in inflammatory bowel diseases. The acronym stands for Pediatric Inflammatory Bowel Diseases Network and it is present in 31 countries (Europe, North America, Australia and Japan). This organisation is dedicated in improving the medical care of children with inflammatory bowel disease through the establishment of clinical researches.

PIBD-Net and partners received funding from European Commission for Horizon 2020 program (project no.668023) in order to perform this research.

The approximate number of participants and duration of follow-up

A total of 312 new-onset children with Crohn's disease (136 in that high-risk group and 176 in the low risk) will be enrolled in many sites around the world. The period of recruitment is 45 months and your child will be followed up for 12 months after enrolment.

What will happen to your child during the trial?
If you agree to have your child participating in this study, your child will be first directed into one of two groups based on certain predictors of its disease (such as its location and severity). You will know which group your child is in. Next, your child will be randomized to receive maintenance treatment namely:

- **METHOTREXATE or AZATHIOPRINE for the low risk group** ;
- **METHOTREXATE orADALIMUMABfor the high risk group** ;

You cannot choose the treatment group but you will know which drug your child will receive.

<u>You will not be asked to come to clinic just because of this study which is designed to mirror regular follow-up in clinic.</u> After signing the informed consent allowing your child to be part of this research, your child will have clinic visit every 2 months during the first 6 months and then every 3 months during the last 6 months. At each visit, a clinic examination is performed and blood, urine and stool samples are collected (this process follows our standard clinical practices of our patients not involved in this protocole).

Your doctor, your child and yourself will be asked to complete short questionnaires to evaluate the quality of life of your child. <u>Most of the recorded data for this study is needed anyway as part of a regular visit</u> but there might be a few more questions we will ask you for this study.

We will also contact you over the phone at week 4 to ask how your child feels regarding its Crohn's disease, whether your child has any bad reactions to the medications your child will receive and check your child compliance to the treatment.

To optimize the medications we prescribe, we will draw 12 ml (3-4 teaspoons) of blood at inclusion visit, 10ml at visit V2, and then 5ml at each next study visits to measure the level of the medications your child will receive. A urine sample will be collected at inclusion visit and a DNA sample (either 5ml of blood or buccal swab) will be collected at the beginning of the study, and also in case of drug intolerance.

We will also collect your child stool (poop) six times during the year to measure the amount of inflammation in its bowel as well as bacteria flora in its intestine.

We will evaluate whether the drugs work based on

- completed questionnaires
- clinic examination
- results of biological samples

Optional Ancillary study (« ADA STEP-up »)
In case of failing (intolerance or relapse) of your child immunomodulator therapy (: either azathioprine/6MP or methotrexate), your child will be invitated to participate in the ancillary study. If you agree, your child will be prescribed adalimumab during 12 months.
This adalimumab treatment can increase the study duration by a maximum of 9 months, meaning a maximum of 3 additional visits. Those visits are identical to the regular follow up study visits.

The expected benefits to the participant or to others because of the trial

The medications your child will receive in this study are not experimental and thus there are no direct benefit for using these drugs that are available outside of the study. However, your child will be monitored closely to ensure optimization of the treatment by adapting drug amounts based on new analyses (urine, DNA, blood and stool samples). In addition, patients involved in this study have access to molecular analyses in order to better understand why a patient is less responsive than expected. That might result in a more tight control of the disease and better monitoring of the treatment of your child.

Harris RE, *et al. BMJ Open* 2020; 10:e034892. doi: 10.1136/bmjopen-2019-034892

After study completion, we will have the required data to recommend how to use these medications in new children who develop Crohn's disease.

Risks added by the research

As previously mentionned, all medications used in this trial are not experimental and are being used very often in clinical practice in children/adolescents and adults with Crohn's disease. There is no additional risk compares to regular clinical practices. The known risks and discomfort that may be anticipated are listed below.

Known risks and discomfort that may be anticipated

The medications yourchild will receive in this study are not experimental but used in regular practices. They can also be associated with side effects (all described in the corresponding drug information sheet).

- ✓ **METHOTREXATE: weekly subcutaneous (under the skin)** This drug may be associated with side effects mainly in the day of the injection including flu-like symptoms, nausea, vomiting, headache or fatigue. Your child will be asked to take a vitamin called folic acid which will reduce these non-dangerous side effects. This injection may cause slight discomfort.METHOTREXATE may cause reduced blood counts, especially white blood cells and elevated liver enzymes. Thoses parameters will be checked regularly. In this study, molecular analyses will be performed to screen patients who can't tolerate METHOTREXATE..This drug causes an unusual sensitiveness to the sun (however, there is no data stating that it increases the risk of cancer or lymphoma). METHOTREXATE can cause foetal abdormalities so pregnancy is not allowed and efficient contraceptive is essential (for both male and female).
- ✓ **AZATHIOPRINE (or 6MP): to be taken orally.** THIOPURINES may cause reduced blood counts, especially white blood cells and elevated liver enzymes. Thoses parameters will be checked regurlarlyIn this study, molecular analyses will be done in order to screen patients who can't tolerate those drugs. In some rare cases (<3%), the drug may cause inflammation in the pancreas which is usually mild and not dangerous. As all drugs, THIOPURINES can't be tolerated by some patients due to allergy. Approximately 10% of children will not tolerate the drug because of nausea, vomiting, tummy pain, diarrhea, headaches or fever..THIOPURINES are associated with an increased infectious risk (about 1%). Infections are likely cause by viruses. In rare cases, THIOPURINES may increase risk for blood cancer called lymphoma (especially for patients > 65 year old).–This drug causes an unusual sensitiveness to the sun and can be associated with skin cancer in case of significant sun exposure.

ADALIMUMAB: subcutaneous (under the skin) injections every 2 weeks Adalimumab is associated with minor pain during the injection and local reactions could appear with minimal significance..ADALIMUMAB is associated with an increased infectious risk. However, serious infections are uncommon. Before starting ADALIMUMAB treatment, tuberculosis must be excluded. With time, the effect of adalimumab may wain as a result of the development of antibodies against the drug. Skin inflammatory damages (such as « psoriasis ») were observed in some patients. Anti TNF drugs have been closely monitored since their use as standard treatment. Those drugs may be responsible of heart failure for patients with severe heart disease, hepatitis, decreasing blood cells, demyelinating neurologic disease, or lupus (without affecting main organs). In addition, some cases of cancer have been notified in patient treated by ADALIMUMAB, but risks of cancer is slightly increased only for melanoma. Number of cancer seems not to be increased compared to patients with Crohn's diseases and without being treated with those drugs.

Circumstances under which participation in the medical trial may be discontinued in accordance with the decision of the investigator or the Sponsor:

a. The doctor has the right to take your child out of the study at any time. This will be made after clinical considerations, your child's side effects from the drugs, intolerance to the drugs or lose of response.

b. Regulatory authorities (Ministery of Health or Ethics committee), may stop your child participating in the study.

Harris RE, *et al. BMJ Open* 2020; 10:e034892. doi: 10.1136/bmjopen-2019-034892

An explanation of alternative treatments, their advantages and disadvantages, if any, for the participant:

The current standard therapy for maintenance therapy in Crohn's disease is either METHOTREXATE or thiopurines or anti-TNF biologics for the more severe Crohn diseaes. This is exactly the medications given also as part of this study. The difference is that instead letting you and the doctor choose between the options, the choice is standardized based on predictive variables of your disease and randomization. If you choose not to have your child participating in the study, your child will likely receive anyway one or more of these three drugs. The only exception would be that if anti-TNF is prescribed, either adalimumab or infliximab can be given and as part of this study your child will receive adalimumab only. However, your child will not have access to molecular analyses described in this protocole with a close monitoring of drug safety. Indeed, those 'new" analyses are not done in the standardclinical practise of Crohn's disease.

If you participate in this study, what will you have to do more than usual ?

If you agree to have your child participating in this study, please make sure to follow the listed points belowPlease come at your appointements with your child. If not possible, please inform its physician as soon as possible

- Please ensure that your child takes the treatment as instructed by its doctor
- Please inform the physician involved in the study of any event happening during the research (such as hospitalization,…)
- Your child must not participate in any other clinical trial that involves the use of an investigational product throughout the course of this trial. It is to avoid accidents such as possible interactions between medicines.

Biological samples collected during this research project

If you agree to have your child participating in this research, additional blood, urine and stool samples will be collected at the same time as our standard clinic samplings. Please see below:

- ✓ 10ml of blood during inclusion visit (on randomisation day).
- ✓ 5ml of blood (PAX tube) during inclusion visit for RNA analyses
- ✓ 10ml of blood at follow up visits M2 (2 months after inclusion)
- ✓ 5 ml of blood at follow up visits M4, M6, M9 and M12 (4, 6, 9, 12 months after inclusion)
- ✓ Stool sample at inclusion visit and follow up visits M2, M4, M6, M9 and M12 (2, 4, 6, 9, 12 months after inclusion visit).
- ✓ A DNA sample will be collected at inclusion visit and in case of intolerance of one of the drugs for DNA analyses.
- ✓
- ✓ Urine sample (15ml) will be collected at M2 visit (2 months after inclusion).

Those samples will be sent to specialized laboratories in order to be used to perform specific studies such as adalimumab, methotrexate, thiopurine analyses and serology, genetic (both DNA and RA), microbiology studies. They will also be re-used for further testing on Crohn's disease, its diagnosis and its treatment as well as efficacy and tolerance by molecular ("omic") analyses.

At any time, you can request to your clinician to have those biological samples destroyed or not to be used for further researches.

Confidentiality

As part of biomedical research in which PIBD-Net sponsor proposes your child's participation, treatment of personal data will be set up to analyse results of this research based on its aim. Therefore, your child medical data and quality of life will be transferred to PIBD-Net sponsor. Those data will be anonymous and identified by a coded number and its initials. Those confidential data could be transferred to local and foreign authorities. If your child has to be withdrawn for any reasons, collected data prior its withdrawal will be used unless you do not want

Harris RE, *et al. BMJ Open* 2020; 10:e034892. doi: 10.1136/bmjopen-2019-034892

them to. Then, you will have to inform the physician accordingly.

According to the EU General Data Protection Regulation (GDPR) dated on 26May2018, you have the right to access to your child and your personal data, modify them and oppose the use of your child and yourdata. You have also the right to request that your child and your personal data are erased, are limited in use, and to ask for a complete copy of all data collected from you and your child for the study. You can contact the Data Privacy Officer (DPO) of the sponsor at any time at dpo@pibd-net.org for any request regarding your child and your personal data.

Data collected for the study are transferred outside of the EU, as our database is based in Israel. However, we guarantee that data protection will be as strict as requested by GDPR.

Voluntary participation

Your participation in this research is entirely voluntary. It is your choice whether to have your child participating or not, all the services your child receives at this hospital will continue and nothing will change. If you choose not to participate in this research project, your child will be offered the treatment that is routinely offered in this hospital for Crohn’s disease. You may change your mind later and stop participating even if you agreed earlier.

Right to refuse or withdraw

Your child does not have to take part in this research if you do not wish to do so and refusing to participate will not affect its treatment in any way. Your child will still have all the benefits that it would otherwise have at this hospital. You may stop participating in the research at any time that you wish without losing any of its rights as a patient here. Its treatment at this hospital will not be affected in any way.

Alternatives to participating

If you do not wish that your child takes part in the research, your child will be provided with the established standard treatment available at this hospital.

Reimbursement

There is no reimbursement for participating in this study. There are no special visits to the hospital excepted during this study. All DNA, blood, urine and stool samples will be taken at the time of a routine clinic visit.

<u>This proposal has been reviewed and approved by [name of the local IRB], which is a committee whose task it is to make sure that research participants are protected from harm.</u>

Harris RE, *et al. BMJ Open* 2020; 10:e034892. doi: 10.1136/bmjopen-2019-034892

Informed consent form

We, the undersigned :

M, Miss, *(name, first name of parent/legal guardian)* …………………………………………………………………..

M, Miss, *(name, first name of parent/legal guardian)* …………………………………………………………………..

I, M, Miss, ……………………………………………………………………….*(name, first name of parent)* hereby declare that my consent below has been given voluntarily and that I have understood all of the above. I undertake to also inform the child's father/mother *(please cross off as appropriate)* of my consent for the participation of our child in the clinical trial. If the child's father/mother *(please cross off as appropriate)* does not agree to affix his/her consent to mine, I undertake to inform the physician in-charge and to withdraw my consent for the participation of my child in the clinical trial. I have also received a lawfully and dated copy of this informed consent form

I agree that my child (name, first name of the child)………………………………………………………**takes part of the study named "***Risk-stratified randomized controlled trial in paediatric Crohn's Disease : Methotrexate versus azathioprine or adalimumab for maintaining remission in patients at low or at high risk for aggressive disease course, respectively – a treatment strategy* " , managed by PIBD Net. It has been explained to us by *(name, first name of explaining investigator /sub investigator, phone,)*…………………………………………… …… ………………………………………………………………………………………physician in this clinical trial.

- We hereby declare that we agree for our child to participate in the clinical trial as detailed in this document
- Our child has been informed and agreed to take part of this clinical trial.
- We had the opportunity to ask all the questions we had to the physician who explained potential risks and constraints linked to our child participation in this clinical trial.
- We received appropriate answers to all our questions
- We hereby declare that at the time of signing this document, our child is not participating in another clinical trial that involves the use of any investigational product, and that we undertake that our child will not participate in any other clinical that involves the use of an investigational product throughout the course of this trial.
- We declare that our child has a health insurance.
- .We hereby declare that we are free to choose that our child will not participate in the clinical trial, and that we are free to stop our child participation in the trial at any time, and all of this without prejudice to our child's right to receive the standard treatment. Then, we will inform the physician whether data collected prior our decision can be used or not.
- We have been informed that the doctor has the right to take our child out of the study at any time, if needed.
- That in case of completing a questionnaire – we are entitled not to answer all or some of the questions in the questionnaire.
- We are informed that samples collected during this clinical trial will be kept and used for further testing on Crohn's disease. We can decide at any time not to have those samples used by informing our child physician.
- That we are guaranteed confidentiality concerning the identity of the patient and that of the parents/guardians. This confidentiality will be kept by all those concerned with and involved in the clinical trial, and their identity will not be disclosed in any publication.
- That the Medical Institution has arranged for appropriate insurance coverage of the investigators, physicians and medical staff involved in the clinical trial, against claims filed by clinical trial participants and/or third party claims related to the clinical trial, either during the course of the trial or thereafter. This is

Harris RE, *et al. BMJ Open* 2020; 10:e034892. doi: 10.1136/bmjopen-2019-034892

PIBD-SETQuality

European Commission

without prejudice to our rights under the law.

- That in case of pregnancy during the course of the clinical trial, the girl/woman will be counselled (by the principal investigator) concerning the possible effects on the foetus and the fate of the pregnancy, including the possibility of discontinuing the pregnancy.
- We hereby declare that our below consent has been given voluntarily and that we have understood all of the above mentioned. We also received a lawfully signed and dated copy of this informed consent.
- By signing this consent form, we authorize the sponsor of the clinical trial, the Institutional Helsinki Committee, the auditing entity at the Medical Institute and the Ministry of Health direct access to the patient's medical file, to verify the clinical trial methods and the clinical data. This access to our child medical information will be performed with confidentiality maintained, according to the laws and procedures of maintaining confidentiality.
- We declare that we are informed and give our approval to receive all information related to our child participation in this clinical trial. We know that data will only be used for treatment and follow up cares
- We hereby declare that we know and agree to have the information on our child's participation in the clinical trial provided to his/her attending physician at the HMO/Health care Services with which our child is insured, in case the clinical trial involved the provision of services : performing medical examinations or supplying devices or products or implants. We know that the HMO will not use this information for purposes other than medical treatment and follow up

I agree to have my child participating in the ancillary study (« ADA STEP-up »)

Yes ☐ No ☐ *[please tick]*

**Signature of parents or guardians/representatives of the patient**	**Signature of the child**
Name, First Name :______________________________<br>Date : Signature :<br><br>Name, First Name :______________________________<br>Date : Signature :	Name, First Name :________________________________<br>Date : Signature :

Declaration of the Investigator/Sub-Investigator :This consent was obtained by me after I have explained all the above mentioned to the parents (or guardians) of the clinical trial participant and ensure that all my explanations were understood by them.

**Investigator/Sub-investigator' Signature :**
Name, First Name: ____
Date: Signature :

Harris RE, *et al. BMJ Open* 2020; 10:e034892. doi: 10.1136/bmjopen-2019-034892

PIBD-SETQuality

European Commission

This is a triplicate document. First / original copy to be kept by the investigator for 15 years, second copy to be given to parents or legal guardians, third copy to be kept in Investigator files (under sealed envelope).

Harris RE, *et al. BMJ Open* 2020; 10:e034892. doi: 10.1136/bmjopen-2019-034892

PIBD-SETQuality

European Commission

Informed consent for Genetic Analyses

Hereby declare that we agree for genetic examinations of our child to study genes involved in tolerance / non tolerance of the drugs by molecular (“omic”) analyses and analyses of drug efficacy in Crohn disease’s patients.

Hereby declare that we agree that all recorded data collected during this trial including genetic data can be processed by the sponsor or acting as sponsor. I understand that, as stipulated in the General Data Protection Regulation, I can access, modify, erase or ask for a copy of my child’s personal data and my personal data at any time, by asking to the investigator who will contact the sponsor.

We can decide not to participate anymore in the genetic part of the trial by informing our doctor who will inform the sponsor.

Yes ☐ No ☐ *[please tick]*

Hereby declare that we agree that all biological samples collected during this trial can be used for future genetic research on Crohn’s disease.

Yes ☐ No ☐ *[please tick]*

Parents/guardians Signature: Investigator Signature:

Name, First name: Name, First name:

Date : Signature : Date : Signature :

Name, First name:

Date : Signature :

Harris RE, *et al. BMJ Open* 2020; 10:e034892. doi: 10.1136/bmjopen-2019-034892
