# Supplementary material for: Protocol for a multinational risk-stratified randomised controlled trial in paediatric Crohn’s disease: methotrexate versus azathioprine or adalimumab for maintaining remission in patients at low or high risk for aggressive disease course
Source: BMJ Open. 2020 Jul 1;10(7):e034892. doi: 10.1136/bmjopen-2019-034892 (PMC7332179; doi:10.1136/bmjopen-2019-034892)
Supplement: Author's manuscript [file bmjopen-2019-034892.draft_revisions.pdf]

BMJ Open

BMJ Open is committed to open peer review. As part of this commitment we make the peer review history of every article we publish publicly available.

When an article is published we post the peer reviewers' comments and the authors' responses online. We also post the versions of the paper that were used during peer review. These are the versions that the peer review comments apply to.

The versions of the paper that follow are the versions that were submitted during the peer review process. They are not the versions of record or the final published versions. They should not be cited or distributed as the published version of this manuscript.

BMJ Open is an open access journal and the full, final, typeset and author-corrected version of record of the manuscript is available on our site with no access controls, subscription charges or pay-per-view fees (http://bmjopen.bmj.com).

If you have any questions on BMJ Open's open peer review process please email info.bmjopen@bmj.com

BMJ Open

RISK-STRATIFIED RANDOMISED CONTROLLED TRIAL IN PAEDIATRIC CROHN'S DISEASE: METHOTREXATE VERSUS AZATHIOPRINE OR ADALIMUMAB FOR MAINTAINING REMISSION IN PATIENTS AT LOW OR HIGH RISK FOR AGGRESSIVE DISEASE COURSE, RESPECTIVELY – A TREATMENT STRATEGY: THE REDUCE-RISK IN CD PIBD TRIAL

Journal:	*BMJ Open*
Manuscript ID	bmjopen-2019-034892
Article Type:	Protocol
Date Submitted by the Author:	09-Oct-2019

Complete List of Authors:	Harris, Rachel; Royal Hospital for Children Glasgow<br>Aloi, Marina; Sapienza University of Rome, Paediatric Gastroenterology and Liver Unit<br>de Ridder, Lissy; Erasmus MC / Sophia Childrens Hospital, Paediatrics<br>Croft, Nicholas; Barts and The London School of Medicine and Dentistry<br>Koletzko, Sibylle; Dr. v. Hauner Children's Hospital, Pediatric Gastroenterology and Hepatology; Collegium Medicum University of Warmia and Mazury, Department of Pediatrics<br>Levine, Arie; Tel Aviv University, Edith Wolfson Medical Center,<br>Turner, Dan; Hebrew University of Jerusalem<br>Veereman, Gigi; UZBrussels-VUB, Pediatric GI; Free University Brussels, University Hospital, Veereman<br>Bigot, Laetitia; Hôpital universitaire Necker-Enfants malades, PIBD-Net<br>Ruemmele, Frank; Hôpital universitaire Necker-Enfants malades, Service de Gastroentérologie pédiatrique; Université Paris Descartes<br>russell, richard; Royal Hospital for Children Glasgow, ; RHC Glasgow

Keywords:	Inflammatory bowel disease < GASTROENTEROLOGY, Paediatric gastroenterology < GASTROENTEROLOGY, Clinical trials < THERAPEUTICS

SCHOLARONE™
Manuscripts

BMJ

I, the Submitting Author has the right to grant and does grant on behalf of all authors of the Work (as defined in the below author licence), an exclusive licence and/or a non-exclusive licence for contributions from authors who are: i) UK Crown employees; ii) where BMJ has agreed a CC-BY licence shall apply, and/or iii) in accordance with the terms applicable for US Federal Government officers or employees acting as part of their official duties; on a worldwide, perpetual, irrevocable, royalty-free basis to BMJ Publishing Group Ltd ("BMJ") its licensees and where the relevant Journal is co-owned by BMJ to the co-owners of the Journal, to publish the Work in this journal and any other BMJ products and to exploit all rights, as set out in our licence.

The Submitting Author accepts and understands that any supply made under these terms is made by BMJ to the Submitting Author unless you are acting as an employee on behalf of your employer or a postgraduate student of an affiliated institution which is paying any applicable article publishing charge ("APC") for Open Access articles. Where the Submitting Author wishes to make the Work available on an Open Access basis (and intends to pay the relevant APC), the terms of reuse of such Open Access shall be governed by a Creative Commons licence – details of these licences and which Creative Commons licence will apply to this Work are set out in our licence referred to above.

Other than as permitted in any relevant BMJ Author's Self Archiving Policies, I confirm this Work has not been accepted for publication elsewhere, is not being considered for publication elsewhere and does not duplicate material already published. I confirm all authors consent to publication of this Work and authorise the granting of this licence.

For peer review only

RISK-STRATIFIED RANDOMISED CONTROLLED TRIAL IN PAEDIATRIC CROHN'S DISEASE: METHOTREXATE VERSUS AZATHIOPRINE OR ADALIMUMAB FOR MAINTAINING REMISSION IN PATIENTS AT LOW OR HIGH RISK FOR AGGRESSIVE DISEASE COURSE, RESPECTIVELY – A TREATMENT STRATEGY: THE REDUCE-RISK IN CD PIBD TRIAL

Rachel E. Harris[1]; Marina Aloi[2]; Lissy de Ridder[3]; Nicholas M Croft[4]; Sibylle Koletzko[5,6]; Arie Levine[7]; Dan Turner[8]; Gigi Veereman [9]; Laetitia Bigot[10]; Frank M. Ruemmele[11,12*]; Richard K. Russell[1*]; on behalf of the PIBD SETQuality consortium and PIBDnet

1. Department of Paediatric Gastroenterology, Hepatology and Nutrition, Royal Hospital for Children, Glasgow, UK
2. Paediatric Gastroenterology and Liver Unit, Department of Paediatrics, Sapienza University of Rome, Rome, Italy
3. Erasmus MC/Sophia Children's Hospital, Rotterdam, The Netherlands
4. Barts and the London School of Medicine, Queen Mary University of London, London, UK.
5. Department of Pediatrics, Dr. von Hauner Children's Hospital, University Hospital, LMU Munich, Munich, Germany
6. Department of Pediatrics, Gastroenterology and Nutrition, School of Medicine Collegium Medicum University of Warmia and Mazury, Olsztyn, Poland
7. Paediatric Gastroenterology and Nutrition Unit, Tel Aviv University, Edith Wolfson Medical Center, Holon, Israel
8. Institute of Paediatric Gastroenterology, Shaare Zedek Medical Center, The Hebrew University of Jerusalem, Jerusalem, Israel

For peer review only

9. Department of Paediatric Gastroenterology and Nutrition, UZ Brussel - Vrije Universiteit Brussel, Brussels, Belgium

10. Pediatric Inflammatory Bowel Disease network (PIBD-net), Hôpital Necker Enfants Malades, Paris, France

11. Université Paris Descartes, Sorbonne Paris Cité, Paris, France

12. Assistance Publique-Hôpitaux de Paris, Hôpital Necker-Enfants Malades, Service de Gastroentérologie pédiatrique, Paris, France

* FMR and RKR share senior authorship

Corresponding Author:

Professor Richard K. Russell

Department of Paediatric Gastroenterology, Hepatology and Nutrition

Royal Hospital for Children

1345 Govan Road

Glasgow

G51 4TF

UNITED KINGDOM

Telephone: 0044 141 451 6543

Email: richardrussell@nhs.net

Key Words: Paediatric Gastroenterology, Inflammatory Bowel Disease, Crohn's Disease, Immunomodulators

For peer review only

ABSTRACT

Introduction

Immunomodulatory medications such as thiopurines (azathioprine (AZA)/6-mercaptopurine (6MP)), methotrexate (MTX) and adalimumab (ADA) are well established for maintenance of remission within paediatric Crohn's disease (CD). It remains unclear which maintenance medication should be used first-line in specific patient groups.

Aims

To compare the efficacy of maintenance therapies in newly diagnosed CD based upon stratification into high and low risk groups for severe CD evolution; MTX versus AZA/6MP in low-risk and MTX versus ADA in high-risk patients. Primary end point: sustained remission at 12 months (weighted paediatric Crohn's disease activity index ≤12.5 and C-reactive protein ≤1.5-fold upper limit) without relapse or ongoing requirement for EEN/steroids 12 weeks after treatment initiation.

Methods and Analysis

REDUCE-RISK in CD is an international multicentre open-label prospective randomised controlled trial funded by EU within the Horizon2020 framework (grant number 668023). Eligible patients (aged 6-17 years, new-onset receiving steroids or EEN for induction of remission for luminal +/- perianal CD; are stratified into low and high-risk groups based upon phenotype and response to induction therapy.

For peer review only

Participants are randomised to one of two treatment arms within their risk group: low-risk patients to weekly subcutaneous MTX or daily oral AZA/6MP, and high-risk patients to weekly subcutaneous MTX or fortnightly ADA. Patients are followed up for 12 months at pre-specified intervals. Electronic case report forms are completed prospectively. The study aims to recruit 312 participants (176 low-risk; 136 high-risk).

Ethics and Dissemination

ClinicalTrials.gov Identifier: (NCT02852694), authorisation and approval from local ethics committees have been obtained prior to recruitment. Individual informed consent will be obtained prior to participation in the study. Results will be published in a peer-reviewed journal with open access.

Registration Details

NCT02852694; pre-results.

Article Summary

- This study is the first international prospective RCT comparing three different immunomodulatory medications for maintenance of remission in newly diagnosed CD based upon a risk stratification protocol.
- This study may better define the most appropriate first-line immunomodulatory medication to be used in specific subsets of CD patients requiring immunomodulatory maintenance therapy.
- An ancillary study will compare outcomes in ADA treated patients from inclusion (TOP-Down) versus patients switched to ADA due to failure of immunomodulator therapy (STEP-Up).

For peer review only

- Therapeutic efficacy will be supported by drug levels, pharmacogenomics and microbiome analysis as secondary outcomes.
- Inability to blind patients, their relatives or treating physicians to treatment due to ethical issues and differences between medication administration routes serves as a limitation to this study.
- Blinding of an alternative clinician to assess disease activity during study visits may prove practically difficult in smaller centres.

For peer review only

INTRODUCTION

Crohn's disease (CD) the most common form of inflammatory bowel disease (IBD) in children is a chronic disorder with the potential to affect the whole gastrointestinal tract. The aim of CD treatment is to control active inflammation and achieve bowel healing; chronic and uncontrolled CD results in poor outcomes for patients, including

reduced quality of life, recurrent hospitalisation and potential need for surgical intervention.[1] Treatments for CD are categorised into those which induce remission (such as steroids[1,2] or exclusive enteral nutrition (EEN)[1,3] and those which maintain remission. Immunomodulatory medications are a mainstay of maintenance treatment in IBD; with the efficacy of thiopurines (e.g. azathioprine (AZA) and 6-mercaptopurine (6MP))[4,5,6] and methotrexate (MTX)[7,8,9,10] well established. Anti-tumour necrosis factor (anti-TNF) therapies (infliximab[11,12] and adalimumab (ADA)[13,14]) including their biosimilars are used in those patients refractory to "traditional" induction or maintenance treatment.

Due to a lack of treatment strategy trials within the paediatric IBD (PIBD) population however, it remains unclear which of the aforementioned maintenance therapies should be used first-line in individual patients. Randomised controlled trials comparing the use of MTX with thiopurines for maintenance of remission failed to show a significant difference in efficacy between the two.[15,16, 17] A Cochrane review in adults with quiescent CD highlighted the lack of adequately powered trials necessary in order to determine the efficacy and safety of thiopurines compared to other maintenance therapies[4, 10]. The RISK study demonstrated improved clinical and growth-based outcomes at 1 year with anti-TNF monotherapy in comparison with immunomodulatory monotherapy; however further investigation into which specific patients are most likely to benefit from these therapies is still required.[18] There is a clear disparity between North America and Europe in terms of which form of immunosuppression first with both concerns about efficacy and safety lying behind these thus there is an urgent need for a head to head study in children to help inform the first choice of immunosuppression.

For peer review only

Stratifying patients by risk for complex or severe CD may allow pre-emptive direction of maintenance strategy and potentially an early reduction in disease burden with subsequent improvement in long-term outcomes. The adult IBD Ahead initiative highlighted young age at diagnosis as a risk factor for severity of CD evolution[19]; all patients diagnosed within paediatric services would therefore be considered 'high risk'. Paediatric consensus guidelines suggest that paediatric CD patients at 'high risk for poor outcome' should receive early therapy optimisation to modify progression of their disease.[1] The guidelines list specific features which may be considered predictive for poor outcome in paediatric CD (see Table 1).[1] Patients deemed at high risk for complex disease or poor outcome may benefit from a 'Top-down' approach as the TISKids aims to investigate[21].

Therefore the PIBDnet consortium recognised the urgent need to investigate the efficacy and safety of immunomodulatory medications and to stratify whether a top-down approach was superior to a traditional 'step-up' for paediatric patients deemed at high risk for rapidly complicated disease course. REDUCE-RISK in CD is a randomised controlled trial (RCT) which aims to compare the effectiveness of immunomodulatory medications for maintenance of remission in newly diagnosed CD based upon risk stratification specifically, the effectiveness of MTX versus AZA/6MP for maintenance of remission who are low risk for rapidly progressive disease and the effectiveness of MTX versus ADA in a high risk group.

METHODS AND ANALYSIS

Study Design

For peer review only

We designed an international multicentre open-label prospective RCT with 4 treatment arms as shown in Figure 1. Following screening and consent, eligible patients are stratified into low and high-risk groups based upon phenotype and disease response to induction therapy (Table 1). Patients are then randomised to one of two arms within their risk group: with low risk patients receiving either weekly subcutaneous MTX or daily oral AZA/6MP, and high-risk patients receiving either weekly subcutaneous MTX or fortnightly subcutaneous ADA.

For peer review only

Study End Points

Patients are followed up for 12 months post randomisation. The primary end point of the study is sustained steroid or EEN-free remission at 12 months, defined as weighted Paediatric Crohn's Disease Activity Index (wPCDAI) ≤12.5 and C-reactive protein (CRP) ≤1.5-fold upper limit without a relapse or need for EEN/steroids since week 12.

Secondary end points include comparison of time to first relapse, remission at 12 weeks, growth, adverse events, health related quality of life and patient reported outcomes between the two treatment arms within each risk group, but also between low and high risk MTX treated patients. The study also aimed to evaluate clinical predictors for response, including genomic and serological markers and results of drug monitoring (MTX and ADA concentrations) metabolites (6-thioguanine (6-TG) and 6-methylmercaptopurine (6-MMP) in AZA/6MP) and anti-drug antibodies (ADA) in relation to adherence, toxicity and response. The ancillary study additionally aimed

to evaluate the efficacy of ADA in patients treated from inclusion (Top-down) versus patients switched to ADA due to immunomodulator failure (Step-up). Further outcome measures are detailed in Box 1.

Box 1: Study endpoints

Primary Endpoint
- Sustained steroid/EEN-free remission at month 12, where sustained remission is defined as wPCDAI ≤12.5 and CRP ≤1.5 times the upper limit without a relapse or need for EEN/steroids since week 12.

Secondary Endpoints*:*
Comparing the following within 1) the two treatment arms per risk group; 2) methotrexate treatment between high and low risk groups; and 3) TOP-Down adalimumab (high risk group) versus STEP-Up adalimumab (ancillary study):
- Rate of clinical remission at month 12 (physician global assessment (PGA), wPCDAI, paediatric Crohn's disease activity index (PCDAI))
- Relapse free remission with normal CRP at month 12
- Relapse free remission with normal CRP and faecal calprotectin <300 at month 12
- Remission at week 12
- Time to first relapse after week 12
- Faecal calprotectin values at visits 1, 2, 4 and 6 (respectively at month 0, 2, 6 and 12)
- Dropout rates
- Adverse drug event rate
- Height velocity and z-score at baseline and 52 weeks
- Quality of life as measured by the IMPACT 3 questionnaire completed at each study visit
- Health economic evaluation at all visits (forms EQ-5D-Y proxy 1, EQ-5D-Y and EQ-5D-5L, WPAI:CD Caregiver, School Attendance start of the research and follow up visits)

Eligibility Criteria and Recruitment

Full eligibility criteria for the study are listed in Box 2. Patients are eligible if aged 6-17 years with new-onset (<6 months) treatment naïve luminally active and/or

perianal fistulising CD diagnosed as per revised Porto criteria[22] receiving steroids or EEN for induction of remission with wPCDAI >40 or CRP >2 times upper limit of normal at diagnosis. Informed consent from must be obtained prior to participation in the study. Patients are excluded in cases of previous use of IBD related medications; pregnancy or refusal to use contraceptives; disease requiring surgery; contraindications to study medication; exposure to live vaccine within 3 weeks; oral anticoagulant or anti-malarial use; current or previous malignancy; significant infection; or significant comorbidity.

For peer review only

Box 2: Eligibility criteria

Inclusion Criteria

- Patients aged 6-17 years with new-onset (<6 months) treatment naïve active luminal and/or perianal fistulising Crohn's disease diagnosed using established criteria[22] requiring steroids or EEN for induction of remission
- wPCDAI >40 or CRP >2 times upper limit of normal at diagnosis
- Luminal active Crohn's disease (B1) with or without B2 and/or B3 disease behaviour as per Paris classification [23]
- Signed informed consent

Exclusion Criteria

- wPCDAI <42.5 at diagnosis, except where CRP >2 times upper normal limit
- Lack of induction therapy with steroids or EEN
- Previous therapy with any IBD-related medication other than induction therapy as detailed within this protocol with the exception of 5-aminosalicylic acid (5ASA) preparations
- Pregnancy or refusal to use contraceptives during the study period in pubertal patients unless absolute abstinence is confirmed at each study visit
- Lactating mothers
- Perianal fistulising disease requiring surgical therapy
- Patients homozygous for thiopurine methyltransferase (TPMT) mutations or those with TPMT activity <6 nmol/h/ml erythrocytes or <9nmol 6MTG/g Hb/h, unless they qualify as high-risk patients
- Evidence of un-drained and un-controlled abscess/phlegmon
- Contraindication to any drugs used in the trial (including intolerance/hypersensitivity or allergy to study drugs (thiopurines, methotrexate or adalimumab))
- Current or previous malignancy
- Serious comorbidities (e.g. renal insufficiency, hepatitis, respiratory insufficiency) which may interfere with drug therapy or interpretation of outcome parameters or will make it unlikely that the patient will complete the trial.
- Infection with mycobacterium tuberculosis, hepatitis B or C, human immunodeficiency virus (HIV)
- Moderate to severe heart failure (New York Heart Association class III/IV)
- Oral anticoagulant therapy, anti-malarial therapy
- Live vaccine exposure (including yellow fever) less than 3 weeks prior to inclusion

Screening Visit (Visit 0)

The screening visit allows for assessment of eligibility for inclusion in the study, evaluation of the patient's response to induction therapy if already commenced,

commencement of induction therapy where not commenced, and acquisition of consent and assent.

Induction Therapy

All enrolled patients receive either corticosteroids or exclusive enteral nutrition (EEN) as induction as determined by the clinical team and the patient/caregiver. For EEN any balanced formula (polymeric or elemental) administered orally or via nasogastric tube is permitted and should be prescribed for 6-8 weeks. Tapering of steroids is at the discretion of the prescribing clinician. Adaptation of induction therapy (e.g. dose increase of steroids or return to EEN) or crossover from one induction therapy to the other is permitted in order to achieve remission, however patients must have discontinued their induction therapy by week 12. If induction therapy is not discontinued by week 12 the patient is considered a treatment failure, with protocol for this detailed below.

<u>Inclusion Visit and Risk Group Allocation (week 5 +/- 3 weeks; visit 1)</u>

In order to incorporate response to initial induction therapy within the risk stratification criteria, inclusion and risk group allocation is performed at week 5 +/- 3 weeks of induction therapy. Data from the screening visit is reviewed with ineligible patients excluded, and patients are then stratified into the high or low risk group (Table 1) based upon the ECCO/ESPGHAN consensus guidelines[1]. Patients with

perianal fistulising disease at diagnosis are auto-allocated to the high-risk group regardless of other factors at inclusion visit. All other patients are allocated to the low risk group. Patients with low thiopurine methyltransferase (TPMT) activity or homozygous mutations are excluded should they be categorised as low risk.

DEFINING HIGH RISK CROHN'S DISEASE PATIENTS	
**ECCO/ESPGHAN CONSENSUS GUIDELINES[1]**	**MODIFIED STUDY CRITERIA**
Severe perianal disease	Complex perianal fistulising disease phenotype
Extensive (pan-enteric) disease; deep colonic ulcers on endoscopy	Panenteric disease phenotype (defined as L3 with L4b as per Paris classification[23] or L3 with deep ulcers in the duodenum, stomach or oesophagus not related to non-steroidal anti-inflammatory medications or Helicobacter pylori)
	Overall cumulative disease extent of >/=60cm
Stricturing and penetrating disease at onset	B2, B3 or B2B3 disease behaviour [21]
Marked growth retardation >-2.5 height Z scores	Severe growth impairment (height z-score <-2 or crossing >/= 2 centiles) likely related to Crohn's disease
Persistent severe disease despite adequate induction therapy	Hypoalbuminemia (<30g/L), elevated CRP (at least 2 times upper limit of normal range), or wPCDAI>12.5 despite at least 3 weeks of optimized induction therapy with steroids or EEN
Severe osteoporosis	Not included

Table 1 – Definition of high-risk patients based upon ECCO/ESPGHAN consensus guidelines[1] (ECCO – European Crohn's and Colitis Organisation; ESPGHAN – European Society for Paediatric Gastroenterology, Hepatology and Nutrition; CRP – C-reactive protein; wPCDAI – weighted Paediatric Crohn's Disease Activity Index; EEN – exclusive enteral nutrition)

For peer review only

Randomisation and Treatment Allocation

Randomisation is undertaken following allocation to high or low risk group at week 5 +/- 3 weeks. This process utilises an integrated module within the electronic case report form (CRF) system. Within both the high and low risk groups patients are 1:1 randomised to MTX versus ADA or AZA/6MP respectively in blocks of four stratified by EEN or steroid induction therapy. Code for randomisation is prepared and held by the central coordinating site and site co-ordinators are then informed of the results. Immunomodulator or biologic therapy should be commenced within 2 weeks of randomisation as per the protocol outlined in Table 2.

AZA/6MP and MTX are prescribed and dispensed according to local guidelines. ADA (Humira ®) is provided by AbbVie. Co-interventions are prohibited.

For peer review only

	Therapy	Route	Dose	Notes
**LOW RISK PROTOCOL**	Methotrexate	SC	15mg/m^2 body surface area weekly (max dose 25mg)	Ondansetron 4-8mg orally 1 hour pre injection and folic acid 15mg (5mg in patients <20kg) 3 days post injection are recommended for all patients
	**VERSUS**			
	Azathioprine	PO	2.5mg/kg (rounded down to nearest 12.5mg)	Half calculated dose for TPMT heterozygotes/activity 6-9nmol/h/ml
	**OR**			
	6-Mercaptopurine	PO	1.5mg/kg (rounded down to nearest 12.5mg)	Half calculated dose for TPMT heterozygotes/activity 6-9nmol/h/ml
**HIGH RISK PROTOCOL**	Methotrexate	SC	15mg/m^2 body surface area weekly (max dose 25mg)	Ondansetron 4-8mg orally 1 hour pre injection and folic acid 15mg (5mg in patients <20kg) 3 days post injection are recommended for all patients
	**VERSUS**			
	Adalimumab<br><br>(Humira ®)	SC	160mg then 80mg after 2 weeks then 40mg every 2 weeks thereafter (patients >35kg)<br><br>120mg then 80mg after 2 weeks then 40mg every 2 weeks thereafter (patients 25-35kg)<br><br>80mg then 40mg after 2 weeks and 20mg every 2 weeks thereafter (patients <25kg)	

Table 2: Medication protocol for low and high-risk patients following randomisation

(TPMT – thiopurine methytransferase)

For peer review only

Follow Up Visits (Visit 2, 3, 4, 5 and 6)

Patients are followed up at pre-specified intervals (Figure 1) with a window of +/- 2 weeks. A telephone call is undertaken at week 4 following initiation of induction in order to support patient compliance with induction regime and advise weaning where appropriate. Data as described in Box 3 are collected at each consultation. Patients' compliance with therapy is determined at each face-to-face follow up visit by pill and vial counts plus by patients' reporting.

Box 3: Standard requirements for each study visit

- An explicit history of illness since last visit, including review of symptoms, medications (including compliance check) and adverse events.
- Physical examination
- wPCDAI, PGA and PCDAI scoring
- Anthropometrics (height measured using a calibrated wall mounted stadiometer)
- Blood tests
  - White blood cells
  - Absolute neutrophil count
  - Haemoglobin
  - Haematocrit
  - Platelet count
  - Erythrocyte sedimentation rate (ESR)
  - C-reactive protein (CRP)
  - Amylase
  - Albumin
  - Aspartate transaminase (AST)
  - Alanine transaminase (ALT)
  - Conjugated bilirubin
  - Gamma glutamyl transferase (GGT)
- Stool samples for faecal calprotectin and microbiome analysis
- Health economic parameters (EQ-5D-Y proxy 1; EQ-5D-Y; EQ-5D-5L; WPAI:CD; school attendance questionnaire)
- Quality of life evaluation (IMPACT 3)
- Urine human chorionic gonadotropin (hCG) in all female patients of child-bearing potential
- Confirmation of contraception use or of absolute abstinence in all patients

Remission is defined as wPCDAI</=12.5, normal CRP (</= 1.5 times upper normal range) and being free of steroids or EEN. Once remission is achieved and induction therapy is discontinued, a patient is considered to be failing treatment or experiencing a relapse in the following circumstances:

- wPCDAI >40
- CRP >2 times upper normal limit in the absence of any clear infectious process
- wPCDAI >12.5 but <40 and/or CRP >1.5 times but <2 times over upper normal limit at 2 consecutive visits within 2-8 weeks
- Development of CD related complications e.g. fistulisation
- Requirement for additional CD-specific medication/surgery since last study visit

A patient will also be considered a treatment failure should induction therapy be continued at week 12. In addition, the treating clinician may escalate treatment at any time point independent of wPCDAI score if it is felt that the patient is experiencing a relapse.

For peer review only

Dose Optimisation and Therapeutic Drug Monitoring

Drug monitoring is undertaken as detailed below. In addition to this, samples for drug monitoring should be collected at the time of medication cessation in the event of drug discontinuance due to adverse effect or loss of response. Potential adaptations to therapies which may be made at specific follow up visits are detailed in Box 4.

Box 4 – Potential adaptations to therapies at follow up visits

Month 2 (Visit 2)

- Failure to discontinue induction therapy by week 12
    - Offer switch to the ancillary study (ADA STEP-up) to those prescribed MTX or AZA/6MP, or an increase in dose frequency to weekly in those prescribed ADA
    - Alternatively, the patient may leave the study and receive therapies as per the discretion of the treating clinician.

Months 4, 6, 9 and 12 (Visits 3, 4, 5 and 6)

- Thiopurine non response
    - Protocol as per metabolite levels (detailed in Table 3)
- Thiopurine intolerance (except pancreatitis)
    - Switch to alternate thiopurine (AZA to 6MP or vice versa) or split dose to provide twice daily (BD) dosing
- Thiopurine failure (any exacerbation despite dose optimisation/pancreatitis/cytopaenia)
    - Offer switch to ancillary study (ADA STEP-up) or exit study
- MTX intolerance or failure (any exacerbation or elevation of liver enzymes as detailed below)
    - Offer switch to ancillary study (ADA STEP-up) or exit study
- ADA failure (any exacerbation)
    - Increase frequency to weekly dosing

Azathioprine

RESULT	ACTION
6-TG <150	Consider non-compliance; repeat sample at subsequent visit and increase dose if low 6-TG confirmed (+25mg or +12.5mg if dose <50mg)
6-TG 150-800	No adaptation
6-TG >800	Decrease dose if repeat sample at subsequent visit confirms high 6-TG (-25mg or -12.5mg if dose <50mg)
6-MMP >8000 or signs of hepatotoxicity	Stop medication – switch to ancillary study<br><br>Erythrocyte lysate sample frozen at -80C and shipped to central lab at end of study for thiopurine nucleotides

Table 3 – Azathioprine dose adjustments based upon metabolite levels

TPMT genotype or phenotype at screening determines the initial dose of AZA/6MP; and measurement of thiopurine metabolites (6-thioguanine (6-TG) and 6-methylmercaptopurine (6-MMP)) at visit 2 determines requirement for subsequent dose adjustment performed according to the recommendations in Table 3. Where possible thiopurine metabolites are measured locally; central lab measurements are provided for centres where this is unavailable.

At visit 2 a urine sample for TPMT metabolite determination and an erythrocyte lysate sample for quantification of Thiopurine Nucleotides by Liquid Chromatography-Tandem Mass Spectrometry should be frozen at -80°C and shipped on dried ice to the central lab at the end of the study. At each visit from visit

For peer review only

2 to 6, an additional EDTA blood sample will be collected for further 6-TG and 6-MMP testing and frozen at -80C.

Methotrexate

Washed erythrocyte for MTX levels will be obtained at visits 2, 4 and 6 and stored frozen at local centres. These samples will be sent on dry ice for central analysis to evaluate response to therapy and adverse effects in relation to drug levels.

Adalimumab

Adalimumab trough levels are measured after 3 injections of maintenance therapy (e.g. at visit 2) within the local laboratory (central lab testing available if local lab testing is unavailable). Dosing interval may be shortened to weekly in the event of low ADA levels (<8 mcg/ml) and negative ADA antibodies. Further samples should be obtained at visits 3, 4, 5 and 6 and should be frozen for later analysis within the central lab.

Pharmacogenetics

DNA for pharmacogenetics should be taken from patients randomised to MTX or AZA/6MP for multiplex genotyping of polymorphism related to drug metabolism to evaluate safety and response to therapy. Analysis will be performed at the end of the study, or earlier in those patients showing toxicity.

For peer review only

Ancillary Study

Patients unable to discontinue induction therapy or those randomised to thiopurine or MTX therapy who experience treatment failure may be invited to participate in the ancillary study (STEP-up ADA) until visit 5. Any initial maintenance therapy will be stopped and induction and maintenance regime for ADA as previously described will be commenced. Up to 3 additional study visits at 3-month intervals will be offered to these patients in order to obtain 12 months of follow up. A maximum of 68 patients can participate in this ancillary study allowing a 1:1 comparison of TOP-down ADA to STEP-up ADA therapy.

Unscheduled Visits

Unscheduled visits may be arranged based upon clinical requirements. As for scheduled visits per protocol treatment adaptions are possible if intolerance or failure of the study drug is detected. Subsequent scheduled visits will not be changed after an unscheduled visit.

Treatment Discontinuation

Patients who discontinue treatment before completing 12 months of study drug within either the main study or the ancillary study will receive a single follow-up visit. This will be either 12 months after the commencement of study treatment or at the point of inclusion in the ancillary study.

Modifications to the protocol while the study is being conducted will be relayed to all site staff by email and then onto their relevant ethical and regulatory boards. The current manuscript is based on protocol 5.1 last modified 28th May 2019.

For peer review only

Allocation Concealment and Blinding

For ethical reasons we decided against a double dummy design for blinding the patient, parents and care givers. Due to the differences in medication administration route and the significant nausea commonly associated with MTX blinding of the allocation to the patients, their families or their physicians is not possible. Where possible however, blinding of an alternative clinician to score the wPCDAI, PCDAI and PGA at each study visit should occur (prospective randomized open blind end-point (PROBE) evaluation).

Safety

The external and independent Advisory Board of PIBDSETQuality serves as an independent Data and Safety Monitoring Board; it meets at pre-specified intervals with access to all data within the study. The principal investigator at each site is responsible for reporting any safety issues (adverse events, serious adverse events (SAEs), suspected unexpected serious adverse reactions), drop-outs, or any new information which may impact the study in any way. The principal investigator shall report to the sponsor all SAEs experienced by a study subject receiving an Adalimumab(Humira) within 24 hours of learning of the event regardless of the relationship of the event to the product. All SAEs are immediately sent to AbbVie pharmacovigilance by the sponsor. SAEs will be followed from the date of patient's signature of informed consent, until complete resolution or 30 days after the end of the study/patient's final study visit.

Box 5 – Criteria for premature termination of study treatment or participation

- Pregnancy at any stage
- Treatment failure as per protocol
- Failure to tolerate allocated treatment or alternatives as listed within the protocol
- Significant drug related side effects manifesting as significantly abnormal bloods results or adverse effects based upon the clinical judgement of the treating physician
- Request of participant to be withdrawn from treatment
- The judgement of the treating physician being that it is in the best interests of the participant to withdraw from study treatment
- Loss of participant to follow up
- Patient death

Participants may withdraw consent for further participation or data collection at any time without giving reason and without prejudicing further care or treatment; and will be permanently withdrawn from study treatment in the event of any of the situations outlined in Box 5. Patients should be provided with a study alert card for use in the event of an emergency.

Biochemical markers are monitored with a clearly defined protocol for adjustments to therapy based on abnormal results (e.g. neutropenia, pancreatitis, elevated liver enzymes).

Data Collection, Management and Monitoring

Patient CRFs are completed in a prospective manner using an electronic web-based system designed specifically by PIBDnet for this trial. In order to maintain data

For peer review only

security and integrity, the web-based data entry will be linked to a password secured Microsoft Access database, where data will be stored until time of analysis. Files will be saved on a code secured net-drive and backed-up following each data entry on a disk locked in a cabinet. Patients will be identified only by a study code assigned at the point of enrolment. Code of patient identifiers will be kept at each participating site. Handling of patient-identifiable is compliant with the legislation of each participating centre and the European General Data Protection Regulation (GDPR). Investigators will be invited to fax or email the paper source document to the coordinating site on a random basis to allow appropriate monitoring. Access to data with detailed information on study outcomes will be made available to other research groups on request and at the discretion of the principal investigators.

Monitoring arrangements are in place for all sites after initial site initiation. The monitoring visits will occur regularly partly dependant on recruitment rate at individual sites. The monitoring is perfomed usually by someone external to the clinical team.

Analysis and Statistical Methods

Descriptive statistics (mean, median, standard deviation, standard error, quartiles, minimum, maximum, and two-sided 95% confidence limits of mean and median) will be presented for each treatment of the low and high risk paediatric CD groups and, where applicable, for the paired difference of each patient. Frequency tables will be presented where applicable.

Primary Analysis

For peer review only

Difference in the 12-month steroid/EEN free sustained remission rates between the treatment groups will be undertaken using Chi square test. Mantel Haenzel test will be used to combine data from all participating sites.

Secondary Analyses

Chi-square tests or Fisher's exact tests will be used to compare rates of remission, steroid intake, dropout and serious adverse events between the two arms of each risk group and between the low and high risk MTX groups. Logistic regression analyses may be performed to adjust for any imbalances in baseline covariates. To compare time to disease flare between the arms of each risk group and between high and low risk MTX groups, a Kaplan–Meier survival estimate will be used and the log-rank test of equality over strata. A Cox proportional hazard model will be constructed to obtain a hazard ratio after validation of the proportionality assumption and adjusting for possible confounding variables (including age and disease duration). Student's t tests or Wilcoxon rank sum tests will be used to compare growth, steroid dose, adverse events, changes in quality of life and patient reported outcomes between the two arms of each risk group and between the high and low risk MTX groups. The predictive value of faecal calprotectin levels, CRP, serum tests or other clinical predictors for response (including genomic and serological markers) will be assessed for each arm of the study using sensitivity, specificity, negative and positive predictive values or area under the ROC curve. Multivariate logistic regression analyses will then be performed.

For peer review only

Analyses will be performed using the R software (http://cran.r-project.org). All comparisons will be made using a 2-sided significance level of 0.05.

Sample Size Considerations

Estimated remission rates are based on recent analysis from the RISK study[18], indicating an advantage of early anti-TNF introduction over immunomodulator therapy. For the low risk group, it was hypothesized that 48% of children will be in remission at 12 months for the AZA/6MP arm versus 70% for the MTX arm. On the basis of this data with an alpha risk of 5% and a power of 80% a sample requirement of 88 patients per arm was calculated assuming a 10% loss of follow up. For the high-risk group, it was hypothesised that 40% of children will be in remission at 12 months for the MTX arm versus 65% for the adalimumab arm. To detect this difference with an alpha risk of 5% and a power of 80%, a sample size of 68 participants is necessary, again assuming a 10% loss of follow-up. In total 312 participants will be included in the study (176 low-risk group; 136 high risk group).

Patient and Public Involvement

Patients were not involved in the development of this study; however, the French patient charity AFA Crohn, RCH, France was involved in study design and critically reviewed and commented upon all aspects of the trial.

For peer review only

Discussion

REDUCE-RISK in CD is the first multicentre international RCT aiming to compare three different medication strategies for maintenance of remission in newly diagnosed CD based upon a risk stratification protocol. During the 12-month follow up period the effects of the differing management strategies will be assessed via data collected and outcome measures as defined above in order to analyse the efficacy and safety of each medication and better define the most appropriate first-line maintenance immunomodulatory medication to be used in specific subsets of CD patients. As a group we speculatively hypothesise that MTX will be superior to thiopurines for maintaining remission in CD in the low risk group although in the absence of head to head studies prior to this one this study will provide data to address this. We also hypothesise that ADA will be superior to MTX in the high-risk group based upon the results from the RISK study.[18] In addition to this, the ancillary study will compare outcomes in ADA treated patients from inclusion (Top-down) versus patients switched to ADA due to failure of immunomodulator therapy (Step-up), with the potential to stratify which patients might benefit from such a top-down treatment strategy.

The design and completion of interventional studies in PIBD is a recognised challenge between rigorous study design methodology and pragmatic considerations around feasibility and completion within a paediatric dataset.[24] This particular study is limited by the inability to blind the treatment allocation to the patients, their families

For peer review only

or their treating physician due to the differences in medication administration route and the side effects commonly associated with the study medication. Although the protocol advises that where possible blinding of an alternative clinician to score disease assessment at each study visit should occur in order to obtain prospective randomized open blind end-point (PROBE) evaluation this may be practically difficult in smaller centres where staff are familiar with the majority of their patient cohort.

ETHICS AND DISSEMINATION

The study is being conducted according to the principles of the Declarations of Helsinki and to date has been approved by all participating sites as listed within supplementary Table 1. Clinical trials authorisation and ethics approval has been obtained from the local ethics review committees of these participating nations and centres. The Standard Protocol Items: Recommendations for Interventional Trials (SPIRIT) guidelines[25] were adhered to in the production of the protocol for this trial (see uploaded material for details).

Consent

Patients and their caregivers are provided with study-specific information including an explicit description of the study outline and alternatives for participation. It is made clear to all patients approached that declining to participate in the study will not jeopardize the quality of subsequent care received. After a period of consideration, if agreeable, the patient's parent or caregiver is asked to sign consent forms with age-

For peer review only

appropriate assent obtained from the child where relevant (see appendix 1 for model consent forms). The signed forms are filed within the patient's medical record with a copy provided to the participant and their caregiver. Consent will be obtained by site staff with the relevant training and who are identified as assigned on the delegation log. Participants taking part in the ancillary study will not be re-consented.

Dissemination

Results of the study will be submitted for publication within a peer-reviewed journal. In accordance with the H2020 general grant agreement, the dissemination process will ensure open access to the scientific publications resulting from this project. Journal authorship guidelines will be adhered to and there are no plans to use professional writers.

AUTHOR CONTRIBUTIONS

R. Harris prepared the manuscript with comments and review from all authors. **RKR** and FMR **assisted in developing the original study protocol and** provided critical review of the manuscript.

All authors have assisted in developing the original study protocol and approved the uploaded draft.

As sponsor PIBDnet has full responsibility and control for the original study design, collection, management, analysis, and interpretation of data, including writing of the report and the decision where to submit the report for publication,

FUNDING

For peer review only

This work was supported as part of the PIBD-SETQuality (Paediatric Inflammatory Bowel Diseases Network for Safety, Efficacy, Treatment and Quality improvement of care) project funded by the European Commission Horizon 2020 - Research and Innovation Framework Programme (grant number 668023). ADA (Humira ®) is provided by AbbVie.

The main study sponsor is PIBDNet. PIBDNet is the EU legal representative for the study. The specific contact for the sponsor is Frank Ruemmele (Service de Gastro-entéroloegie, Hôpital Necker Enfants Maldes, 149 rue de Sèvres, 75015 Paris, France).

For peer

COMPETING INTERESTS

RKR is supported by an NHS Research Scotland Senior Research Fellowship, and has received speaker's fees, travel support, and/or participated in medical board meetings with Nestle, MSD Immunology, AbbVie, Dr Falk, Takeda, Napp, Mead Johnson, Nutricia & 4D Pharma. FMR has received speaker fees from Shering-Plough, Nestlé, MeadJohnson, Ferring, MSD, Johnson & Johnson, Centocor, AbbVie; has served as a board member for SAC:DEVELOP (Johnson & Johnson), CAPE (AbbVie), LEA (AbbVie); and has been invited to MSD France, Nestlé Nutrition Institute, Nestlé Health Science, Danone, MeadJohnson, Takeda, Celgene, Biogen, Shire, Pfizer, and Therakos. DT received consultation fee, research grant, royalties, or honorarium from Janssen, Pfizer, Hospital for Sick Children, Ferring, Abbvie, Takeda, Biogen, Atlantic Health, Shire, Celgene, Lilly, Neopharm, Roche. LdR received consultation fee, research grant, or honorarium from ZonMw, ECCO, Shire, Malinckrodt, Nestlé, Celltrion, Abbvie and Pfizer. MA received consultation fee and honorarium from Abbvie. SK received consultation fee, research grant, or

honorarium from Danone, Nestec-Nutrition, Abbvie, Takeda, Celgene, Shire, Pfizer, Biogaia, Janssen, Berlin-Chemie; Mead Johnson, Vifor, Pharmacosmos, ThermoFisher

Remaining authors: nil competing interests declared.

FIGURE LEGENDS

Figure 1 – Study Design of the REDUCE-RISK in CD trial

For peer review only

REFERENCES

1. Ruemmele FM, Veres G, Kolho KL et al. Consensus guidelines of ECCO/ESPGHAN on the medical management of pediatric Crohn's disease. *J Crohns Colitis*. 2014;8(10):1179-207.

2. Escher J. Budesonide versus prednisolone for the treatment of active Crohn's disease in children. *Eur J Gastroenterol Hepatol*. 2004;16(1):47-54.

3. Heuschkel R, Menache C, Megerian J et al. Enteral Nutrition and Corticosteroids in the Treatment of Acute Crohn's Disease in Children. *J Pediatr Gastroenterol Nutr.* 2000;31(1):8-15.

4. Chande N, Patton P, Tsoulis D et al. Azathioprine or 6-mercaptopurine for maintenance of remission in Crohn's disease. *Cochrane Database Syst Rev*. 2015;(10):CD000067

For peer review only

5. Markowitz J, Grancher K, Kohn N et al. A multicenter trial of 6-mercaptopurine and prednisone in children with newly diagnosed Crohn's disease. *Gastroenterology*. 2000;119(4):895-902.

6. D'Haens G, Geboes K, Rutgeerts P. Endoscopic and histologic healing of Crohn's (ileo-) colitis with azathioprine. *Gastrointest Endosc*. 1999;50(5):667-71.

7. Feagan BG, Fedorak RN, Irvine EJ et al. A comparison of methotrexate with placebo for the maintenance of remission in Crohn's disease. North American Crohn's Study Group Investigators. *N Engl J Med*. 2000;342(22):1627-32.

8. Feagan BG, Rochon J, Fedorak RN et al. Methotrexate for the treatment of Crohn's disease. The North American Crohn's Study Group Investigators. *N Engl J Med.* 1995;332(5):292-7.

9. Willot S, Noble A, Deslandres C. Methotrexate in the treatment of inflammatory bowel disease: An 8-year retrospective study in a Canadian pediatric IBD center. *Inflamm Bowel Dis*. 2011;17(12):2521-2526.

10. Patel V, Wang Y, MacDonald J et al. Methotrexate for maintenance of remission in Crohn's disease. *Cochrane Database Syst Rev*. 2014;(8):CD006884

For peer review only

11. Hyams J, Crandall W, Kugathasan S et al. Induction and maintenance infliximab therapy for the treatment of moderate-to-severe Crohn's disease in children. *Gastroenterology*. 2007;132(3):863-73.

12. Ruemmele F, Lachaux A, Cézard J et al. Efficacy of Infliximab in Pediatric Crohn’s Disease: A Randomized Multicenter Open-Label Trial Comparing Scheduled to On Demand Maintenance Therapy. *Inflamm Bowel Dis*. 2009;15(3):388-394.

13. Hyams JS, Griffiths A, Markowitz J et al. Safety and efficacy of adalimumab for moderate to severe Crohn's disease in children. *Gastroenterology*. 2012;143(2):365-74.e2.

14. Russell R, Wilson M, Loganathan S et al. A British Society of Paediatric Gastroenterology, Hepatology and Nutrition survey of the effectiveness and safety of adalimumab in children with inflammatory bowel disease. *Aliment Pharmacol Ther*. 2011;33(8):946-953.

15. Ardizzone S, Bollani S, Manzionna G et al. Comparison between methotrexate and azathioprine in the treatment of chronic active Crohn's disease: a randomised, investigator-blind study. *Dig Liver Dis*. 2003;35(9):619-27.

16. Oren R, Moshkowitz M, Odes S et al. Methotrexate in chronic active Crohn's disease: a double-blind, randomized, Israeli multicenter trial. *Am J Gastroenterol*. 1997;92(12):2203-9.

17. Maté-Jiménez J, Hermida C, Cantero-Perona J et al. 6-Mercaptopurine or methotrexate added to prednisone induces and maintains remission in steroid-dependent inflammatory bowel disease. *Eur J Gastroenterol Hepatol.* 2000;12(11):1227-1233.

18. Walters TD, Kim MO, Denson LA et al. Increased effectiveness of early therapy with anti-tumor necrosis factor-alpha vs an immunomodulator in children with Crohn's disease. *Gastroenterology*. 2014;146(2):383-91.

19. Torres J, Caprioli F, Katsanos KH et al. Predicting outcomes to optimize disease management in inflammatory bowel diseases. *J Crohns Colitis*. 2016;10(12):1385-1394

20. Levine A, Chanchlani N, Ziv-Baran T et al. Complicated disease and response to initial therapy predicts early surgery in paediatric Crohn's disease: results from the Porto Group GROWTH study. *J Crohns Colitis*. 2019;epub ahead of print.

21. Cozijnsen M, van Pieterson M, Samsom J et al. Top-down Infliximab Study in Kids with Crohn's disease (TISKids): an international multicentre randomised controlled trial. *BMJ Open Gastroenterol*. 2016;3(1):e000123.

For peer review only

22. Levine A, Koletzko S, Turner D et al. The ESPGHAN Revised Porto Criteria for the Diagnosis of Inflammatory Bowel Disease in Children and Adolescents. *J Pediatr Gastroenterol Nutr*. 2013;58(6):795-806.

23. Levine A, Griffiths A, Markowitz J et al. Pediatric modification of the Montreal classification for inflammatory bowel disease. *Inflamm Bowel Dis.* 2011;17(6):1314-1321.

24. Turner D, Griffiths AM, Wilson D et al. Designing clinical trials in paediatric inflammatory bowel diseases: a PIBDnet commentary. *Gut*. Published online first: 12 April 2019 Apr. doi:10.1136/gutjnl-2018-317987

25. Chan A, Tetzlaff J, Altman D et al. SPIRIT 2013 Statement: Defining Standard Protocol Items for Clinical Trials. *Ann Intern Med*. 2013;158(3):200-207.

For peer review only

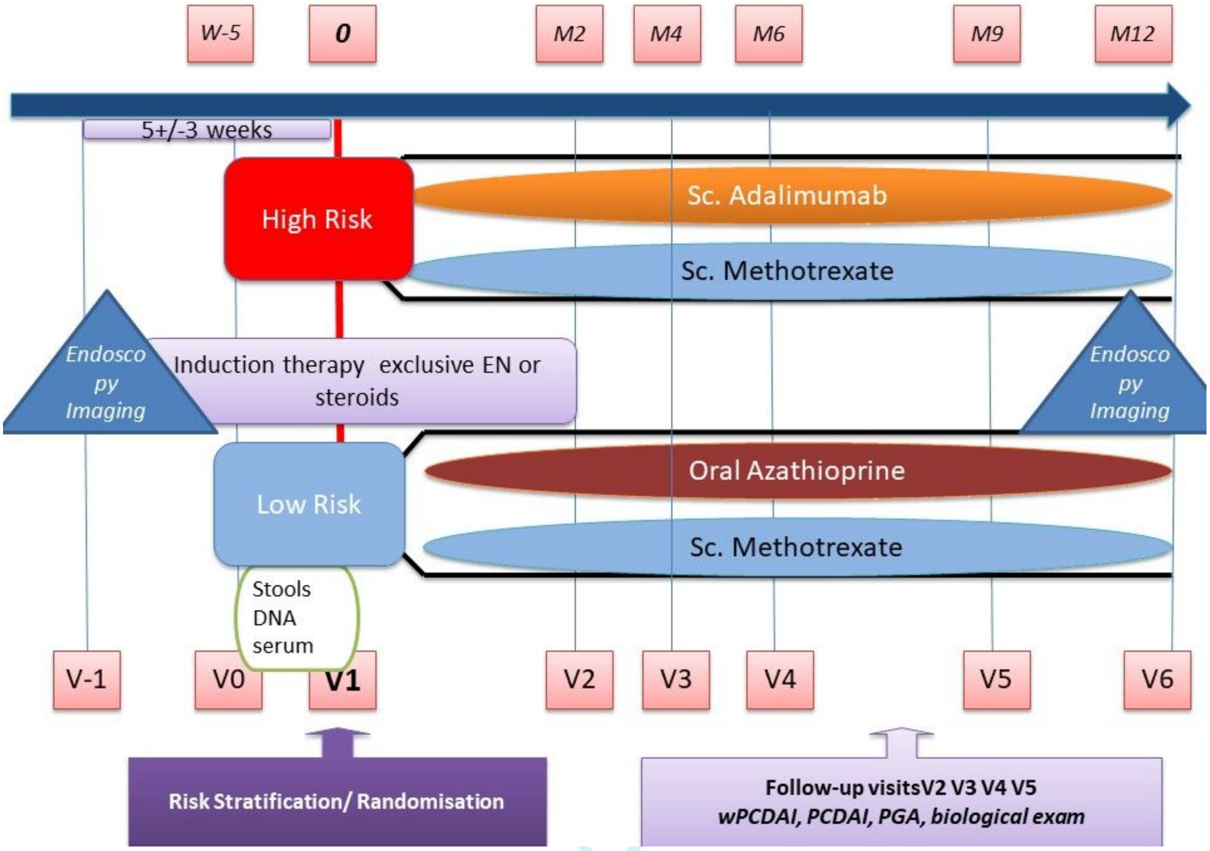

Figure 1 – Study Design of the REDUCE-RISK in CD trial

For peer review only

Participating Sites – REDUCE-RISK in CD Study		
**Country**	**City**	**Site**
**Belgium**	Brussels	Universitair Ziekenhuis
	Brussels	Clinique Saint Luc UCL
	Liège	Clinique de l'Espérance
	Brussels	HUDERF
**Canada**	Toronto	SickKids
**Czech Republic**	Prague	FN Motol
	Plzeň	FN Plzeň
	Prague	First Medical Faculty
**France**	Paris	Hôpital Necker Enfants Malades
	Paris	Hôpital Robert Debré
	Paris	Hôpital Armand Trousseau
	Le Havre	Hôpital Jacques Monod
	Nancy	Hôpitaux de Brabois
	Toulouse	Hôpital des Enfants
	Tours	Hôpital Clocheville
	Caen	CHU Caen Côte de Nacre
	Marseille	Hôpital de la Timone
**Germany**	Munich	Childrens Hospital
	Ulm	Universitätsklinikum
	Hannover	MHH Kinderklinik
	Giessen	UKGM
	Berlin	Charite Hospital
**Greece**	Athens	Children's Hospital "AGIA SOFIA"
**Israel**	Jerusalem	Shaare Zedek Medical Center
	Tel Aviv	Wolfson Medical Center
	Petah Tikva	Schneider Children's Medical Center
	Ramat Gan	Sheba Medical Center
	Haifa	Rambam Medical Center
**Italy**	Rome	Università degli Studi di Roma La Sapienza
	Bologna	Maggiore Hospital
	Florence	Azienda Ospedaliero Universitaria
	Parma	Azienda Ospedaliero
	Rome	Opsedale Pediatrico Bambino Gesù
**Netherlands**	Rotterdam	Erasmus Medical Center
**Poland**	Warsaw	Centrum Zdrowia MDM
	Białystok	Uniwersytecki Dziecięcy Szpital Kliniczny
	Łódź	Instytut Centrum Zdrowia Matki Polki
**United Kingdom**	Glasgow	The Royal Hospital for Children
	London	Royal London Children's Hospital, Barts Health NHS Trust
	Edinburgh	Sick Children's Hospital
	Birmingham	Children's Hospital
	Oxford	John Radcliffe Hospital

Supplementary Table 1 – Sites participating in REDUCE-RISK in CD

For peer review only

PIBD-SETQuality

European Commission

INFORMED CONSENT FORM

Parents/Guardian Informed Consent Form for Participation of a Minor in a Clinical Trial

Risk-stratified randomized controlled trial in paediatric Crohn's Disease: Methotrexate versus azathioprine or adalimumab for maintaining remission in patients at low or at high risk for aggressive disease course, respectively – a treatment strategy.

Dear parents,

Your child's doctor, Dr………………………., working at ………………………………….. Hospital, propose your child to participate in a clinical trial related to its disease.

It is important to read this note carefully before taking any decision. Do not hesitate to ask the physician all the questions you may have about it.

The participation of your child is based on volunteering. Therefore, your child can refuse to participate or stop its participation in the trial at any time, all of this without prejudice to the patient's right to receive the standard treatment.

If you refuse your child to participate, he/she will still receive the best medical support.

Purpose of the research and trial's objectives

Your child has just been diagnosed with Crohn's disease. The disease is caracterised by chronic inflammation of the digestive track (bowel/colon). This disease changes over between remission period and relapse period. There are efficient drugs able to prevent relapse and to maintain remission. In order to reduce the likelihood of long-term complications, induction treatment has already been prescribed to your child. This first treatment has to be followed up by a maintenance treatment that will be introduced to avoid the inflammation from returning. Consensus guidelines of ECCO / ESPGHAN (french and european IBD specialized organizations) recommend 3 efficient treatments: either immunosuppressive treatment with Thiopurines (azathioprine and 6-mercaptopurine), or Methotrexate or anti TNF (adalimumab).

So far, no clinical trial has been conducted to compare those 3 treatments in children with Crohn's disease and to answer the following question: "Which treatment is the most efficient, for which patient and/or in which situation?"

Progression of Crohn's disease is not the same for all patients. That's why this study will first classified all children in high and low risk groups based on more or less severe course of Crohn's disease. The lower risk group will be randomized (which is like tossing a coin) to receive either thiopurines or methotrexate as maintenance treatment. The high risk group will be randomized to receive either methotrexate or adalimumab. Results will show whether there is different efficiency between the 3 drugs for patients with a more or less severe disease.

Sponsor

PIBD-Net (www.pibd-net.org) is a global, international and non-profit organisation gathering physicians and researchers specialised in inflammatory bowel diseases. The acronym stands for Pediatric Inflammatory Bowel Diseases Network and it is present in 31 countries (Europe, North America, Australia and Japan). This organisation is dedicated in improving the medical care of children with inflammatory bowel disease through the establishment of clinical researches.

PIBD-Net and partners received funding from European Commission for Horizon 2020 program (project no.668023) in order to perform this research.

The approximate number of participants and duration of follow-up

A total of 312 new-onset children with Crohn's disease (136 in that high-risk group and 176 in the low risk) will be enrolled in many sites around the world. The period of recruitment is 45 months and your child will be followed up for 12 months after enrolment.

For peer review only - http://bmjopen.bmj.com/site/about/guidelines.xhtml

PIBD-SETQuality

European Commission

What will happen to your child during the trial?

If you agree to have your child participating in this study, your child will be first directed into one of two groups based on certain predictors of its disease (such as its location and severity). You will know which group your child is in. Next, your child will be randomized to receive maintenance treatment namely:

- **METHOTREXATE or AZATHIOPRINE for the low risk group** ;
- **METHOTREXATE orADALIMUMABfor the high risk group** ;

You cannot choose the treatment group but you will know which drug your child will receive.

<u>You will not be asked to come to clinic just because of this study which is designed to mirror regular follow-up in clinic.</u> After signing the informed consent allowing your child to be part of this research, your child will have clinic visit every 2 months during the first 6 months and then every 3 months during the last 6 months. At each visit, a clinic examination is performed and blood, urine and stool samples are collected (this process follows our standard clinical practices of our patients not involved in this protocole).

Your doctor, your child and yourself will be asked to complete short questionnaires to evaluate the quality of life of your child. <u>Most of the recorded data for this study is needed anyway as part of a regular visit</u> but there might be a few more questions we will ask you for this study.

We will also contact you over the phone at week 4 to ask how your child feels regarding its Crohn's disease, whether your child has any bad reactions to the medications your child will receive and check your child compliance to the treatment.

To optimize the medications we prescribe, we will draw 12 ml (3-4 teaspoons) of blood at inclusion visit, 10ml at visit V2, and then 5ml at each next study visits to measure the level of the medications your child will receive. A urine sample will be collected at inclusion visit and a DNA sample (either 5ml of blood or buccal swab) will be collected at the beginning of the study, and also in case of drug intolerance.

We will also collect your child stool (poop) six times during the year to measure the amount of inflammation in its bowel as well as bacteria flora in its intestine.

We will evaluate whether the drugs work based on

- completed questionnaires
- clinic examination
- results of biological samples

Optional Ancillary study (« ADA STEP-up »)

In case of failing (intolerance or relapse) of your child immunomodulator therapy (: either azathioprine/6MP or methotrexate), your child will be invitated to participate in the ancillary study. If you agree, your child will be prescribed adalimumab during 12 months.

This adalimumab treatment can increase the study duration by a maximum of 9 months, meaning a maximum of 3 additional visits. Those visits are identical to the regular follow up study visits.

The expected benefits to the participant or to others because of the trial

The medications your child will receive in this study are not experimental and thus there are no direct benefit for using these drugs that are available outside of the study. However, your child will be monitored closely to ensure optimization of the treatment by adapting drug amounts based on new analyses (urine, DNA, blood and stool samples). In addition, patients involved in this study have access to molecular analyses in order to better understand why a patient is less responsive than expected. That might result in a more tight control of the disease and better monitoring of the treatment of your child.

For peer review only

For peer review only - http://bmjopen.bmj.com/site/about/guidelines.xhtml

PIBD-SETQuality

European Commission

After study completion, we will have the required data to recommend how to use these medications in new children who develop Crohn's disease.

Risks added by the research

As previously mentionned, all medications used in this trial are not experimental and are being used very often in clinical practice in children/adolescents and adults with Crohn's disease. There is no additional risk compares to regular clinical practices. The known risks and discomfort that may be anticipated are listed below.

Known risks and discomfort that may be anticipated
The medications yourchild will receive in this study are not experimental but used in regular practices. They can also be associated with side effects (all described in the corresponding drug information sheet).

- ✓ **METHOTREXATE: weekly subcutaneous (under the skin)** This drug may be associated with side effects mainly in the day of the injection including flu-like symptoms, nausea, vomiting, headache or fatigue. Your child will be asked to take a vitamin called folic acid which will reduce these non-dangerous side effects. This injection may cause slight discomfort.METHOTREXATE may cause reduced blood counts, especially white blood cells and elevated liver enzymes. Thoses parameters will be checked regularly. In this study, molecular analyses will be performed to screen patients who can't tolerate METHOTREXATE..This drug causes an unusual sensitiveness to the sun (however, there is no data stating that it increases the risk of cancer or lymphoma). METHOTREXATE can cause foetal abdormalities so pregnancy is not allowed and efficient contraceptive is essential (for both male and female).

- ✓ **AZATHIOPRINE (or 6MP): to be taken orally.** THIOPURINES may cause reduced blood counts, especially white blood cells and elevated liver enzymes. Thoses parameters will be checked regurlarlyIn this study, molecular analyses will be done in order to screen patients who can't tolerate those drugs. In some rare cases (<3%), the drug may cause inflammation in the pancreas which is usually mild and not dangerous. As all drugs, THIOPURINES can't be tolerated by some patients due to allergy. Approximately 10% of children will not tolerate the drug because of nausea, vomiting, tummy pain, diarrhea, headaches or fever..THIOPURINES are associated with an increased infectious risk (about 1%). Infections are likely cause by viruses. In rare cases, THIOPURINES may increase risk for blood cancer called lymphoma (especially for patients > 65 year old). This drug causes an unusual sensitiveness to the sun and can be associated with skin cancer in case of significant sun exposure.

ADALIMUMAB: subcutaneous (under the skin) injections every 2 weeks Adalimumab is associated with minor pain during the injection and local reactions could appear with minimal significance. ADALIMUMAB is associated with an increased infectious risk. However, serious infections are uncommon. Before starting ADALIMUMAB treatment, tuberculosis must be excluded. With time, the effect of adalimumab may wain as a result of the development of antibodies against the drug. Skin inflammatory damages (such as « psoriasis ») were observed in some patients. Anti TNF drugs have been closely monitored since their use as standard treatment. Those drugs may be responsible of heart failure for patients with severe heart disease, hepatitis, decreasing blood cells, demyelinating neurologic disease, or lupus (without affecting main organs). In addition, some cases of cancer have been notified in patient treated by ADALIMUMAB, but risks of cancer is slightly increased only for melanoma. Number of cancer seems not to be increased compared to patients with Crohn's diseases and without being treated with those drugs.

Circumstances under which participation in the medical trial may be discontinued in accordance with the decision of the investigator or the Sponsor:

a. The doctor has the right to take your child out of the study at any time. This will be made after clinical considerations, your child's side effects from the drugs, intolerance to the drugs or lose of response.

b. Regulatory authorities (Ministry of Health or Ethics committee), may stop your child participating in the study.

For peer review only - http://bmjopen.bmj.com/site/about/guidelines.xhtml

PIBD-SETQuality

European Commission

An explanation of alternative treatments, their advantages and disadvantages, if any, for the participant:

The current standard therapy for maintenance therapy in Crohn's disease is either METHOTREXATE or thiopurines or anti-TNF biologics for the more severe Crohn diseaes. This is exactly the medications given also as part of this study. The difference is that instead letting you and the doctor choose between the options, the choice is standardized based on predictive variables of your disease and randomization. If you choose not to have your child participating in the study, your child will likely receive anyway one or more of these three drugs. The only exception would be that if anti-TNF is prescribed, either adalimumab or infliximab can be given and as part of this study your child will receive adalimumab only. However, your child will not have access to molecular analyses described in this protocole with a close monitoring of drug safety. Indeed, those 'new" analyses are not done in the standardclinical practise of Crohn's disease.

If you participate in this study, what will you have to do more than usual ?

If you agree to have your child participating in this study, please make sure to follow the listed points belowPlease come at your appointements with your child. If not possible, please inform its physician as soon as possible

- Please ensure that your child takes the treatment as instructed by its doctor
- Please inform the physician involved in the study of any event happening during the research (such as hospitalization,…)
- Your child must not participate in any other clinical trial that involves the use of an investigational product throughout the course of this trial. It is to avoid accidents such as possible interactions between medicines.

Biological samples collected during this research project

If you agree to have your child participating in this research, additional blood, urine and stool samples will be collected at the same time as our standard clinic samplings. Please see below:

✓ 10ml of blood during inclusion visit (on randomisation day).
✓ 5ml of blood (PAX tube) during inclusion visit for RNA analyses
✓ 10ml of blood at follow up visits M2 (2 months after inclusion)
✓ 5 ml of blood at follow up visits M4, M6, M9 and M12 (4, 6, 9, 12 months after inclusion)
✓ Stool sample at inclusion visit and follow up visits M2, M4, M6, M9 and M12 (2, 4, 6, 9, 12 months after inclusion visit).
✓ A DNA sample will be collected at inclusion visit and in case of intolerance of one of the drugs for DNA analyses.
✓
✓ Urine sample (15ml) will be collected at M2 visit (2 months after inclusion).

Those samples will be sent to specialized laboratories in order to be used to perform specific studies such as adalimumab, methotrexate, thiopurine analyses and serology, genetic (both DNA and RA), microbiology studies. They will also be re-used for further testing on Crohn's disease, its diagnosis and its treatment as well as efficacy and tolerance by molecular ("omic") analyses.

At any time, you can request to your clinician to have those biological samples destroyed or not to be used for further researches.

Confidentiality

As part of biomedical research in which PIBD-Net sponsor proposes your child's participation, treatment of personal data will be set up to analyse results of this research based on its aim. Therefore, your child medical data and quality of life will be transferred to PIBD-Net sponsor. Those data will be anonymous and identified by a coded number and its initials. Those confidential data could be transferred to local and foreign authorities. If your child has to be withdrawn for any reasons, collected data prior its withdrawal will be used unless you do not want them to.

For peer review only

For peer review only - http://bmjopen.bmj.com/site/about/guidelines.xhtml

PIBD-SETQuality

European Commission

Then, you will have to inform the physician accordingly.

According to the EU General Data Protection Regulation (GDPR) dated on 26May2018, you have the right to access to your child and your personal data, modify them and oppose the use of your child and yourdata. You have also the right to request that your child and your personal data are erased, are limited in use, and to ask for a complete copy of all data collected from you and your child for the study. You can contact the Data Privacy Officer (DPO) of the sponsor at any time at dpo@pibd-net.org for any request regarding your child and your personal data.

Data collected for the study are transferred outside of the EU, as our database is based in Israel. However, we guarantee that data protection will be as strict as requested by GDPR.

Voluntary participation

Your participation in this research is entirely voluntary. It is your choice whether to have your child participating or not, all the services your child receives at this hospital will continue and nothing will change. If you choose not to participate in this research project, your child will be offered the treatment that is routinely offered in this hospital for Crohn’s disease. You may change your mind later and stop participating even if you agreed earlier.

Right to refuse or withdraw

Your child does not have to take part in this research if you do not wish to do so and refusing to participate will not affect its treatment in any way. Your child will still have all the benefits that it would otherwise have at this hospital. You may stop participating in the research at any time that you wish without losing any of its rights as a patient here. Its treatment at this hospital will not be affected in any way.

Alternatives to participating

If you do not wish that your child takes part in the research, your child will be provided with the established standard treatment available at this hospital.

Reimbursement

There is no reimbursement for participating in this study. There are no special visits to the hospital excepted during this study. All DNA, blood, urine and stool samples will be taken at the time of a routine clinic visit.

This proposal has been reviewed and approved by [name of the local IRB], which is a committee whose task it is to make sure that research participants are protected from harm.

For peer review only

For peer review only - http://bmjopen.bmj.com/site/about/guidelines.xhtml

PIBD-SETQuality

Informed consent form

We, the undersigned :

M, Miss, *(name, first name of parent/legal guardian)* ……………………………………………………………..

M, Miss, *(name, first name of parent/legal guardian)* ……………………………………………………………..

I, M, Miss, …………………………………………………………………..*(name, first name of parent)* hereby declare that my consent below has been given voluntarily and that I have understood all of the above. I undertake to also inform the child's father/mother *(please cross off as appropriate)* of my consent for the participation of our child in the clinical trial. If the child's father/mother *(please cross off as appropriate)* does not agree to affix his/her consent to mine, I undertake to inform the physician in-charge and to withdraw my consent for the participation of my child in the clinical trial. I have also received a lawfully and dated copy of this informed consent form

I agree that my child (name, first name of the child)……………………………………………………**takes part of the study named "***Risk-stratified randomized controlled trial in paediatric Crohn's Disease : Methotrexate versus azathioprine or adalimumab for maintaining remission in patients at low or at high risk for aggressive disease course, respectively – a treatment strategy* " , managed by PIBD Net. It has been explained to us by *(name, first name of explaining investigator /sub investigator, phone,)*…………………………………………
……
……………………………………………………………………………………physician in this clinical trial.

- We hereby declare that we agree for our child to participate in the clinical trial as detailed in this document
- Our child has been informed and agreed to take part of this clinical trial.
- We had the opportunity to ask all the questions we had to the physician who explained potential risks and constraints linked to our child participation in this clinical trial.
- We received appropriate answers to all our questions
- We hereby declare that at the time of signing this document, our child is not participating in another clinical trial that involves the use of any investigational product, and that we undertake that our child will not participate in any other clinical that involves the use of an investigational product throughout the course of this trial.
- We declare that our child has a health insurance.
- .We hereby declare that we are free to choose that our child will not participate in the clinical trial, and that we are free to stop our child participation in the trial at any time, and all of this without prejudice to our child's right to receive the standard treatment. Then, we will inform the physician whether data collected prior our decision can be used or not.
- We have been informed that the doctor has the right to take our child out of the study at any time, if needed.
- That in case of completing a questionnaire – we are entitled not to answer all or some of the questions in the questionnaire.
- We are informed that samples collected during this clinical trial will be kept and used for further testing on Crohn's disease. We can decide at any time not to have those samples used by informing our child physician.
- That we are guaranteed confidentiality concerning the identity of the patient and that of the parents/guardians. This confidentiality will be kept by all those concerned with and involved in the clinical trial, and their identity will not be disclosed in any publication.
- That the Medical Institution has arranged for appropriate insurance coverage of the investigators, physicians and medical staff involved in the clinical trial, against claims filed by clinical trial participants and/or third party claims related to the clinical trial, either during the course of the trial or thereafter. This is without prejudice to our rights under the law.
- That in case of pregnancy during the course of the clinical trial, the girl/woman will be counselled (by the principal investigator) concerning the possible effects on the foetus and the fate of the pregnancy, including

For peer review only

For peer review only - http://bmjopen.bmj.com/site/about/guidelines.xhtml

PIBD-SETQuality

European Commission

the possibility of discontinuing the pregnancy.

- We hereby declare that our below consent has been given voluntarily and that we have understood all of the above mentioned. We also received a lawfully signed and dated copy of this informed consent.
- By signing this consent form, we authorize the sponsor of the clinical trial, the Institutional Helsinki Committee, the auditing entity at the Medical Institute and the Ministry of Health direct access to the patient's medical file, to verify the clinical trial methods and the clinical data. This access to our child medical information will be performed with confidentiality maintained, according to the laws and procedures of maintaining confidentiality.
- We declare that we are informed and give our approval to receive all information related to our child participation in this clinical trial. We know that data will only be used for treatment and follow up cares
- We hereby declare that we know and agree to have the information on our child's participation in the clinical trial provided to his/her attending physician at the HMO/Health care Services with which our child is insured, in case the clinical trial involved the provision of services : performing medical examinations or supplying devices or products or implants. We know that the HMO will not use this information for purposes other than medical treatment and follow up

I agree to have my child participating in the ancillary study (« ADA STEP-up »)

Yes ☐ No ☐ *[please tick]*

**Signature of parents or guardians/representatives of the patient**	**Signature of the child**
Name, First Name :______________________<br>Date : Signature :<br><br>Name, First Name :______________________<br>Date : Signature :	Name, First Name :______________________<br>Date : Signature :

Declaration of the Investigator/Sub-Investigator :This consent was obtained by me after I have explained all the above mentioned to the parents (or guardians) of the clinical trial participant and ensure that all my explanations were understood by them.

**Investigator/Sub-investigator' Signature :**
Name, First Name: ______________________________
Date: Signature :

This is a triplicate document. First / original copy to be kept by the investigator for 15 years, second copy to be given to parents or legal guardians, third copy to be kept in Investigator files (under sealed envelope).

For peer review only - http://bmjopen.bmj.com/site/about/guidelines.xhtml
For peer review only

PIBD-SETQuality

European Commission

Informed consent for Genetic Analyses

Hereby declare that we agree for genetic examinations of our child to study genes involved in tolerance / non tolerance of the drugs by molecular ("omic") analyses and analyses of drug efficacy in Crohn disease's patients.

Hereby declare that we agree that all recorded data collected during this trial including genetic data can be processed by the sponsor or acting as sponsor. I understand that, as stipulated in the General Data Protection Regulation, I can access, modify, erase or ask for a copy of my child's personal data and my personal data at any time, by asking to the investigator who will contact the sponsor.

We can decide not to participate anymore in the genetic part of the trial by informing our doctor who will inform the sponsor.

Yes ☐ No ☐ *[please tick]*

Hereby declare that we agree that all biological samples collected during this trial can be used for future genetic research on Crohn's disease.

Yes ☐ No ☐ *[please tick]*

Parents/guardians Signature:

Name, First name:

Date : Signature :

Name, First name:

Date : Signature :

Investigator Signature:

Name, First name:

Date : Signature :

For peer review only

For peer review only - http://bmjopen.bmj.com/site/about/guidelines.xhtml

Reporting checklist for protocol of a clinical trial.

Based on the SPIRIT guidelines.

Instructions to authors

Complete this checklist by entering the page numbers from your manuscript where readers will find each of the items listed below.

Your article may not currently address all the items on the checklist. Please modify your text to include the missing information. If you are certain that an item does not apply, please write "n/a" and provide a short explanation.

Upload your completed checklist as an extra file when you submit to a journal.

In your methods section, say that you used the SPIRITreporting guidelines, and cite them as:

Chan A-W, Tetzlaff JM, Altman DG, Laupacis A, Gøtzsche PC, Krleža-Jerić K, Hróbjartsson A, Mann H, Dickersin K, Berlin J, Doré C, Parulekar W, Summerskill W, Groves T, Schulz K, Sox H, Rockhold FW, Rennie D, Moher D. SPIRIT 2013 Statement: Defining standard protocol items for clinical trials. Ann Intern Med. 2013;158(3):200-207

		Reporting Item	Page Number
**Administrative information**			
Title	#1	Descriptive title identifying the study design, population, interventions, and, if applicable, trial acronym	1
Trial registration	#2a	Trial identifier and registry name. If not yet registered, name of intended registry	4
Trial registration: data set	#2b	All items from the World Health Organization Trial Registration Data Set	Throughout manuscript
Protocol version	#3	Date and version identifier	21
Funding	#4	Sources and types of financial, material, and other support	30
Roles and responsibilities:	#5a	Names, affiliations, and roles of protocol contributors	1,2,30

For peer review only

contributorship			
Roles and responsibilities: sponsor contact information	#5b	Name and contact information for the trial sponsor	29
Roles and responsibilities: sponsor and funder	#5c	Role of study sponsor and funders, if any, in study design; collection, management, analysis, and interpretation of data; writing of the report; and the decision to submit the report for publication, including whether they will have ultimate authority over any of these activities	30
Roles and responsibilities: committees	#5d	Composition, roles, and responsibilities of the coordinating centre, steering committee, endpoint adjudication committee, data management team, and other individuals or groups overseeing the trial, if applicable (see Item 21a for data monitoring committee)	23,30
**Introduction**			
Background and rationale	#6a	Description of research question and justification for undertaking the trial, including summary of relevant studies (published and unpublished) examining benefits and harms for each intervention	6-8
Background and rationale: choice of comparators	#6b	Explanation for choice of comparators	6-7
Objectives	#7	Specific objectives or hypotheses	8-10
Trial design	#8	Description of trial design including type of trial (eg, parallel group, crossover, factorial, single group), allocation ratio, and framework (eg, superiority, equivalence, non-inferiority, exploratory)	
**Methods: Participants, interventions, and outcomes**			

For peer review only

Study setting	#9	Description of study settings (eg, community clinic, academic hospital) and list of countries where data will be collected. Reference to where list of study sites can be obtained	Supplemental table 1
Eligibility criteria	#10	Inclusion and exclusion criteria for participants. If applicable, eligibility criteria for study centres and individuals who will perform the interventions (eg, surgeons, psychotherapists)	10-12
Interventions: description	#11a	Interventions for each group with sufficient detail to allow replication, including how and when they will be administered	15-16
Interventions: modifications	#11b	Criteria for discontinuing or modifying allocated interventions for a given trial participant (eg, drug dose change in response to harms, participant request, or improving / worsening disease)	18, 19,22
Interventions: adherance	#11c	Strategies to improve adherence to intervention protocols, and any procedures for monitoring adherence (eg, drug tablet return; laboratory tests)	19-21
Interventions: concomitant care	#11d	Relevant concomitant care and interventions that are permitted or prohibited during the trial	13,23
Outcomes	#12	Primary, secondary, and other outcomes, including the specific measurement variable (eg, systolic blood pressure), analysis metric (eg, change from baseline, final value, time to event), method of aggregation (eg, median, proportion), and time point for each outcome. Explanation of the clinical relevance of chosen efficacy and harm outcomes is strongly recommended	9-10
Participant timeline	#13	Time schedule of enrolment, interventions (including any run-ins and washouts), assessments, and visits for participants. A schematic diagram is highly recommended (see Figure)	See figure 1<br>8,12,15,16-19
Sample size	#14	Estimated number of participants needed to achieve study objectives and how it was determined, including clinical and statistical assumptions	27

For peer review only

		supporting any sample size calculations	
Recruitment	#15	Strategies for achieving adequate participant enrolment to reach target sample size	Not listed
**Methods: Assignment of interventions (for controlled trials)**			
Allocation: sequence generation	#16a	Method of generating the allocation sequence (eg, computer-generated random numbers), and list of any factors for stratification. To reduce predictability of a random sequence, details of any planned restriction (eg, blocking) should be provided in a separate document that is unavailable to those who enrol participants or assign interventions	15,16,22
Allocation concealment mechanism	#16b	Mechanism of implementing the allocation sequence (eg, central telephone; sequentially numbered, opaque, sealed envelopes), describing any steps to conceal the sequence until interventions are assigned	22
Allocation: implementation	#16c	Who will generate the allocation sequence, who will enrol participants, and who will assign participants to interventions	15
Blinding (masking)	#17a	Who will be blinded after assignment to interventions (eg, trial participants, care providers, outcome assessors, data analysts), and how	22
Blinding (masking): emergency unblinding	#17b	If blinded, circumstances under which unblinding is permissible, and procedure for revealing a participant's allocated intervention during the trial	N/A
**Methods: Data collection, management, and analysis**			
Data collection plan	#18a	Plans for assessment and collection of outcome, baseline, and other trial data, including any related processes to promote data quality (eg, duplicate	24-25

For peer review only

		measurements, training of assessors) and a description of study instruments (eg, questionnaires, laboratory tests) along with their reliability and validity, if known. Reference to where data collection forms can be found, if not in the protocol	
Data collection plan: retention	#18b	Plans to promote participant retention and complete follow-up, including list of any outcome data to be collected for participants who discontinue or deviate from intervention protocols	Not listed
Data management	#19	Plans for data entry, coding, security, and storage, including any related processes to promote data quality (eg, double data entry; range checks for data values). Reference to where details of data management procedures can be found, if not in the protocol	25
Statistics: outcomes	#20a	Statistical methods for analysing primary and secondary outcomes. Reference to where other details of the statistical analysis plan can be found, if not in the protocol	25-26
Statistics: additional analyses	#20b	Methods for any additional analyses (eg, subgroup and adjusted analyses)	26-27
Statistics: analysis population and missing data	#20c	Definition of analysis population relating to protocol non-adherence (eg, as randomised analysis), and any statistical methods to handle missing data (eg, multiple imputation)	26
**Methods: Monitoring**			
Data monitoring: formal committee	#21a	Composition of data monitoring committee (DMC); summary of its role and reporting structure; statement of whether it is independent from the sponsor and competing interests; and reference to where further details about its charter can be found, if not in the protocol. Alternatively, an explanation of why a DMC is not needed	23
Data monitoring:	#21b	Description of any interim analyses and stopping	23

For peer review only

interim analysis		guidelines, including who will have access to these interim results and make the final decision to terminate the trial	
Harms	#22	Plans for collecting, assessing, reporting, and managing solicited and spontaneously reported adverse events and other unintended effects of trial interventions or trial conduct	23
Auditing	#23	Frequency and procedures for auditing trial conduct, if any, and whether the process will be independent from investigators and the sponsor	24
**Ethics and dissemination**			
Research ethics approval	#24	Plans for seeking research ethics committee / institutional review board (REC / IRB) approval	4
Protocol amendments	#25	Plans for communicating important protocol modifications (eg, changes to eligibility criteria, outcomes, analyses) to relevant parties (eg, investigators, REC / IRBs, trial participants, trial registries, journals, regulators)	22
Consent or assent	#26a	Who will obtain informed consent or assent from potential trial participants or authorised surrogates, and how (see Item 32)	29-30
Consent or assent: ancillary studies	#26b	Additional consent provisions for collection and use of participant data and biological specimens in ancillary studies, if applicable	No additional consent see page 30
Confidentiality	#27	How personal information about potential and enrolled participants will be collected, shared, and maintained in order to protect confidentiality before, during, and after the trial	24
Declaration of interests	#28	Financial and other competing interests for principal investigators for the overall trial and each study site	30
Data access	#29	Statement of who will have access to the final trial dataset, and disclosure of contractual agreements that limit such access for investigators	Not provided

For peer review only

Ancillary and post trial care	#30	Provisions, if any, for ancillary and post-trial care, and for compensation to those who suffer harm from trial participation	N/A
Dissemination policy: trial results	#31a	Plans for investigators and sponsor to communicate trial results to participants, healthcare professionals, the public, and other relevant groups (eg, via publication, reporting in results databases, or other data sharing arrangements), including any publication restrictions	29
Dissemination policy: authorship	#31b	Authorship eligibility guidelines and any intended use of professional writers	29
Dissemination policy: reproducible research	#31c	Plans, if any, for granting public access to the full protocol, participant-level dataset, and statistical code	Not planned
**Appendices**			
Informed consent materials	#32	Model consent form and other related documentation given to participants and authorised surrogates	Appendix 1
Biological specimens	#33	Plans for collection, laboratory evaluation, and storage of biological specimens for genetic or molecular analysis in the current trial and for future use in ancillary studies, if applicable	Not provided

None The SPIRIT checklist is distributed under the terms of the Creative Commons Attribution License CC-BY-ND 3.0. This checklist can be completed online using https://www.goodreports.org/, a tool made by the EQUATOR Network in collaboration with Penelope.ai

For peer review only

BMJ Open

A MULTI-NATIONAL RISK-STRATIFIED RANDOMISED CONTROLLED TRIAL IN PAEDIATRIC CROHN'S DISEASE : METHOTREXATE VERSUS AZATHIOPRINE OR ADALIMUMAB FOR MAINTAINING REMISSION IN PATIENTS AT LOW OR HIGH RISK FOR AGGRESSIVE DISEASE COURSE, RESPECTIVELY – A TREATMENT STRATEGY PROTOCOL FOR: THE REDUCE-RISK IN CD PIBD TRIAL

Journal:	*BMJ Open*
Manuscript ID	bmjopen-2019-034892.R1
Article Type:	Protocol
Date Submitted by the Author:	25-Feb-2020
Complete List of Authors:	Harris, Rachel; Royal Hospital for Children Glasgow<br>Aloi, Marina; Sapienza University of Rome, Paediatric Gastroenterology and Liver Unit<br>de Ridder, Lissy; Erasmus MC / Sophia Childrens Hospital, Paediatrics<br>Croft, Nicholas; Barts and The London School of Medicine and Dentistry<br>Koletzko, Sibylle; Dr. v. Hauner Children's Hospital, Pediatric Gastroenterology and Hepatology; Collegium Medicum University of Warmia and Mazury, Department of Pediatrics<br>Levine, Arie; Tel Aviv University, Edith Wolfson Medical Center,<br>Turner, Dan; Hebrew University of Jerusalem<br>Veereman, Gigi; UZBrussels-VUB, Pediatric GI; Free University Brussels, University Hospital, Veereman<br>Neyt , Mattias ; ME-TA Medical Evaluation and Technology Assessment<br>Bigot, Laetitia; Hôpital universitaire Necker-Enfants malades, PIBD-Net<br>Ruemmele, Frank; Hôpital universitaire Necker-Enfants malades, Service de Gastroentérologie pédiatrique; Université Paris Descartes<br>russell, richard; Royal Hospital for Children Glasgow, ; RHC Glasgow
**Primary Subject Heading**:	Gastroenterology and hepatology
Secondary Subject Heading:	Paediatrics, Pharmacology and therapeutics, Patient-centred medicine
Keywords:	Inflammatory bowel disease < GASTROENTEROLOGY, Paediatric gastroenterology < GASTROENTEROLOGY, Clinical trials < THERAPEUTICS

SCHOLARONE™
Manuscripts

I, the Submitting Author has the right to grant and does grant on behalf of all authors of the Work (as defined in the below author licence), an exclusive licence and/or a non-exclusive licence for contributions from authors who are: i) UK Crown employees; ii) where BMJ has agreed a CC-BY licence shall apply, and/or iii) in accordance with the terms applicable for US Federal Government officers or employees acting as part of their official duties; on a worldwide, perpetual, irrevocable, royalty-free basis to BMJ Publishing Group Ltd ("BMJ") its licensees and where the relevant Journal is co-owned by BMJ to the co-owners of the Journal, to publish the Work in this journal and any other BMJ products and to exploit all rights, as set out in our licence.

The Submitting Author accepts and understands that any supply made under these terms is made by BMJ to the Submitting Author unless you are acting as an employee on behalf of your employer or a postgraduate student of an affiliated institution which is paying any applicable article publishing charge ("APC") for Open Access articles. Where the Submitting Author wishes to make the Work available on an Open Access basis (and intends to pay the relevant APC), the terms of reuse of such Open Access shall be governed by a Creative Commons licence – details of these licences and which Creative Commons licence will apply to this Work are set out in our licence referred to above.

Other than as permitted in any relevant BMJ Author's Self Archiving Policies, I confirm this Work has not been accepted for publication elsewhere, is not being considered for publication elsewhere and does not duplicate material already published. I confirm all authors consent to publication of this Work and authorise the granting of this licence.

For peer review only

A MULTI-NATIONAL RISK-STRATIFIED RANDOMISED CONTROLLED TRIAL IN PAEDIATRIC CROHN'S DISEASE: METHOTREXATE VERSUS AZATHIOPRINE OR ADALIMUMAB FOR MAINTAINING REMISSION IN PATIENTS AT LOW OR HIGH RISK FOR AGGRESSIVE DISEASE COURSE, RESPECTIVELY – A TREATMENT STRATEGY PROTOCOL FOR THE REDUCE-RISK IN CD PIBD TRIAL

Rachel E. Harris[1]; Marina Aloi[2]; Lissy de Ridder[3]; Nicholas M Croft[4]; Sibylle Koletzko[5,6]; Arie Levine[7]; Dan Turner[8]; Gigi Veereman [9]; Mattias Neyt[10] Laetitia Bigot[11]; Frank M. Ruemmele[12,13*]; Richard K. Russell[1*]; on behalf of the PIBD SETQuality consortium and PIBDnet

1. Department of Paediatric Gastroenterology, Hepatology and Nutrition, Royal Hospital for Children, Glasgow, UK
2. Paediatric Gastroenterology and Liver Unit, Department of Paediatrics, Sapienza University of Rome, Rome, Italy
3. Erasmus MC/Sophia Children's Hospital, Rotterdam, The Netherlands
4. Barts and the London School of Medicine, Queen Mary University of London, London, UK.
5. Department of Pediatrics, Dr. von Hauner Children's Hospital, University Hospital, LMU Munich, Munich, Germany
6. Department of Pediatrics, Gastroenterology and Nutrition, School of Medicine Collegium Medicum University of Warmia and Mazury, Olsztyn, Poland
7. Paediatric Gastroenterology and Nutrition Unit, Tel Aviv University, Edith Wolfson Medical Center, Holon, Israel

For peer review only

8. Institute of Paediatric Gastroenterology, Shaare Zedek Medical Center, The Hebrew University of Jerusalem, Jerusalem, Israel

9. Department of Paediatric Gastroenterology and Nutrition, UZ Brussel - Vrije Universiteit Brussel, Brussels, Belgium

10. ME-TA Medical Evaluation and Technology Assessment, Merendree, Belgium

11. Pediatric Inflammatory Bowel Disease network (PIBD-net), Hôpital Necker Enfants Malades, Paris, France

12. Université Paris Descartes, Sorbonne Paris Cité, Paris, France

13. Assistance Publique-Hôpitaux de Paris, Hôpital Necker-Enfants Malades, Service de Gastroentérologie pédiatrique, Paris, France

* FMR and RKR share senior authorship

Corresponding Author:

Professor Richard K. Russell

Department of Paediatric Gastroenterology, Hepatology and Nutrition

Royal Hospital for Children

1345 Govan Road

Glasgow

G51 4TF

UNITED KINGDOM

Telephone: 0044 141 451 6543

Email: richardrussell@nhs.net

For peer review only

Key Words: Paediatric Gastroenterology, Inflammatory Bowel Disease, Crohn's Disease, Immunomodulators

ABSTRACT

Introduction

Immunomodulators such as thiopurines (azathioprine (AZA)/6-mercaptopurine (6MP)), methotrexate (MTX) and biologics such as adalimumab (ADA) are well established for maintenance of remission within paediatric Crohn's disease (CD). It remains unclear however which maintenance medication should be used first-line in specific patient groups.

Aims

To compare the efficacy of maintenance therapies in newly diagnosed CD based upon stratification into high and low risk groups for severe CD evolution; MTX versus AZA/6MP in low-risk and MTX versus ADA in high-risk patients. Primary end point: sustained remission at 12 months (weighted paediatric Crohn's disease activity index ≤12.5 and C-reactive protein ≤1.5-fold upper limit) without relapse or ongoing requirement for EEN/steroids 12 weeks after treatment initiation.

Methods and Analysis

REDUCE-RISK in CD is an international multicentre open-label prospective randomised controlled trial funded by EU within the Horizon2020 framework (grant

For peer review only

number 668023). Eligible patients (aged 6-17 years, new-onset disease receiving steroids or EEN for induction of remission for luminal +/- perianal CD are stratified into low and high-risk groups based upon phenotype and response to induction therapy. Participants are randomised to one of two treatment arms within their risk group: low-risk patients to weekly subcutaneous MTX or daily oral AZA/6MP, and high-risk patients to weekly subcutaneous MTX or fortnightly ADA. Patients are followed up for 12 months at pre-specified intervals. Electronic case report forms are completed prospectively. The study aims to recruit 312 participants (176 low-risk; 136 high-risk).

Ethics and Dissemination

ClinicalTrials.gov Identifier: (NCT02852694), authorisation and approval from local ethics committees have been obtained prior to recruitment. Individual informed consent will be obtained prior to participation in the study. Results will be published in a peer-reviewed journal with open access.

Registration Details

NCT02852694; pre-results.

Strengths and limitations

- This is the 1st international prospective RCT comparing 3 different i medications for maintenance of remission in newly diagnosed CD
- This study may better define the most appropriate first-line immunomodulators based upon a risk stratification protocol. .

For peer review only

- Therapeutic efficacy will be supported by drug levels, pharmacogenomics and microbiome analysis as secondary outcomes.
- Inability to blind participants or treating physicians serves as a limitation to this study.
- Blinding of an alternative clinician to assess disease activity during study visits may prove practically difficult in smaller centres.

For peer review only

INTRODUCTION

Crohn's disease (CD) the most common form of inflammatory bowel disease (IBD) in children is a chronic disorder with the potential to affect the whole gastrointestinal tract. The aim of CD treatment is to control active inflammation and achieve bowel healing. Chronic and uncontrolled CD results in poor outcomes for patients, including reduced quality of life, recurrent hospitalisation and potential need for surgical

intervention.[1] Treatments for CD are categorised into those which induce remission (such as steroids[1,2] or exclusive enteral nutrition (EEN)[1,3] and those which maintain remission. Immunomodulators are a mainstay of maintenance treatment in IBD, with the efficacy of thiopurines (e.g. azathioprine (AZA) and 6-mercaptopurine (6MP))[4,5,6] and methotrexate (MTX)[7,8,9,10] well established. Anti-tumour necrosis factor (anti-TNF) therapies (infliximab[11,12] and adalimumab (ADA)[13,14]) including their biosimilars were used in those patients refractory to "traditional" induction or maintenance treatment. More recently in clinical practice patients deemed as high risk have been treated with a biologic without the need for prior use of an immunomodulator.

Due to a lack of treatment strategy trials within the paediatric IBD (PIBD) population however, it remains unclear which of the aforementioned maintenance therapies should be used first-line in individual patients. Randomised controlled trials comparing the use of MTX with thiopurines for maintenance of remission failed to show a significant difference in efficacy between the two.[15,16, 17] A Cochrane review in adults with quiescent CD highlighted the lack of adequately powered trials necessary in order to determine the efficacy and safety of thiopurines compared to other maintenance therapies[4, 10]. The RISK study (observational, non-randomised study) demonstrated improved clinical and growth-based outcomes at 1 year with anti-TNF monotherapy in comparison with immunomodulators; however further investigation into which specific patients are most likely to benefit from these therapies is still required.[18] There is a clear disparity between North America and Europe in terms of which form of immunosuppression is used initially with both concerns about efficacy and safety lying behind these differences, thus there is an urgent need for a head to head study in children to help objectively inform the primary choice of immunosuppression.

For peer review only - http://bmjopen.bmj.com/site/about/guidelines.xhtml

Stratifying patients by risk for complex or severe CD may allow pre-emptive direction of maintenance strategy and potentially an early reduction in disease burden with subsequent improvement in long-term outcomes. The adult IBD Ahead initiative highlighted young age at diagnosis as a risk factor for severity of CD evolution[19]; all patients diagnosed within paediatric services would therefore be considered 'high risk'. Paediatric consensus guidelines suggest that paediatric CD patients at 'high risk for poor outcome' should receive early therapy optimisation to modify progression of their disease.[1] The guidelines list specific features which may be considered predictive for poor outcome in paediatric CD (see Table 1).[1] Patients deemed at high risk for complex disease or poor outcome may benefit from a 'Top-down' approach as the TISKids (a randomised controlled trial from disease diagnosis) aims to investigate[20].

Therefore the PIBDnet consortium recognised the urgent need to investigate the efficacy and safety of immunomodulators and to investigate whether a top-down approach was superior to a traditional 'step-up' for paediatric patients deemed at high risk for rapidly complicated disease course. REDUCE-RISK in CD is a randomised controlled trial (RCT) which aims to compare the effectiveness of immunomodulators for maintenance of remission in newly diagnosed CD based upon risk stratification specifically, the effectiveness of MTX versus AZA/6MP for maintenance of remission who are low risk for rapidly progressive disease and the effectiveness of MTX versus ADA in a high risk group.

METHODS AND ANALYSIS

Study Design

We designed an international multicentre open-label prospective RCT with 4 treatment arms as shown in Figure 1. Following screening and consent, eligible patients are stratified into low and high-risk groups based upon phenotype and disease response to induction therapy (Table 1). Patients are then randomised to one of two arms within their risk group, with low risk patients receiving either weekly subcutaneous MTX or daily oral AZA/6MP and high-risk patients receiving either weekly subcutaneous MTX or fortnightly subcutaneous ADA.

Study End Points

Patients are followed up for 12 months post randomisation. The primary end point of the study is sustained steroid or EEN-free remission at 12 months, defined as weighted Paediatric Crohn's Disease Activity Index (wPCDAI) ≤12.5 and C-reactive protein (CRP) ≤1.5-fold upper limit without a relapse or need for EEN/steroids since week 12.

Secondary end points include comparison of time to first relapse, remission at 12 weeks, growth, adverse events, health related quality of life and patient reported outcomes between the two treatment arms within each risk group but also between low and high risk MTX treated patients. The study also aimed to evaluate clinical predictors for response, including genomic and serological markers and results of drug monitoring (MTX and ADA concentrations) metabolites (6-thioguanine (6-TG) and 6-methylmercaptopurine (6-MMP) in AZA/6MP) and anti-drug antibodies (ADA)

in relation to adherence, toxicity and response. The ancillary study additionally aimed to evaluate the efficacy of ADA in patients treated from inclusion (Top-down) versus patients switched to ADA due to immunomodulator failure (Step-up). Further outcome measures are detailed in Box 1.

Box 1: Study endpoints

Primary Endpoint

- Sustained steroid/EEN-free remission at month 12, where sustained remission is defined as wPCDAI ≤12.5 and CRP ≤1.5 times the upper limit without a relapse or need for EEN/steroids since week 12.

Secondary Endpoints*:*

Comparing the following within 1) the two treatment arms per risk group; 2) methotrexate treatment between high and low risk groups; and 3) TOP-Down adalimumab (high risk group) versus STEP-Up adalimumab (ancillary study):

- Rate of clinical remission at month 12 (physician global assessment (PGA), wPCDAI, paediatric Crohn's disease activity index (PCDAI))
- Relapse free remission with normal CRP at month 12
- Relapse free remission with normal CRP and faecal calprotectin <300 at month 12
- Remission at week 12
- Time to first relapse after week 12
- Faecal calprotectin values at visits 1, 2, 4 and 6 (respectively at month 0, 2, 6 and 12)
- Dropout rates
- Adverse drug event rate
- Height velocity and z-score at baseline and 52 weeks
- Quality of life as measured by the IMPACT 3 questionnaire completed at each study visit
- Health economic evaluation at all visits (forms EQ-5D-Y proxy 1, EQ-5D-Y and EQ-5D-5L, WPAI:CD Caregiver, School Attendance start of the research and follow up visits)

Eligibility Criteria and Recruitment

Full eligibility criteria for the study are listed in Box 2. Patients are eligible if aged 6-17 years with new-onset (<6 months) treatment naïve luminally active and/or perianal fistulising CD diagnosed as per revised Porto criteria[21] receiving steroids or EEN for induction of remission with wPCDAI >40 or CRP >2 times upper limit of normal at diagnosis. Informed consent from must be obtained prior to participation in the study. Patients are excluded in cases of previous use of IBD related medications, pregnancy or refusal to use contraceptives; disease requiring surgery, contraindications to study medication, exposure to live vaccine within 3 weeks, oral anticoagulant or anti-malarial use, current or previous malignancy, significant infection or significant comorbidity.

The planned start date for the study is 01.2017 with planned end 06/2022.

For peer review only

Box 2: Eligibility criteria

Inclusion Criteria

- Patients aged 6-17 years with new-onset (<6 months) treatment naïve active luminal and/or perianal fistulising Crohn's disease diagnosed using established criteria[21]requiring steroids or EEN for induction of remission
- wPCDAI >40 or CRP >2 times upper limit of normal at diagnosis
- Luminal active Crohn's disease (B1) with or without B2 and/or B3 disease behaviour as per Paris classification [22]
- Signed informed consent

Exclusion Criteria

- wPCDAI <42.5 at diagnosis, except where CRP >2 times upper normal limit
- Lack of induction therapy with steroids or EEN
- Previous therapy with any IBD-related medication other than induction therapy as detailed within this protocol with the exception of 5-aminosalicylic acid (5ASA) preparations
- Pregnancy or refusal to use contraceptives during the study period in pubertal patients unless absolute abstinence is confirmed at each study visit
- Lactating mothers
- Perianal fistulising disease requiring surgical therapy
- Patients homozygous for thiopurine methyltransferase (TPMT) mutations or those with TPMT activity <6 nmol/h/ml erythrocytes or <9nmol 6MTG/g Hb/h, unless they qualify as high-risk patients
- Evidence of un-drained and un-controlled abscess/phlegmon
- Contraindication to any drugs used in the trial (including intolerance/hypersensitivity or allergy to study drugs (thiopurines, methotrexate or adalimumab))
- Current or previous malignancy
- Serious comorbidities (e.g. renal insufficiency, hepatitis, respiratory insufficiency) which may interfere with drug therapy or interpretation of outcome parameters or will make it unlikely that the patient will complete the trial.
- Infection with mycobacterium tuberculosis, hepatitis B or C, human immunodeficiency virus (HIV)
- Moderate to severe heart failure (New York Heart Association class III/IV)
- Oral anticoagulant therapy, anti-malarial therapy
- Live vaccine exposure (including yellow fever) less than 3 weeks prior to inclusion

Screening Visit (Visit 0)

The screening visit allows for assessment of eligibility for inclusion in the study, evaluation of the patient's response to induction therapy if already commenced,

For peer review only - http://bmjopen.bmj.com/site/about/guidelines.xhtml

commencement of induction therapy where not commenced, and acquisition of consent and assent.

Induction Therapy

All enrolled patients receive either corticosteroids or exclusive enteral nutrition (EEN) as induction as determined by the clinical team and the patient/caregiver. For EEN any balanced formula (polymeric or elemental) administered orally or via nasogastric tube is permitted and should be prescribed for 6-8 weeks. Tapering of steroids is at the discretion of the prescribing clinician. Adaptation of induction therapy (e.g. dose increase of steroids or return to EEN) or crossover from one induction therapy to the other is permitted in order to achieve remission, however patients must have discontinued their induction therapy by week 12. If induction therapy is not discontinued by week 12 the patient is considered a treatment failure, with the protocol for this detailed below.

<u>Inclusion Visit and Risk Group Allocation (week 5 +/- 3 weeks; visit 1)</u>

In order to incorporate response to initial induction therapy within the risk stratification criteria, inclusion and risk group allocation is performed at week 5 +/- 3 weeks of induction therapy. Data from the screening visit is reviewed with ineligible patients excluded and patients are then stratified into the high or low risk group (Table 1) based upon the ECCO/ESPGHAN consensus guidelines[1]. Patients with

For peer review only

perianal fistulising disease at diagnosis are auto-allocated to the high-risk group regardless of other factors at inclusion visit. All other patients are allocated to the low risk group. Patients with low thiopurine methyltransferase (TPMT) activity or homozygous mutations are excluded should they be categorised as low risk.

DEFINING HIGH RISK CROHN'S DISEASE PATIENTS	
**ECCO/ESPGHAN CONSENSUS GUIDELINES**	**MODIFIED STUDY CRITERIA**
Severe perianal disease	Complex perianal fistulising disease phenotype
Extensive (pan-enteric) disease; deep colonic ulcers on endoscopy	Panenteric disease phenotype (defined as L3 with L4b as per Paris classification[23] or L3 with deep ulcers in the duodenum, stomach or oesophagus not related to non-steroidal anti-inflammatory medications or Helicobacter pylori)
	Overall cumulative disease extent of >/=60cm
Stricturing and penetrating disease at onset	B2, B3 or B2B3 disease behaviour [20]
Marked growth retardation >-2.5 height Z scores	Severe growth impairment (height z-score <-2 or crossing >/= 2 centiles) likely related to Crohn's disease
Persistent severe disease despite adequate induction therapy	Hypoalbuminemia (<30g/L), elevated CRP (at least 2 times upper limit of normal range), or wPCDAI>12.5 despite at least 3 weeks of optimized induction therapy with steroids or EEN
Severe osteoporosis	Not included

Table 1 – Definition of high-risk patients based upon ECCO/ESPGHAN consensus guidelines[1] (ECCO – European Crohn's and Colitis Organisation; ESPGHAN – European Society for Paediatric Gastroenterology, Hepatology and Nutrition; CRP – C-reactive protein; wPCDAI – weighted Paediatric Crohn's Disease Activity Index; EEN – exclusive enteral nutrition)

For peer review only

Randomisation and Treatment Allocation

Randomisation is undertaken following allocation to high or low risk group at week 5 +/- 3 weeks. This process utilises an integrated module within the electronic case report form (CRF) system. Within both the high and low risk groups patients are 1:1 randomised to MTX versus ADA or AZA/6MP respectively in blocks of four stratified by EEN or steroid induction therapy. Code for randomisation is prepared and held by the central coordinating site and site co-ordinators are then informed of the results. Immunomodulator or biologic therapy should be commenced within 2 weeks of randomisation as per the protocol outlined in Table 2.

AZA/6MP and MTX are prescribed and dispensed according to local guidelines. ADA (Humira ®) is provided by AbbVie. Co-interventions are prohibited.

For peer review only

	Therapy	Route	Dose	Notes
**LOW RISK PROTOCOL**	Methotrexate	SC	15mg/m^2 body surface area weekly (max dose 25mg)	Ondansetron 4-8mg orally 1 hour pre injection and folic acid 15mg (5mg in patients <20kg) 3 days post injection are recommended for all patients
	**VERSUS**			
	Azathioprine	PO	2.5mg/kg (rounded down to nearest 12.5mg)	Half calculated dose for TPMT heterozygotes/activity 6-9nmol/h/ml
	**OR**			
	6-Mercaptopurine	PO	1.5mg/kg (rounded down to nearest 12.5mg)	Half calculated dose for TPMT heterozygotes/activity 6-9nmol/h/ml
**HIGH RISK PROTOCOL**	Methotrexate	SC	15mg/m^2 body surface area weekly (max dose 25mg)	Ondansetron 4-8mg orally 1 hour pre injection and folic acid 15mg (5mg in patients <20kg) 3 days post injection are recommended for all patients
	**VERSUS**			
	Adalimumab<br><br>(Humira ®)	SC	160mg then 80mg after 2 weeks then 40mg every 2 weeks thereafter (patients >35kg)<br><br>120mg then 80mg after 2 weeks then 40mg every 2 weeks thereafter (patients 25-35kg)<br><br>80mg then 40mg after 2 weeks and 20mg every 2 weeks thereafter (patients <25kg)	

Table 2: Medication protocol for low and high-risk patients following randomisation

(TPMT – thiopurine methytransferase)

For peer review only

Follow Up Visits (Visit 2, 3, 4, 5 and 6)

Patients are followed up at pre-specified intervals (Figure 1) with a window of +/- 2 weeks. A telephone call is undertaken at week 4 following initiation of induction in order to support patient compliance with induction regime and advise weaning where appropriate. Data as described in Box 3 are collected at each consultation. Patients' compliance with therapy is determined at each face-to-face follow up visit by pill and vial counts plus by patients' reporting.

Box 3: Standard requirements for each study visit

- An explicit history of illness since last visit, including review of symptoms, medications (including compliance check) and adverse events.
- Physical examination
- wPCDAI, PGA and PCDAI scoring
- Anthropometrics (height measured using a calibrated wall mounted stadiometer)
- Blood tests
    - White blood cells
    - Absolute neutrophil count
    - Haemoglobin
    - Haematocrit
    - Platelet count
    - Erythrocyte sedimentation rate (ESR)
    - C-reactive protein (CRP)
    - Amylase
    - Albumin
    - Aspartate transaminase (AST)
    - Alanine transaminase (ALT)
    - Conjugated bilirubin
    - Gamma glutamyl transferase (GGT)
- Stool samples for faecal calprotectin and microbiome analysis
- Health economic parameters (EQ-5D-Y proxy 1; EQ-5D-Y; EQ-5D-5L; WPAI:CD; school attendance questionnaire)
- Quality of life evaluation (IMPACT 3)
- Urine human chorionic gonadotropin (hCG) in all female patients of child-bearing potential
- Confirmation of contraception use or of absolute abstinence in all patients

Remission is defined as wPCDAI</=12.5, normal CRP (</= 1.5 times upper normal range) and being free of steroids or EEN. Once remission is achieved and induction therapy is discontinued, a patient is considered to be failing treatment or experiencing a relapse in the following circumstances:

- wPCDAI >40
- CRP >2 times upper normal limit in the absence of any clear infectious process
- wPCDAI >12.5 but <40 and/or CRP >1.5 times but <2 times over upper normal limit at 2 consecutive visits within 2-8 weeks
- Development of CD related complications e.g. fistulisation
- Requirement for additional CD-specific medication/surgery since last study visit

A patient will also be considered a treatment failure should induction therapy be continued at week 12. In addition, the treating clinician may escalate treatment at any time point independent of wPCDAI score if it is felt that the patient is experiencing a relapse.

For peer review only

Dose Optimisation and Therapeutic Drug Monitoring

Drug monitoring is undertaken as detailed below. In addition to this, samples for drug monitoring should be collected at the time of medication cessation in the event of drug discontinuance due to adverse effect or loss of response. Potential adaptations to therapies which may be made at specific follow up visits are detailed in Box 4.

Box 4 – Potential adaptations to therapies at follow up visits

Month 2 (Visit 2)

- Failure to discontinue induction therapy by week 12
  - Offer switch to the ancillary study (ADA STEP-up) to those prescribed MTX or AZA/6MP, or an increase in dose frequency to weekly in those prescribed ADA
  - Alternatively, the patient may leave the study and receive therapies as per the discretion of the treating clinician.

Months 4, 6, 9 and 12 (Visits 3, 4, 5 and 6)

- Thiopurine non response
  - Protocol as per metabolite levels (detailed in Table 3)
- Thiopurine intolerance (except pancreatitis)
  - Switch to alternate thiopurine (AZA to 6MP or vice versa) or split dose to provide twice daily (BD) dosing
- Thiopurine failure (any exacerbation despite dose optimisation/pancreatitis/cytopaenia)
  - Offer switch to ancillary study (ADA STEP-up) or exit study
- MTX intolerance or failure (any exacerbation or elevation of liver enzymes as detailed below)
  - Offer switch to ancillary study (ADA STEP-up) or exit study
- ADA failure (any exacerbation)
  - Increase frequency to weekly dosing

Azathioprine

RESULT	ACTION
6-TG <150	Consider non-compliance; repeat sample at subsequent visit and increase dose if low 6-TG confirmed (+25mg or +12.5mg if dose <50mg)
6-TG 150-800	No adaptation
6-TG >800	Decrease dose if repeat sample at subsequent visit confirms high 6-TG (-25mg or -12.5mg if dose <50mg)
6-MMP >8000 or signs of hepatotoxicity	Stop medication – switch to ancillary study<br><br>Erythrocyte lysate sample frozen at -80C and shipped to central lab at end of study for thiopurine nucleotides

Table 3 – Azathioprine dose adjustments based upon metabolite levels

TPMT genotype or phenotype at screening determines the initial dose of AZA/6MP; and measurement of thiopurine metabolites (6-thioguanine (6-TG) and 6-methylmercaptopurine (6-MMP)) at visit 2 determines requirement for subsequent dose adjustment performed according to the recommendations in Table 3. Where possible thiopurine metabolites are measured locally; central lab measurements are provided for centres where this is unavailable.

At visit 2 a urine sample for TPMT metabolite determination and an erythrocyte lysate sample for quantification of Thiopurine Nucleotides by Liquid Chromatography-Tandem Mass Spectrometry should be frozen at -80°C and shipped on dried ice to the central lab at the end of the study. At each visit from visit

For peer review only

2 to 6, an additional EDTA blood sample will be collected for further 6-TG and 6-MMP testing and frozen at -80C.

Methotrexate

Washed erythrocyte for MTX levels will be obtained at visits 2, 4 and 6 and stored frozen at local centres. These samples will be sent on dry ice for central analysis to evaluate response to therapy and adverse effects in relation to drug levels.

Adalimumab

Adalimumab trough levels are measured after 3 injections of maintenance therapy (e.g. at visit 2) within the local laboratory (central lab testing available if local lab testing is unavailable). Dosing interval may be shortened to weekly in the event of low ADA levels (<8 mcg/ml) and negative ADA antibodies. Further samples should be obtained at visits 3, 4, 5 and 6 and should be frozen for later analysis within the central lab.

Pharmacogenetics

DNA for pharmacogenetics should be taken from patients randomised to MTX or AZA/6MP for multiplex genotyping of polymorphism related to drug metabolism to evaluate safety and response to therapy. Analysis will be performed at the end of the study, or earlier in those patients showing toxicity.

For peer review only

Ancillary Study

Patients unable to discontinue induction therapy or those randomised to thiopurine or MTX therapy who experience treatment failure may be invited to participate in the ancillary study (STEP-up ADA) until visit 5. Any initial maintenance therapy will be stopped and induction and maintenance regime for ADA as previously described will be commenced. Up to 3 additional study visits at 3-month intervals will be offered to these patients in order to obtain 12 months of follow up. A maximum of 68 patients can participate in this ancillary study allowing a 1:1 comparison of TOP-down ADA to STEP-up ADA therapy.

Unscheduled Visits

Unscheduled visits may be arranged based upon clinical requirements. As for scheduled visits per protocol treatment adaptions are possible if intolerance or failure of the study drug is detected. Subsequent scheduled visits will not be changed after an unscheduled visit.

Treatment Discontinuation

Patients who discontinue treatment before completing 12 months of study drug within either the main study or the ancillary study will receive a single follow-up visit. This will be either 12 months after the commencement of study treatment or at the point of inclusion in the ancillary study.

Modifications to the protocol while the study is being conducted will be relayed to all site staff by email and then onto their relevant ethical and regulatory boards. The current manuscript is based on protocol 5.1 last modified 28th May 2019.

For peer review only

Allocation Concealment and Blinding

For ethical reasons we decided against a double dummy design for blinding the patient, parents and care givers. Due to the differences in medication administration route and the significant nausea commonly associated with MTX blinding of the allocation to the patients, their families or their physicians is not possible. Where possible however, blinding of an alternative clinician to score the wPCDAI, PCDAI and PGA at each study visit should occur (prospective randomized open blind end-point (PROBE) evaluation).

Safety

The external and independent Advisory Board of PIBDSETQuality serves as an independent Data and Safety Monitoring Board which meets at pre-specified intervals with access to all data within the study. The principal investigator at each site is responsible for reporting any safety issues (adverse events, serious adverse events (SAEs), suspected unexpected serious adverse reactions), drop-outs, or any new information which may impact the study in any way. The principal investigator shall report to the sponsor all SAEs experienced by a study subject receiving an Adalimumab (Humira) within 24 hours of learning of the event regardless of the relationship of the event to the product. All SAEs are immediately sent to AbbVie pharmacovigilance by the sponsor. SAEs will be followed from the date of patient's signature of informed consent, until complete resolution or 30 days after the end of the study/patient's final study visit.

For peer review only

Box 5 – Criteria for premature termination of study treatment or participation

- Pregnancy at any stage
- Treatment failure as per protocol
- Failure to tolerate allocated treatment or alternatives as listed within the protocol
- Significant drug related side effects manifesting as significantly abnormal bloods results or adverse effects based upon the clinical judgement of the treating physician
- Request of participant to be withdrawn from treatment
- The judgement of the treating physician being that it is in the best interests of the participant to withdraw from study treatment
- Loss of participant to follow up
- Patient death

Participants may withdraw consent for further participation or data collection at any time without giving reason and without prejudicing further care or treatment. Patients will be permanently withdrawn from study treatment in the event of any of the situations outlined in Box 5. Patients should be provided with a study alert card for use in the event of an emergency.

Biochemical markers are monitored with a clearly defined protocol for adjustments to therapy based on abnormal results (e.g. neutropenia, pancreatitis, elevated liver enzymes).

Data Collection, Management and Monitoring

Patient CRFs are completed in a prospective manner using an electronic web-based system designed specifically by PIBDnet for this trial. In order to maintain data

For peer review only

security and integrity, the web-based data entry will be linked to a password secured Microsoft Access database, where data will be stored until time of analysis. Files will be saved on a code secured net-drive and backed-up following each data entry on a disk locked in a cabinet. Patients will be identified only by a study code assigned at the point of enrolment. Code of patient identifiers will be kept at each participating site. Handling of patient-identifiable is compliant with the legislation of each participating centre and the European General Data Protection Regulation (GDPR). Investigators will be invited to fax or email the paper source document to the coordinating site on a random basis to allow appropriate monitoring. Access to data with detailed information on study outcomes will be made available to other research groups on request and at the discretion of the principal investigators.

Monitoring arrangements are in place for all sites after initial site initiation. The monitoring visits will occur regularly partly dependant on recruitment rate at individual sites. The monitoring is perfomed usually by someone external to the clinical team.

Analysis and Statistical Methods

Descriptive statistics (mean, median, standard deviation, standard error, quartiles, minimum, maximum, and two-sided 95% confidence limits of mean and median) will be presented for each treatment of the low and high risk paediatric CD groups and where applicable, for the paired difference of each patient. Frequency tables will be presented where applicable.

Primary Analysis

For peer review only

Difference in the 12-month steroid/EEN free sustained remission rates between the treatment groups will be undertaken using Chi square test. Mantel Haenzel test will be used to combine data from all participating sites.

Secondary Analyses

Chi-square tests or Fisher's exact tests will be used to compare rates of remission, steroid intake, dropout and serious adverse events between the two arms of each risk group and between the low and high risk MTX groups. Logistic regression analyses may be performed to adjust for any imbalances in baseline covariates. To compare time to disease flare between the arms of each risk group and between high and low risk MTX groups, a Kaplan–Meier survival estimate will be used and the log-rank test of equality over strata. A Cox proportional hazard model will be constructed to obtain a hazard ratio after validation of the proportionality assumption and adjusting for possible confounding variables (including age and disease duration). Student's t tests or Wilcoxon rank sum tests will be used to compare growth, steroid dose, adverse events, changes in quality of life and patient reported outcomes between the two arms of each risk group and between the high and low risk MTX groups. The predictive value of faecal calprotectin levels, CRP, serum tests or other clinical predictors for response (including genomic and serological markers) will be assessed for each arm of the study using sensitivity, specificity, negative and positive predictive values or area under the ROC curve. Multivariate logistic regression analyses will then be performed.

For peer review only

Analyses will be performed using the R software (http://cran.r-project.org). All comparisons will be made using a 2-sided significance level of 0.05.

Sample Size Considerations

Estimated remission rates are based on recent analysis from the RISK study[18], indicating an advantage of early anti-TNF introduction over immunomodulator therapy. For the low risk group, it was hypothesized that 48% of children will be in remission at 12 months for the AZA/6MP arm versus 70% for the MTX arm. On the basis of this data with an alpha risk of 5% and a power of 80% a sample requirement of 88 patients per arm was calculated assuming a 10% loss of follow up. For the high-risk group, it was hypothesised that 40% of children will be in remission at 12 months for the MTX arm versus 65% for the adalimumab arm. To detect this difference with an alpha risk of 5% and a power of 80%, a sample size of 68 participants is necessary, again assuming a 10% loss of follow-up. In total 312 participants will be included in the study (176 low-risk group; 136 high risk group).

Patient and Public Involvement

Patients were not involved in the development of this study; however, the French patient charity AFA Crohn, RCH, France was involved in study design and critically reviewed and commented upon all aspects of the trial.

For peer review only

Discussion

REDUCE-RISK in CD is the first multicentre international RCT aiming to compare three different medication strategies for maintenance of remission in newly diagnosed CD based upon a risk stratification protocol. During the 12-month follow up period the effects of the differing management strategies will be assessed via data collected and outcome measures as defined above in order to analyse the efficacy and safety of each medication and better define the most appropriate first-line maintenance immunomodulators to be used in specific subsets of CD patients. As a group we speculatively hypothesise that MTX will be superior to thiopurines for maintaining remission in CD in the low risk group although in the absence of head to head studies prior to this one this study will provide randomised data to address this. We also hypothesise that ADA will be superior to MTX in the high-risk group based upon the results from the RISK study.[18] In addition to this, the ancillary study will compare outcomes in ADA treated patients from inclusion (Top-down) versus patients switched to ADA due to failure of immunomodulators (Step-up), with the potential to stratify which patients might benefit from such a top-down treatment strategy. We acknowledge that comparison of the ancillary group with the group randomised from baseline to ADA is not randomised and may be subject to selection bias noting the ancillary group have failed or been intolerant to initial therapy. However we feel it is important to include this to allow us to compare the trial with studies which have allocated patients directly to anti-tnf (RISK, TISKids) and to see how many patients benefit from “rescue therapy” after failure of their initial allocation.

For peer review only

The design and completion of interventional studies in PIBD is a recognised challenge between rigorous study design methodology and pragmatic considerations around feasibility and completion within a paediatric dataset.[23] This particular study is limited by the inability to blind the treatment allocation to the patients, their families or their treating physician due to the differences in medication administration route and the side effects commonly associated with the study medication. Although the protocol advises that where possible blinding of an alternative clinician to score disease assessment at each study visit should occur in order to obtain prospective randomized open blind end-point (PROBE) evaluation this may be practically difficult in smaller centres where staff are familiar with the majority of their patient cohort.

ETHICS AND DISSEMINATION

The study is being conducted according to the principles of the Declarations of Helsinki and to date has been approved by all participating sites as listed within supplementary Table 1. Clinical trials authorisation and ethics approval has been obtained from the local ethics review committees of these participating nations and centres. The Standard Protocol Items: Recommendations for Interventional Trials (SPIRIT) guidelines[24] were adhered to in the production of the protocol for this trial (see uploaded material for details).

Consent

For peer review only

Patients and their caregivers are provided with study-specific information including an explicit description of the study outline and alternatives for participation. It is made clear to all patients approached that declining to participate in the study will not jeopardize the quality of subsequent care received. After a period of consideration, if agreeable, the patient's parent or caregiver is asked to sign consent forms with age-appropriate assent obtained from the child where relevant (see appendix 1 for model consent forms). The signed forms are filed within the patient's medical record with a copy provided to the participant and their caregiver. Consent will be obtained by site staff with the relevant training and who are identified as assigned on the delegation log. Participants taking part in the ancillary study will not be re-consented.

Dissemination

Results of the study will be submitted for publication within a peer-reviewed journal. In accordance with the H2020 general grant agreement, the dissemination process will ensure open access to the scientific publications resulting from this project. Journal authorship guidelines will be adhered to and there are no plans to use professional writers.

AUTHOR CONTRIBUTIONS

RH prepared the draft manuscript with comments and review from all authors. RKR, MA, LR, NC, SK, AL, DT, GV, MN and LB and FMR were involved in the conception, design, planning and then drafting of the original research protocol and

RKR, RH, MA, LR, NC, SK, AL, DT, GV, MN and LB and FMR provided critical review of the manuscript and approved the final uploaded draft.

As sponsor PIBDnet has full responsibility and control for the original study design, collection, management, analysis, and interpretation of data, including writing of the report and the decision where to submit the report for publication,

FUNDING

This work was supported as part of the PIBD-SETQuality (Paediatric Inflammatory Bowel Diseases Network for Safety, Efficacy, Treatment and Quality improvement of care) project funded by the European Commission Horizon 2020 - Research and Innovation Framework Programme (grant number 668023). ADA (Humira ®) is provided by AbbVie.

The main study sponsor is PIBDNet. PIBDNet is the EU legal representative for the study. The specific contact for the sponsor is Frank Ruemmele (Service de Gastro-entéroloegie, Hôpital Necker Enfants Maldes, 149 rue de Sèvres, 75015 Paris, France).

COMPETING INTERESTS

RKR is supported by an NHS Research Scotland Senior Research Fellowship, and has received speaker's fees, travel support, and/or participated in medical board meetings with Nestle, MSD Immunology, AbbVie, Dr Falk, Takeda, Napp, Mead Johnson, Nutricia & 4D Pharma. FMR has received speaker fees from Shering-Plough, Nestlé, MeadJohnson, Ferring, MSD, Johnson & Johnson, Centocor, AbbVie; has served as a board member for SAC:DEVELOP (Johnson & Johnson), CAPE (AbbVie), LEA (AbbVie); and has been invited to MSD France, Nestlé Nutrition Institute, Nestlé Health Science, Danone, MeadJohnson, Takeda, Celgene, Biogen,

For peer review only

Shire, Pfizer, and Therakos. DT received consultation fee, research grant, royalties, or honorarium from Janssen, Pfizer, Hospital for Sick Children, Ferring, Abbvie, Takeda, Biogen, Atlantic Health, Shire, Celgene, Lilly, Neopharm, Roche. LdR received consultation fee, research grant, or honorarium from ZonMw, ECCO, Shire, Malinckrodt, Nestlé, Celltrion, Abbvie and Pfizer. MA received consultation fee and honorarium from Abbvie. SK received consultation fee, research grant, or honorarium from Danone, Nestec-Nutrition, Abbvie, Takeda, Celgene, Shire, Pfizer, Biogaia, Janssen, Berlin-Chemie; Mead Johnson, Vifor, Pharmacosmos, ThermoFisher

Remaining authors: nil competing interests declared.

FIGURE LEGENDS

Figure 1 – Study Design of the REDUCE-RISK in CD trial

M2 = Month 2, V2 = visit 2.

For peer review only

REFERENCES

1. Ruemmele FM, Veres G, Kolho KL et al. Consensus guidelines of ECCO/ESPGHAN on the medical management of pediatric Crohn's disease. *J Crohns Colitis*. 2014;8(10):1179-207.

2. Escher J. Budesonide versus prednisolone for the treatment of active Crohn's disease in children. *Eur J Gastroenterol Hepatol*. 2004;16(1):47-54.

3. Heuschkel R, Menache C, Megerian J et al. Enteral Nutrition and Corticosteroids in the Treatment of Acute Crohn's Disease in Children. *J Pediatr Gastroenterol Nutr.* 2000;31(1):8-15.

For peer review only

4. Chande N, Patton P, Tsoulis D et al. Azathioprine or 6-mercaptopurine for maintenance of remission in Crohn's disease. *Cochrane Database Syst Rev*. 2015;(10):CD000067

5. Markowitz J, Grancher K, Kohn N et al. A multicenter trial of 6-mercaptopurine and prednisone in children with newly diagnosed Crohn's disease. *Gastroenterology*. 2000;119(4):895-902.

6. D'Haens G, Geboes K, Rutgeerts P. Endoscopic and histologic healing of Crohn's (ileo-) colitis with azathioprine. *Gastrointest Endosc*. 1999;50(5):667-71.

7. Feagan BG, Fedorak RN, Irvine EJ et al. A comparison of methotrexate with placebo for the maintenance of remission in Crohn's disease. North American Crohn's Study Group Investigators. *N Engl J Med*. 2000;342(22):1627-32.

8. Feagan BG, Rochon J, Fedorak RN et al. Methotrexate for the treatment of Crohn's disease. The North American Crohn's Study Group Investigators. *N Engl J Med.* 1995;332(5):292-7.

9. Willot S, Noble A, Deslandres C. Methotrexate in the treatment of inflammatory bowel disease: An 8-year retrospective study in a Canadian pediatric IBD center. *Inflamm Bowel Dis*. 2011;17(12):2521-2526.

For peer review only

10. Patel V, Wang Y, MacDonald J et al. Methotrexate for maintenance of remission in Crohn's disease. *Cochrane Database Syst Rev*. 2014;(8):CD006884

11. Hyams J, Crandall W, Kugathasan S et al. Induction and maintenance infliximab therapy for the treatment of moderate-to-severe Crohn's disease in children. *Gastroenterology*. 2007;132(3):863-73.

12. Ruemmele F, Lachaux A, Cézard J et al. Efficacy of Infliximab in Pediatric Crohn’s Disease: A Randomized Multicenter Open-Label Trial Comparing Scheduled to On Demand Maintenance Therapy. *Inflamm Bowel Dis*. 2009;15(3):388-394.

13. Hyams JS, Griffiths A, Markowitz J et al. Safety and efficacy of adalimumab for moderate to severe Crohn's disease in children. *Gastroenterology*. 2012;143(2):365-74.e2.

14. Russell R, Wilson M, Loganathan S et al. A British Society of Paediatric Gastroenterology, Hepatology and Nutrition survey of the effectiveness and safety of adalimumab in children with inflammatory bowel disease. *Aliment Pharmacol Ther*. 2011;33(8):946-953.

15. Ardizzone S, Bollani S, Manzionna G et al. Comparison between methotrexate and azathioprine in the treatment of chronic active Crohn's

For peer review only

disease: a randomised, investigator-blind study. *Dig Liver Dis*. 2003;35(9):619-27.

16. Oren R, Moshkowitz M, Odes S et al. Methotrexate in chronic active Crohn's disease: a double-blind, randomized, Israeli multicenter trial. *Am J Gastroenterol*. 1997;92(12):2203-9.

17. Maté-Jiménez J, Hermida C, Cantero-Perona J et al. 6-Mercaptopurine or methotrexate added to prednisone induces and maintains remission in steroid-dependent inflammatory bowel disease. *Eur J Gastroenterol Hepatol.* 2000;12(11):1227-1233.

18. Walters TD, Kim MO, Denson LA et al. Increased effectiveness of early therapy with anti-tumor necrosis factor-alpha vs an immunomodulator in children with Crohn's disease. *Gastroenterology*. 2014;146(2):383-91.

19. Torres J, Caprioli F, Katsanos KH et al. Predicting outcomes to optimize disease management in inflammatory bowel diseases. *J Crohns Colitis*. 2016;10(12):1385-1394

20. Cozijnsen M, van Pieterson M, Samsom J et al. Top-down Infliximab Study in Kids with Crohn's disease (TISKids): an international multicentre randomised controlled trial. *BMJ Open Gastroenterol*. 2016;3(1):e000123.

For peer review only

21. Levine A, Koletzko S, Turner D et al. The ESPGHAN Revised Porto Criteria for the Diagnosis of Inflammatory Bowel Disease in Children and Adolescents. *J Pediatr Gastroenterol Nutr*. 2013;58(6):795-806.

22. Levine A, Griffiths A, Markowitz J et al. Pediatric modification of the Montreal classification for inflammatory bowel disease. *Inflamm Bowel Dis.* 2011;17(6):1314-1321.

23. Turner D, Griffiths AM, Wilson D et al. Designing clinical trials in paediatric inflammatory bowel diseases: a PIBDnet commentary. *Gut*. Published online first: 12 April 2019 Apr. doi:10.1136/gutjnl-2018-317987

24. Chan A, Tetzlaff J, Altman D et al. SPIRIT 2013 Statement: Defining Standard Protocol Items for Clinical Trials. *Ann Intern Med*. 2013;158(3):200-207.

For peer review only

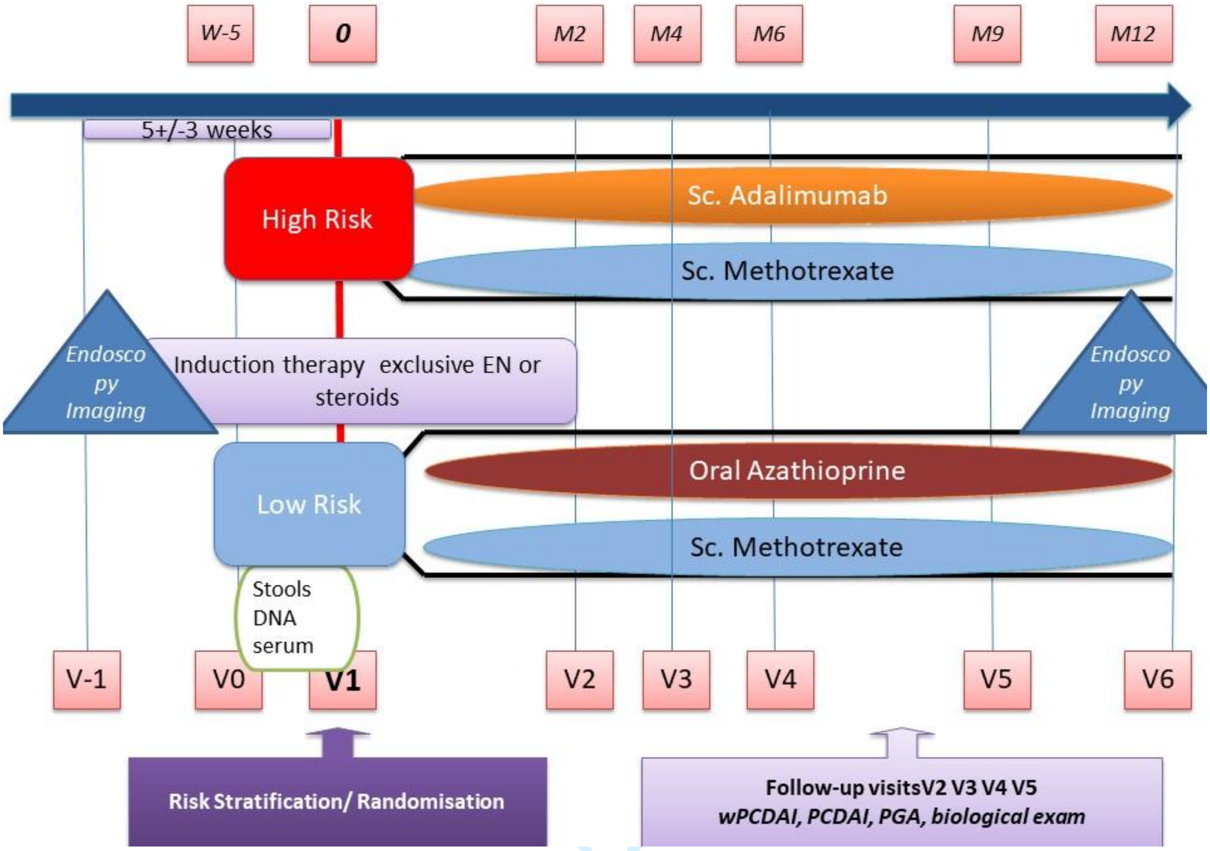

Figure 1 – Study Design of the REDUCE-RISK in CD trial

Participating Sites – REDUCE-RISK in CD Study			
**Country**	**City**	**Site**	**Ethics committee**
**Belgium**	Brussels	Universitair Ziekenhuis	Commissie Ethiek, UZ **Brussel**
	Brussels	Clinique Saint Luc UCL	
	Liège	Clinique de l'Espérance	
	Brussels	HUDERF	
**Canada**	Toronto	SickKids	SickKids Research Ethics Board, **Toronto**
**Czech Republic**	Prague	FN Motol	Eticka Komise, **Plzen**<br>Eticka Komise, VFN **Praha**<br>Eticka Komise, FN Motol **Praha**
	Plzeň	FN Plzeň	
	Prague	First Medical Faculty	
**France**	Paris	Hôpital Necker Enfants Malades	CPP Hôpital Necker, **Paris**
	Paris	Hôpital Robert Debré	
	Paris	Hôpital Armand Trousseau	
	Le Havre	Hôpital Jacques Monod	
	Nancy	Hôpitaux de Brabois	
	Toulouse	Hôpital des Enfants	
	Tours	Hôpital Clocheville	
	Caen	CHU Caen Côte de Nacre	
	Marseille	Hôpital de la Timone	
**Germany**	Munich	Childrens Hospital	Ethikkommission LMU, **München**
	Ulm	Universitätsklinikum	
	Hannover	MHH Kinderklinik	
	Giessen	UKGM	
	Berlin	Charite Hospital	
**Greece**	Athens	Children’s Hospital “AGIA SOFIA”	Ethics Committee, **Athens**
**Israel**	Jerusalem	Shaare Zedek Medical Center	Helsinki Committee, Schneider Medical Center, **Petah Tikva**<br>Ethics and Research Committe, Wolfson Medical Center, **Tel Aviv**<br>Institutional Review Board, SZMC, **Jerusalem**
	Tel Aviv	Wolfson Medical Center	
	Petah Tikva	Schneider Children's Medical Center	
	Ramat Gan	Sheba Medical Center	
	Haifa	Rambam Medical Center	
**Italy**	Rome	Università degli Studi di Roma La Sapienza	Comitato Etico dell'Universita' "SAPIENZA", **Roma**<br>Comitato Etico Regionale per la Sperimentazione Clinica della Regione Toscana Sezione, **Firenze**<br>Comitato Etico, Servizio Saintario Regionale, **Bologna**
	Bologna	Maggiore Hospital	
	Florence	Azienda Ospedaliero Universitaria	
	Parma	Azienda Ospedaliero	
	Rome	Opsedale Pediatrico Bambino Gesù	
**Netherlands**	Rotterdam	Erasmus Medical Center	Medische Etische Toetsings Commissie, Erasmus MC,

For peer review only

			**Rotterdam**
**Poland**	Warsaw	Centrum Zdrowia MDM	Bioethical Commission at the Institute of Polish Mother's Health Center, **Lodz**
	Białystok	Uniwersytecki Dziecięcy Szpital Kliniczny	
	Łódź	Instytut Centrum Zdrowia Matki Polki	
**United Kingdom**	Glasgow	The Royal Hospital for Children	North West - Liverpool East Research Ethics Committee, NHS, **Manchester**
	London	Royal London Children's Hospital, Barts Health NHS Trust	
	Edinburgh	Sick Children's Hospital	
	Birmingham	Children's Hospital	
	Oxford	John Radcliffe Hospital	

Supplementary Table 1 – Sites participating in REDUCE-RISK in CD

For peer review only

PIBD-SETQuality

European Commission

INFORMED CONSENT FORM

Parents/Guardian Informed Consent Form for Participation of a Minor in a Clinical Trial

Risk-stratified randomized controlled trial in paediatric Crohn's Disease: Methotrexate versus azathioprine or adalimumab for maintaining remission in patients at low or at high risk for aggressive disease course, respectively – a treatment strategy.

Dear parents,

Your child's doctor, Dr………………………., working at ………………………………….. Hospital, propose your child to participate in a clinical trial related to its disease.

It is important to read this note carefully before taking any decision. Do not hesitate to ask the physician all the questions you may have about it.

The participation of your child is based on volunteering. Therefore, your child can refuse to participate or stop its participation in the trial at any time, all of this without prejudice to the patient's right to receive the standard treatment.

If you refuse your child to participate, he/she will still receive the best medical support.

Purpose of the research and trial's objectives

Your child has just been diagnosed with Crohn's disease. The disease is caracterised by chronic inflammation of the digestive track (bowel/colon). This disease changes over between remission period and relapse period. There are efficient drugs able to prevent relapse and to maintain remission. In order to reduce the likelihood of long-term complications, induction treatment has already been prescribed to your child. This first treatment has to be followed up by a maintenance treatment that will be introduced to avoid the inflammation from returning. Consensus guidelines of ECCO / ESPGHAN (french and european IBD specialized organizations) recommend 3 efficient treatments: either immunosuppressive treatment with Thiopurines (azathioprine and 6-mercaptopurine), or Methotrexate or anti TNF (adalimumab).

So far, no clinical trial has been conducted to compare those 3 treatments in children with Crohn's disease and to answer the following question: "Which treatment is the most efficient, for which patient and/or in which situation?"

Progression of Crohn's disease is not the same for all patients. That's why this study will first classified all children in high and low risk groups based on more or less severe course of Crohn's disease. The lower risk group will be randomized (which is like tossing a coin) to receive either thiopurines or methotrexate as maintenance treatment. The high risk group will be randomized to receive either methotrexate or adalimumab. Results will show whether there is different efficiency between the 3 drugs for patients with a more or less severe disease.

Sponsor

PIBD-Net (www.pibd-net.org) is a global, international and non-profit organisation gathering physicians and researchers specialised in inflammatory bowel diseases. The acronym stands for Pediatric Inflammatory Bowel Diseases Network and it is present in 31 countries (Europe, North America, Australia and Japan). This organisation is dedicated in improving the medical care of children with inflammatory bowel disease through the establishment of clinical researches.

PIBD-Net and partners received funding from European Commission for Horizon 2020 program (project no.668023) in order to perform this research.

The approximate number of participants and duration of follow-up

A total of 312 new-onset children with Crohn's disease (136 in that high-risk group and 176 in the low risk) will be enrolled in many sites around the world. The period of recruitment is 45 months and your child will be followed up for 12 months after enrolment.

For peer review only

For peer review only - http://bmjopen.bmj.com/site/about/guidelines.xhtml

PIBD-SETQuality

What will happen to your child during the trial?

If you agree to have your child participating in this study, your child will be first directed into one of two groups based on certain predictors of its disease (such as its location and severity). You will know which group your child is in. Next, your child will be randomized to receive maintenance treatment namely:

- **METHOTREXATE or AZATHIOPRINE for the low risk group** ;
- **METHOTREXATE orADALIMUMABfor the high risk group** ;

You cannot choose the treatment group but you will know which drug your child will receive.

You will not be asked to come to clinic just because of this study which is designed to mirror regular follow-up in clinic. After signing the informed consent allowing your child to be part of this research, your child will have clinic visit every 2 months during the first 6 months and then every 3 months during the last 6 months. At each visit, a clinic examination is performed and blood, urine and stool samples are collected (this process follows our standard clinical practices of our patients not involved in this protocole).

Your doctor, your child and yourself will be asked to complete short questionnaires to evaluate the quality of life of your child. Most of the recorded data for this study is needed anyway as part of a regular visit but there might be a few more questions we will ask you for this study.

We will also contact you over the phone at week 4 to ask how your child feels regarding its Crohn's disease, whether your child has any bad reactions to the medications your child will receive and check your child compliance to the treatment.

To optimize the medications we prescribe, we will draw 12 ml (3-4 teaspoons) of blood at inclusion visit, 10ml at visit V2, and then 5ml at each next study visits to measure the level of the medications your child will receive. A urine sample will be collected at inclusion visit and a DNA sample (either 5ml of blood or buccal swab) will be collected at the beginning of the study, and also in case of drug intolerance.

We will also collect your child stool (poop) six times during the year to measure the amount of inflammation in its bowel as well as bacteria flora in its intestine.

We will evaluate whether the drugs work based on

- completed questionnaires
- clinic examination
- results of biological samples

Optional Ancillary study (« ADA STEP-up »)

In case of failing (intolerance or relapse) of your child immunomodulator therapy (: either azathioprine/6MP or methotrexate), your child will be invitated to participate in the ancillary study. If you agree, your child will be prescribed adalimumab during 12 months.

This adalimumab treatment can increase the study duration by a maximum of 9 months, meaning a maximum of 3 additional visits. Those visits are identical to the regular follow up study visits.

The expected benefits to the participant or to others because of the trial

The medications your child will receive in this study are not experimental and thus there are no direct benefit for using these drugs that are available outside of the study. However, your child will be monitored closely to ensure optimization of the treatment by adapting drug amounts based on new analyses (urine, DNA, blood and stool samples). In addition, patients involved in this study have access to molecular analyses in order to better understand why a patient is less responsive than expected. That might result in a more tight control of the disease and better monitoring of the treatment of your child.

For peer review only

For peer review only - http://bmjopen.bmj.com/site/about/guidelines.xhtml

PIBD-SETQuality

European Commission

After study completion, we will have the required data to recommend how to use these medications in new children who develop Crohn's disease.

Risks added by the research

As previously mentionned, all medications used in this trial are not experimental and are being used very often in clinical practice in children/adolescents and adults with Crohn's disease. There is no additional risk compares to regular clinical practices. The known risks and discomfort that may be anticipated are listed below.

Known risks and discomfort that may be anticipated

The medications yourchild will receive in this study are not experimental but used in regular practices. They can also be associated with side effects (all described in the corresponding drug information sheet).

- ✓ **METHOTREXATE: weekly subcutaneous (under the skin)** This drug may be associated with side effects mainly in the day of the injection including flu-like symptoms, nausea, vomiting, headache or fatigue. Your child will be asked to take a vitamin called folic acid which will reduce these non-dangerous side effects. This injection may cause slight discomfort.METHOTREXATE may cause reduced blood counts, especially white blood cells and elevated liver enzymes. Thoses parameters will be checked regularly. In this study, molecular analyses will be performed to screen patients who can't tolerate METHOTREXATE..This drug causes an unusual sensitiveness to the sun (however, there is no data stating that it increases the risk of cancer or lymphoma). METHOTREXATE can cause foetal abdormalities so pregnancy is not allowed and efficient contraceptive is essential (for both male and female).
- ✓ **AZATHIOPRINE (or 6MP): to be taken orally.** THIOPURINES may cause reduced blood counts, especially white blood cells and elevated liver enzymes. Thoses parameters will be checked regurlarlyIn this study, molecular analyses will be done in order to screen patients who can't tolerate those drugs. In some rare cases (<3%), the drug may cause inflammation in the pancreas which is usually mild and not dangerous. As all drugs, THIOPURINES can't be tolerated by some patients due to allergy. Approximately 10% of children will not tolerate the drug because of nausea, vomiting, tummy pain, diarrhea, headaches or fever..THIOPURINES are associated with an increased infectious risk (about 1%). Infections are likely cause by viruses. In rare cases, THIOPURINES may increase risk for blood cancer called lymphoma (especially for patients > 65 year old).–This drug causes an unusual sensitiveness to the sun and can be associated with skin cancer in case of significant sun exposure.

ADALIMUMAB: subcutaneous (under the skin) injections every 2 weeks Adalimumab is associated with minor pain during the injection and local reactions could appear with minimal significance..ADALIMUMAB is associated with an increased infectious risk. However, serious infections are uncommon. Before starting ADALIMUMAB treatment, tuberculosis must be excluded. With time, the effect of adalimumab may wain as a result of the development of antibodies against the drug. Skin inflammatory damages (such as « psoriasis ») were observed in some patients. Anti TNF drugs have been closely monitored since their use as standard treatment. Those drugs may be responsible of heart failure for patients with severe heart disease, hepatitis, decreasing blood cells, demyelinating neurologic disease, or lupus (without affecting main organs). In addition, some cases of cancer have been notified in patient treated by ADALIMUMAB, but risks of cancer is slightly increased only for melanoma. Number of cancer seems not to be increased compared to patients with Crohn's diseases and without being treated with those drugs.

Circumstances under which participation in the medical trial may be discontinued in accordance with the decision of the investigator or the Sponsor:

a. The doctor has the right to take your child out of the study at any time. This will be made after clinical considerations, your child's side effects from the drugs, intolerance to the drugs or lose of response.

b. Regulatory authorities (Ministery of Health or Ethics committee), may stop your child participating in the study.

For peer review only - http://bmjopen.bmj.com/site/about/guidelines.xhtml

PIBD-SETQuality

European Commission

An explanation of alternative treatments, their advantages and disadvantages, if any, for the participant:

The current standard therapy for maintenance therapy in Crohn's disease is either METHOTREXATE or thiopurines or anti-TNF biologics for the more severe Crohn diseaes. This is exactly the medications given also as part of this study. The difference is that instead letting you and the doctor choose between the options, the choice is standardized based on predictive variables of your disease and randomization. If you choose not to have your child participating in the study, your child will likely receive anyway one or more of these three drugs. The only exception would be that if anti-TNF is prescribed, either adalimumab or infliximab can be given and as part of this study your child will receive adalimumab only. However, your child will not have access to molecular analyses described in this protocole with a close monitoring of drug safety. Indeed, those 'new" analyses are not done in the standardclinical practise of Crohn's disease.

If you participate in this study, what will you have to do more than usual ?

If you agree to have your child participating in this study, please make sure to follow the listed points belowPlease come at your appointements with your child. If not possible, please inform its physician as soon as possible

- Please ensure that your child takes the treatment as instructed by its doctor
- Please inform the physician involved in the study of any event happening during the research (such as hospitalization,…)
- Your child must not participate in any other clinical trial that involves the use of an investigational product throughout the course of this trial. It is to avoid accidents such as possible interactions between medicines.

Biological samples collected during this research project

If you agree to have your child participating in this research, additional blood, urine and stool samples will be collected at the same time as our standard clinic samplings. Please see below:

- ✓ 10ml of blood during inclusion visit (on randomisation day).
- ✓ 5ml of blood (PAX tube) during inclusion visit for RNA analyses
- ✓ 10ml of blood at follow up visits M2 (2 months after inclusion)
- ✓ 5 ml of blood at follow up visits M4, M6, M9 and M12 (4, 6, 9, 12 months after inclusion)
- ✓ Stool sample at inclusion visit and follow up visits M2, M4, M6, M9 and M12 (2, 4, 6, 9, 12 months after inclusion visit).
- ✓ A DNA sample will be collected at inclusion visit and in case of intolerance of one of the drugs for DNA analyses.
- ✓
- ✓ Urine sample (15ml) will be collected at M2 visit (2 months after inclusion).

Those samples will be sent to specialized laboratories in order to be used to perform specific studies such as adalimumab, methotrexate, thiopurine analyses and serology, genetic (both DNA and RA), microbiology studies. They will also be re-used for further testing on Crohn's disease, its diagnosis and its treatment as well as efficacy and tolerance by molecular ("omic") analyses.

At any time, you can request to your clinician to have those biological samples destroyed or not to be used for further researches.

Confidentiality

As part of biomedical research in which PIBD-Net sponsor proposes your child's participation, treatment of personal data will be set up to analyse results of this research based on its aim. Therefore, your child medical data and quality of life will be transferred to PIBD-Net sponsor. Those data will be anonymous and identified by a coded number and its initials. Those confidential data could be transferred to local and foreign authorities. If your child has to be withdrawn for any reasons, collected data prior its withdrawal will be used unless you do not want

For peer review only - http://bmjopen.bmj.com/site/about/guidelines.xhtml

PIBD-SETQuality

European Commission

them to. Then, you will have to inform the physician accordingly.

According to the EU General Data Protection Regulation (GDPR) dated on 26May2018, you have the right to access to your child and your personal data, modify them and oppose the use of your child and yourdata. You have also the right to request that your child and your personal data are erased, are limited in use, and to ask for a complete copy of all data collected from you and your child for the study. You can contact the Data Privacy Officer (DPO) of the sponsor at any time at dpo@pibd-net.org for any request regarding your child and your personal data.

Data collected for the study are transferred outside of the EU, as our database is based in Israel. However, we guarantee that data protection will be as strict as requested by GDPR.

Voluntary participation

Your participation in this research is entirely voluntary. It is your choice whether to have your child participating or not, all the services your child receives at this hospital will continue and nothing will change. If you choose not to participate in this research project, your child will be offered the treatment that is routinely offered in this hospital for Crohn's disease. You may change your mind later and stop participating even if you agreed earlier.

Right to refuse or withdraw

Your child does not have to take part in this research if you do not wish to do so and refusing to participate will not affect its treatment in any way. Your child will still have all the benefits that it would otherwise have at this hospital. You may stop participating in the research at any time that you wish without losing any of its rights as a patient here. Its treatment at this hospital will not be affected in any way.

Alternatives to participating

If you do not wish that your child takes part in the research, your child will be provided with the established standard treatment available at this hospital.

Reimbursement

There is no reimbursement for participating in this study. There are no special visits to the hospital excepted during this study. All DNA, blood, urine and stool samples will be taken at the time of a routine clinic visit.

This proposal has been reviewed and approved by [name of the local IRB], which is a committee whose task it is to make sure that research participants are protected from harm.

For peer review only

For peer review only - http://bmjopen.bmj.com/site/about/guidelines.xhtml

PIBD-SETQuality

European Commission

Informed consent form

We, the undersigned :

M, Miss, *(name, first name of parent/legal guardian)* ……………………………………………………………..

M, Miss, *(name, first name of parent/legal guardian)* ……………………………………………………………..

I, M, Miss, ……………………………………………………………………..*(name, first name of parent)* hereby declare that my consent below has been given voluntarily and that I have understood all of the above. I undertake to also inform the child's father/mother *(please cross off as appropriate)* of my consent for the participation of our child in the clinical trial. If the child's father/mother *(please cross off as appropriate)* does not agree to affix his/her consent to mine, I undertake to inform the physician in-charge and to withdraw my consent for the participation of my child in the clinical trial. I have also received a lawfully and dated copy of this informed consent form

I agree that my child (name, first name of the child)…………………………………………………….t**akes part of the study named "***Risk-stratified randomized controlled trial in paediatric Crohn's Disease : Methotrexate versus azathioprine or adalimumab for maintaining remission in patients at low or at high risk for aggressive disease course, respectively – a treatment strategy* " , managed by PIBD Net. It has been explained to us by *(name, first name of explaining investigator /sub investigator, phone,)*……………………………………………
………
………………………………………………………………………………………physician in this clinical trial.

- We hereby declare that we agree for our child to participate in the clinical trial as detailed in this document
- Our child has been informed and agreed to take part of this clinical trial.
- We had the opportunity to ask all the questions we had to the physician who explained potential risks and constraints linked to our child participation in this clinical trial.
- We received appropriate answers to all our questions
- We hereby declare that at the time of signing this document, our child is not participating in another clinical trial that involves the use of any investigational product, and that we undertake that our child will not participate in any other clinical that involves the use of an investigational product throughout the course of this trial.
- We declare that our child has a health insurance.
- .We hereby declare that we are free to choose that our child will not participate in the clinical trial, and that we are free to stop our child participation in the trial at any time, and all of this without prejudice to our child's right to receive the standard treatment. Then, we will inform the physician whether data collected prior our decision can be used or not.
- We have been informed that the doctor has the right to take our child out of the study at any time, if needed.
- That in case of completing a questionnaire – we are entitled not to answer all or some of the questions in the questionnaire.
- We are informed that samples collected during this clinical trial will be kept and used for further testing on Crohn's disease. We can decide at any time not to have those samples used by informing our child physician.
- That we are guaranteed confidentiality concerning the identity of the patient and that of the parents/guardians. This confidentiality will be kept by all those concerned with and involved in the clinical trial, and their identity will not be disclosed in any publication.
- That the Medical Institution has arranged for appropriate insurance coverage of the investigators, physicians and medical staff involved in the clinical trial, against claims filed by clinical trial participants and/or third party claims related to the clinical trial, either during the course of the trial or thereafter. This is

For peer review only - http://bmjopen.bmj.com/site/about/guidelines.xhtml

PIBD-SETQuality

without prejudice to our rights under the law.

- That in case of pregnancy during the course of the clinical trial, the girl/woman will be counselled (by the principal investigator) concerning the possible effects on the foetus and the fate of the pregnancy, including the possibility of discontinuing the pregnancy.
- We hereby declare that our below consent has been given voluntarily and that we have understood all of the above mentioned. We also received a lawfully signed and dated copy of this informed consent.
- By signing this consent form, we authorize the sponsor of the clinical trial, the Institutional Helsinki Committee, the auditing entity at the Medical Institute and the Ministry of Health direct access to the patient's medical file, to verify the clinical trial methods and the clinical data. This access to our child medical information will be performed with confidentiality maintained, according to the laws and procedures of maintaining confidentiality.
- We declare that we are informed and give our approval to receive all information related to our child participation in this clinical trial. We know that data will only be used for treatment and follow up cares
- We hereby declare that we know and agree to have the information on our child's participation in the clinical trial provided to his/her attending physician at the HMO/Health care Services with which our child is insured, in case the clinical trial involved the provision of services : performing medical examinations or supplying devices or products or implants. We know that the HMO will not use this information for purposes other than medical treatment and follow up

I agree to have my child participating in the ancillary study (« ADA STEP-up »)

Yes ☐ No ☐ *[please tick]*

**Signature of parents or guardians/representatives of the patient**	**Signature of the child**
Name, First Name :________________<br>Date : Signature :<br><br>Name, First Name :________________<br>Date : Signature :	Name, First Name :________________<br>Date : Signature :

Declaration of the Investigator/Sub-Investigator :This consent was obtained by me after I have explained all the above mentioned to the parents (or guardians) of the clinical trial participant and ensure that all my explanations were understood by them.

**Investigator/Sub-investigator' Signature :**
Name, First Name: ________________________________
Date: Signature :

For peer review only

For peer review only - http://bmjopen.bmj.com/site/about/guidelines.xhtml

PIBD-SETQuality

This is a triplicate document. First / original copy to be kept by the investigator for 15 years, second copy to be given to parents or legal guardians, third copy to be kept in Investigator files (under sealed envelope).

For peer review only

For peer review only - http://bmjopen.bmj.com/site/about/guidelines.xhtml

PIBD-SETQuality

European Commission

Informed consent for Genetic Analyses

Hereby declare that we agree for genetic examinations of our child to study genes involved in tolerance / non tolerance of the drugs by molecular ("omic") analyses and analyses of drug efficacy in Crohn disease's patients.

Hereby declare that we agree that all recorded data collected during this trial including genetic data can be processed by the sponsor or acting as sponsor. I understand that, as stipulated in the General Data Protection Regulation, I can access, modify, erase or ask for a copy of my child's personal data and my personal data at any time, by asking to the investigator who will contact the sponsor.

We can decide not to participate anymore in the genetic part of the trial by informing our doctor who will inform the sponsor.

Yes ☐ No ☐ *[please tick]*

Hereby declare that we agree that all biological samples collected during this trial can be used for future genetic research on Crohn's disease.

Yes ☐ No ☐ *[please tick]*

Parents/guardians Signature:

Name, First name:

Date : Signature :

Name, First name:

Date : Signature :

Investigator Signature:

Name, First name:

Date : Signature :

For peer review only

For peer review only - http://bmjopen.bmj.com/site/about/guidelines.xhtml

Reporting checklist for protocol of a clinical trial.

Based on the SPIRIT guidelines.

Instructions to authors

Complete this checklist by entering the page numbers from your manuscript where readers will find each of the items listed below.

Your article may not currently address all the items on the checklist. Please modify your text to include the missing information. If you are certain that an item does not apply, please write "n/a" and provide a short explanation.

Upload your completed checklist as an extra file when you submit to a journal.

In your methods section, say that you used the SPIRITreporting guidelines, and cite them as:

Chan A-W, Tetzlaff JM, Altman DG, Laupacis A, Gøtzsche PC, Krleža-Jerić K, Hróbjartsson A, Mann H, Dickersin K, Berlin J, Doré C, Parulekar W, Summerskill W, Groves T, Schulz K, Sox H, Rockhold FW, Rennie D, Moher D. SPIRIT 2013 Statement: Defining standard protocol items for clinical trials. Ann Intern Med. 2013;158(3):200-207

		Reporting Item	Page Number
**Administrative information**			
Title	#1	Descriptive title identifying the study design, population, interventions, and, if applicable, trial acronym	1
Trial registration	#2a	Trial identifier and registry name. If not yet registered, name of intended registry	4
Trial registration: data set	#2b	All items from the World Health Organization Trial Registration Data Set	Throughout manuscript
Protocol version	#3	Date and version identifier	21
Funding	#4	Sources and types of financial, material, and other support	30
Roles and responsibilities:	#5a	Names, affiliations, and roles of protocol contributors	1,2,30

For peer review only

contributorship			
Roles and responsibilities: sponsor contact information	#5b	Name and contact information for the trial sponsor	29
Roles and responsibilities: sponsor and funder	#5c	Role of study sponsor and funders, if any, in study design; collection, management, analysis, and interpretation of data; writing of the report; and the decision to submit the report for publication, including whether they will have ultimate authority over any of these activities	30
Roles and responsibilities: committees	#5d	Composition, roles, and responsibilities of the coordinating centre, steering committee, endpoint adjudication committee, data management team, and other individuals or groups overseeing the trial, if applicable (see Item 21a for data monitoring committee)	23,30
**Introduction**			
Background and rationale	#6a	Description of research question and justification for undertaking the trial, including summary of relevant studies (published and unpublished) examining benefits and harms for each intervention	6-8
Background and rationale: choice of comparators	#6b	Explanation for choice of comparators	6-7
Objectives	#7	Specific objectives or hypotheses	8-10
Trial design	#8	Description of trial design including type of trial (eg, parallel group, crossover, factorial, single group), allocation ratio, and framework (eg, superiority, equivalence, non-inferiority, exploratory)	
**Methods: Participants, interventions, and outcomes**			

For peer review only

Study setting	#9	Description of study settings (eg, community clinic, academic hospital) and list of countries where data will be collected. Reference to where list of study sites can be obtained	Supplemental table 1
Eligibility criteria	#10	Inclusion and exclusion criteria for participants. If applicable, eligibility criteria for study centres and individuals who will perform the interventions (eg, surgeons, psychotherapists)	10-12
Interventions: description	#11a	Interventions for each group with sufficient detail to allow replication, including how and when they will be administered	15-16
Interventions: modifications	#11b	Criteria for discontinuing or modifying allocated interventions for a given trial participant (eg, drug dose change in response to harms, participant request, or improving / worsening disease)	18, 19,22
Interventions: adherance	#11c	Strategies to improve adherence to intervention protocols, and any procedures for monitoring adherence (eg, drug tablet return; laboratory tests)	19-21
Interventions: concomitant care	#11d	Relevant concomitant care and interventions that are permitted or prohibited during the trial	13,23
Outcomes	#12	Primary, secondary, and other outcomes, including the specific measurement variable (eg, systolic blood pressure), analysis metric (eg, change from baseline, final value, time to event), method of aggregation (eg, median, proportion), and time point for each outcome. Explanation of the clinical relevance of chosen efficacy and harm outcomes is strongly recommended	9-10
Participant timeline	#13	Time schedule of enrolment, interventions (including any run-ins and washouts), assessments, and visits for participants. A schematic diagram is highly recommended (see Figure)	See figure 1<br>8,12,15,16-19
Sample size	#14	Estimated number of participants needed to achieve study objectives and how it was determined, including clinical and statistical assumptions	27

For peer review only

		supporting any sample size calculations	
Recruitment	#15	Strategies for achieving adequate participant enrolment to reach target sample size	Not listed
**Methods: Assignment of interventions (for controlled trials)**			
Allocation: sequence generation	#16a	Method of generating the allocation sequence (eg, computer-generated random numbers), and list of any factors for stratification. To reduce predictability of a random sequence, details of any planned restriction (eg, blocking) should be provided in a separate document that is unavailable to those who enrol participants or assign interventions	15,16,22
Allocation concealment mechanism	#16b	Mechanism of implementing the allocation sequence (eg, central telephone; sequentially numbered, opaque, sealed envelopes), describing any steps to conceal the sequence until interventions are assigned	22
Allocation: implementation	#16c	Who will generate the allocation sequence, who will enrol participants, and who will assign participants to interventions	15
Blinding (masking)	#17a	Who will be blinded after assignment to interventions (eg, trial participants, care providers, outcome assessors, data analysts), and how	22
Blinding (masking): emergency unblinding	#17b	If blinded, circumstances under which unblinding is permissible, and procedure for revealing a participant's allocated intervention during the trial	N/A
**Methods: Data collection, management, and analysis**			
Data collection plan	#18a	Plans for assessment and collection of outcome, baseline, and other trial data, including any related processes to promote data quality (eg, duplicate	24-25

For peer review only

		measurements, training of assessors) and a description of study instruments (eg, questionnaires, laboratory tests) along with their reliability and validity, if known. Reference to where data collection forms can be found, if not in the protocol	
Data collection plan: retention	#18b	Plans to promote participant retention and complete follow-up, including list of any outcome data to be collected for participants who discontinue or deviate from intervention protocols	Not listed
Data management	#19	Plans for data entry, coding, security, and storage, including any related processes to promote data quality (eg, double data entry; range checks for data values). Reference to where details of data management procedures can be found, if not in the protocol	25
Statistics: outcomes	#20a	Statistical methods for analysing primary and secondary outcomes. Reference to where other details of the statistical analysis plan can be found, if not in the protocol	25-26
Statistics: additional analyses	#20b	Methods for any additional analyses (eg, subgroup and adjusted analyses)	26-27
Statistics: analysis population and missing data	#20c	Definition of analysis population relating to protocol non-adherence (eg, as randomised analysis), and any statistical methods to handle missing data (eg, multiple imputation)	26
**Methods: Monitoring**			
Data monitoring: formal committee	#21a	Composition of data monitoring committee (DMC); summary of its role and reporting structure; statement of whether it is independent from the sponsor and competing interests; and reference to where further details about its charter can be found, if not in the protocol. Alternatively, an explanation of why a DMC is not needed	23
Data monitoring:	#21b	Description of any interim analyses and stopping	23

For peer review only

interim analysis		guidelines, including who will have access to these interim results and make the final decision to terminate the trial	
Harms	#22	Plans for collecting, assessing, reporting, and managing solicited and spontaneously reported adverse events and other unintended effects of trial interventions or trial conduct	23
Auditing	#23	Frequency and procedures for auditing trial conduct, if any, and whether the process will be independent from investigators and the sponsor	24
**Ethics and dissemination**			
Research ethics approval	#24	Plans for seeking research ethics committee / institutional review board (REC / IRB) approval	4
Protocol amendments	#25	Plans for communicating important protocol modifications (eg, changes to eligibility criteria, outcomes, analyses) to relevant parties (eg, investigators, REC / IRBs, trial participants, trial registries, journals, regulators)	22
Consent or assent	#26a	Who will obtain informed consent or assent from potential trial participants or authorised surrogates, and how (see Item 32)	29-30
Consent or assent: ancillary studies	#26b	Additional consent provisions for collection and use of participant data and biological specimens in ancillary studies, if applicable	No additional consent see page 30
Confidentiality	#27	How personal information about potential and enrolled participants will be collected, shared, and maintained in order to protect confidentiality before, during, and after the trial	24
Declaration of interests	#28	Financial and other competing interests for principal investigators for the overall trial and each study site	30
Data access	#29	Statement of who will have access to the final trial dataset, and disclosure of contractual agreements that limit such access for investigators	Not provided

For peer review only

Ancillary and post trial care	#30	Provisions, if any, for ancillary and post-trial care, and for compensation to those who suffer harm from trial participation	N/A
Dissemination policy: trial results	#31a	Plans for investigators and sponsor to communicate trial results to participants, healthcare professionals, the public, and other relevant groups (eg, via publication, reporting in results databases, or other data sharing arrangements), including any publication restrictions	29
Dissemination policy: authorship	#31b	Authorship eligibility guidelines and any intended use of professional writers	29
Dissemination policy: reproducible research	#31c	Plans, if any, for granting public access to the full protocol, participant-level dataset, and statistical code	Not planned
**Appendices**			
Informed consent materials	#32	Model consent form and other related documentation given to participants and authorised surrogates	Appendix 1
Biological specimens	#33	Plans for collection, laboratory evaluation, and storage of biological specimens for genetic or molecular analysis in the current trial and for future use in ancillary studies, if applicable	Not provided

None The SPIRIT checklist is distributed under the terms of the Creative Commons Attribution License CC-BY-ND 3.0. This checklist can be completed online using https://www.goodreports.org/, a tool made by the EQUATOR Network in collaboration with Penelope.ai

For peer review only

BMJ Open

PROTOCOL FOR A MULTI-NATIONAL RISK-STRATIFIED RANDOMISED CONTROLLED TRIAL IN PAEDIATRIC CROHN'S DISEASE: METHOTREXATE VERSUS AZATHIOPRINE OR ADALIMUMAB FOR MAINTAINING REMISSION IN PATIENTS AT LOW OR HIGH RISK FOR AGGRESSIVE DISEASE COURSE

Journal:	*BMJ Open*
Manuscript ID	bmjopen-2019-034892.R2
Article Type:	Protocol
Date Submitted by the Author:	21-Apr-2020
Complete List of Authors:	Harris, Rachel; Royal Hospital for Children Glasgow<br>Aloi, Marina; Sapienza University of Rome, Paediatric Gastroenterology and Liver Unit<br>de Ridder, Lissy; Erasmus MC / Sophia Childrens Hospital, Paediatrics<br>Croft, Nicholas; Barts and The London School of Medicine and Dentistry<br>Koletzko, Sibylle; Dr. v. Hauner Children's Hospital, Pediatric Gastroenterology and Hepatology; Collegium Medicum University of Warmia and Mazury, Department of Pediatrics<br>Levine, Arie; Tel Aviv University, Edith Wolfson Medical Center,<br>Turner, Dan; Hebrew University of Jerusalem<br>Veereman, Gigi; UZBrussels-VUB, Pediatric GI; Free University Brussels, University Hospital, Veereman<br>Neyt , Mattias ; ME-TA Medical Evaluation and Technology Assessment<br>Bigot, Laetitia; Hôpital universitaire Necker-Enfants malades, PIBD-Net<br>Ruemmele, Frank; Hôpital universitaire Necker-Enfants malades, Service de Gastroentérologie pédiatrique; Université Paris Descartes<br>russell, richard; Royal Hospital for Children Glasgow, ; RHC Glasgow
**Primary Subject Heading**:	Gastroenterology and hepatology
Secondary Subject Heading:	Paediatrics, Pharmacology and therapeutics, Patient-centred medicine
Keywords:	Inflammatory bowel disease < GASTROENTEROLOGY, Paediatric gastroenterology < GASTROENTEROLOGY, Clinical trials < THERAPEUTICS

SCHOLARONE™ Manuscripts

I, the Submitting Author has the right to grant and does grant on behalf of all authors of the Work (as defined in the below author licence), an exclusive licence and/or a non-exclusive licence for contributions from authors who are: i) UK Crown employees; ii) where BMJ has agreed a CC-BY licence shall apply, and/or iii) in accordance with the terms applicable for US Federal Government officers or employees acting as part of their official duties; on a worldwide, perpetual, irrevocable, royalty-free basis to BMJ Publishing Group Ltd ("BMJ") its licensees and where the relevant Journal is co-owned by BMJ to the co-owners of the Journal, to publish the Work in this journal and any other BMJ products and to exploit all rights, as set out in our licence.

The Submitting Author accepts and understands that any supply made under these terms is made by BMJ to the Submitting Author unless you are acting as an employee on behalf of your employer or a postgraduate student of an affiliated institution which is paying any applicable article publishing charge ("APC") for Open Access articles. Where the Submitting Author wishes to make the Work available on an Open Access basis (and intends to pay the relevant APC), the terms of reuse of such Open Access shall be governed by a Creative Commons licence – details of these licences and which Creative Commons licence will apply to this Work are set out in our licence referred to above.

Other than as permitted in any relevant BMJ Author's Self Archiving Policies, I confirm this Work has not been accepted for publication elsewhere, is not being considered for publication elsewhere and does not duplicate material already published. I confirm all authors consent to publication of this Work and authorise the granting of this licence.

For peer review only

PROTOCOL FOR A MULTI-NATIONAL RISK-STRATIFIED RANDOMISED CONTROLLED TRIAL IN PAEDIATRIC CROHN'S DISEASE: METHOTREXATE VERSUS AZATHIOPRINE OR ADALIMUMAB FOR MAINTAINING REMISSION IN PATIENTS AT LOW OR HIGH RISK FOR AGGRESSIVE DISEASE COURSE

Rachel E. Harris[1]; Marina Aloi[2]; Lissy de Ridder[3]; Nicholas M Croft[4]; Sibylle Koletzko[5,6]; Arie Levine[7]; Dan Turner[8]; Gigi Veereman [9]; Mattias Neyt[10] Laetitia Bigot[11]; Frank M. Ruemmele[12,13*]; Richard K. Russell[1*]; on behalf of the PIBD SETQuality consortium and PIBDnet

1. Department of Paediatric Gastroenterology, Hepatology and Nutrition, Royal Hospital for Children, Glasgow, UK
2. Paediatric Gastroenterology and Liver Unit, Department of Paediatrics, Sapienza University of Rome, Rome, Italy
3. Erasmus MC/Sophia Children's Hospital, Rotterdam, The Netherlands
4. Barts and the London School of Medicine, Queen Mary University of London, London, UK.
5. Department of Pediatrics, Dr. von Hauner Children's Hospital, University Hospital, LMU Munich, Munich, Germany
6. Department of Pediatrics, Gastroenterology and Nutrition, School of Medicine Collegium Medicum University of Warmia and Mazury, Olsztyn, Poland
7. Paediatric Gastroenterology and Nutrition Unit, Tel Aviv University, Edith Wolfson Medical Center, Holon, Israel
8. Institute of Paediatric Gastroenterology, Shaare Zedek Medical Center, The Hebrew University of Jerusalem, Jerusalem, Israel
9. Department of Paediatric Gastroenterology and Nutrition, UZ Brussel - Vrije Universiteit Brussel, Brussels, Belgium

For peer review only

10. ME-TA Medical Evaluation and Technology Assessment, Merendree, Belgium

11. Pediatric Inflammatory Bowel Disease network (PIBD-net), Hôpital Necker Enfants Malades, Paris, France

12. Université Paris Descartes, Sorbonne Paris Cité, Paris, France

13. Assistance Publique-Hôpitaux de Paris, Hôpital Necker-Enfants Malades, Service de Gastroentérologie pédiatrique, Paris, France

* FMR and RKR share senior authorship

Corresponding Author:

Professor Richard K. Russell

Department of Paediatric Gastroenterology, Hepatology and Nutrition

Royal Hospital for Children

1345 Govan Road

Glasgow

G51 4TF

UNITED KINGDOM

Telephone: 0044 141 451 6543

Email: richardrussell@nhs.net

Key Words: Paediatric Gastroenterology, Inflammatory Bowel Disease, Crohn's Disease, Immunomodulators

For peer review only

ABSTRACT

Introduction

Immunomodulators such as thiopurines (azathioprine (AZA)/6-mercaptopurine (6MP)), methotrexate (MTX) and biologics such as adalimumab (ADA) are well established for maintenance of remission within paediatric Crohn's disease (CD). It remains unclear however which maintenance medication should be used first-line in specific patient groups.

Aims

To compare the efficacy of maintenance therapies in newly diagnosed CD based upon stratification into high and low risk groups for severe CD evolution; MTX versus AZA/6MP in low-risk and MTX versus ADA in high-risk patients. Primary end point: sustained remission at 12 months (weighted paediatric Crohn's disease activity index ≤12.5 and C-reactive protein ≤1.5-fold upper limit) without relapse or ongoing requirement for EEN/steroids 12 weeks after treatment initiation.

Methods and Analysis

REDUCE-RISK in CD is an international multicentre open-label prospective randomised controlled trial funded by EU within the Horizon2020 framework (grant number 668023). Eligible patients (aged 6-17 years, new-onset disease receiving steroids or EEN for induction of remission for luminal +/- perianal CD are stratified into low and high-risk groups based upon phenotype and response to induction therapy. Participants are randomised to one of two treatment arms within their risk

For peer review only

group: low-risk patients to weekly subcutaneous MTX or daily oral AZA/6MP, and high-risk patients to weekly subcutaneous MTX or fortnightly ADA. Patients are followed up for 12 months at pre-specified intervals. Electronic case report forms are completed prospectively. The study aims to recruit 312 participants (176 low-risk; 136 high-risk).

Ethics and Dissemination

ClinicalTrials.gov Identifier: (NCT02852694), authorisation and approval from local ethics committees have been obtained prior to recruitment. Individual informed consent will be obtained prior to participation in the study. Results will be published in a peer-reviewed journal with open access.

Registration Details

NCT02852694; pre-results.

Strengths and limitations

- This is the 1st international prospective RCT comparing 3 different i medications for maintenance of remission in newly diagnosed CD
- This study may better define the most appropriate first-line immunomodulators based upon a risk stratification protocol. .
- Therapeutic efficacy will be supported by drug levels, pharmacogenomics and microbiome analysis as secondary outcomes.
- Inability to blind participants or treating physicians serves as a limitation to this study.

For peer review only

- Blinding of an alternative clinician to assess disease activity during study visits may prove practically difficult in smaller centres.

For peer review only

INTRODUCTION

Crohn's disease (CD) the most common form of inflammatory bowel disease (IBD) in children is a chronic disorder with the potential to affect the whole gastrointestinal tract. The aim of CD treatment is to control active inflammation and achieve bowel healing. Chronic and uncontrolled CD results in poor outcomes for patients, including reduced quality of life, recurrent hospitalisation and potential need for surgical intervention.[1] Treatments for CD are categorised into those which induce remission (such as steroids[1,2] or exclusive enteral nutrition (EEN)[1,3] and those which maintain remission. Immunomodulators are a mainstay of maintenance treatment in IBD, with the efficacy of thiopurines (e.g. azathioprine (AZA) and 6-mercaptopurine (6MP))[4,5,6]

and methotrexate (MTX)[7,8,9,10] well established. Anti-tumour necrosis factor (anti-TNF) therapies (infliximab[11,12] and adalimumab (ADA)[13,14]) including their biosimilars were used in those patients refractory to “traditional” induction or maintenance treatment. More recently in clinical practice patients deemed as high risk have been treated with a biologic without the need for prior use of an immunomodulator.

Due to a lack of treatment strategy trials within the paediatric IBD (PIBD) population however, it remains unclear which of the aforementioned maintenance therapies should be used first-line in individual patients. Randomised controlled trials comparing the use of MTX with thiopurines for maintenance of remission failed to show a significant difference in efficacy between the two.[15,16, 17] A Cochrane review in adults with quiescent CD highlighted the lack of adequately powered trials necessary in order to determine the efficacy and safety of thiopurines compared to other maintenance therapies[4, 10]. The RISK study (observational, non-randomised study) demonstrated improved clinical and growth-based outcomes at 1 year with anti-TNF monotherapy in comparison with immunomodulators; however further investigation into which specific patients are most likely to benefit from these therapies is still required.[18] There is a clear disparity between North America and Europe in terms of which form of immunosuppression is used initially with both concerns about efficacy and safety lying behind these differences, thus there is an urgent need for a head to head study in children to help objectively inform the primary choice of immunosuppression.

Stratifying patients by risk for complex or severe CD may allow pre-emptive direction of maintenance strategy and potentially an early reduction in disease burden with subsequent improvement in long-term outcomes. The adult IBD Ahead initiative highlighted young age at diagnosis as a risk factor for severity of CD evolution[19]; all

For peer review only

patients diagnosed within paediatric services would therefore be considered ‘high risk’. Paediatric consensus guidelines suggest that paediatric CD patients at ‘high risk for poor outcome’ should receive early therapy optimisation to modify progression of their disease.[1] The guidelines list specific features which may be considered predictive for poor outcome in paediatric CD (see Table 1).[1] Patients deemed at high risk for complex disease or poor outcome may benefit from a ‘Top-down’ approach as the TISKids (a randomised controlled trial from disease diagnosis) aims to investigate[20].

Therefore the PIBDnet consortium recognised the urgent need to investigate the efficacy and safety of immunomodulators and to investigate whether a top-down approach was superior to a traditional ‘step-up’ for paediatric patients deemed at high risk for rapidly complicated disease course. REDUCE-RISK in CD is a randomised controlled trial (RCT) which aims to compare the effectiveness of immunomodulators for maintenance of remission in newly diagnosed CD based upon risk stratification specifically, the effectiveness of MTX versus AZA/6MP for maintenance of remission who are low risk for rapidly progressive disease and the effectiveness of MTX versus ADA in a high risk group.

METHODS AND ANALYSIS

Study Design

We designed an international multicentre open-label prospective RCT with 4 treatment arms as shown in Figure 1. Following screening and consent, eligible patients are stratified into low and high-risk groups based upon phenotype and

For peer review only

disease response to induction therapy (Table 1). Patients are then randomised to one of two arms within their risk group, with low risk patients receiving either weekly subcutaneous MTX or daily oral AZA/6MP and high-risk patients receiving either weekly subcutaneous MTX or fortnightly subcutaneous ADA.

Study End Points

Patients are followed up for 12 months post randomisation. The primary end point of the study is sustained steroid or EEN-free remission at 12 months, defined as weighted Paediatric Crohn's Disease Activity Index (wPCDAI) ≤12.5 and C-reactive protein (CRP) ≤1.5-fold upper limit without a relapse or need for EEN/steroids since week 12.

Secondary end points include comparison of time to first relapse, remission at 12 weeks, growth, adverse events, health related quality of life and patient reported outcomes between the two treatment arms within each risk group but also between low and high risk MTX treated patients (a full list of secondary endpoints can be found in box 1). The TUMMY CD (Patient related outcome measure) was originally included as a secondary end point but has been withdrawn as the original timetable of development and validation of the score has not been met so it was not ready in time to be included. The study also aimed to evaluate clinical predictors for response, including genomic and serological markers and results of drug monitoring (MTX and ADA concentrations) metabolites (6-thioguanine (6-TG) and 6-methylmercaptopurine (6-MMP) in AZA/6MP) and anti-drug antibodies (ADA) in relation to adherence,

For peer review only

toxicity and response. The ancillary study additionally aimed to evaluate the efficacy of ADA in patients treated from inclusion (Top-down) versus patients switched to ADA due to immunomodulator failure (Step-up). Further outcome measures are detailed in Box 1.

Box 1: Study endpoints

Primary Endpoint

- Sustained steroid/EEN-free remission at month 12, where sustained remission is defined as wPCDAI ≤12.5 and CRP ≤1.5 times the upper limit without a relapse or need for EEN/steroids since week 12.

Secondary Endpoints*:*

Comparing the following within 1) the two treatment arms per risk group; 2) methotrexate treatment between high and low risk groups; and 3) TOP-Down adalimumab (high risk group) versus STEP-Up adalimumab (ancillary study):

- Rate of clinical remission at month 12 (physician global assessment (PGA), wPCDAI, paediatric Crohn's disease activity index (PCDAI))
- Relapse free remission with normal CRP at month 12
- Relapse free remission with normal CRP and faecal calprotectin <300 at month 12
- Remission at week 12 (measured by wPCDAI</=12.5 and normal CRP and being off steroids/exclusive enteral nutrition)
- Time to first relapse after week 12
- Predictive value of faecal calprotectin values at visits 1, 2, 4 and 6 (respectively at month 0, 2, 6 and 12)
- Dropout rates
- Adverse drug event rate including pharmacogenomics for toxicity and response to therapy
- Height velocity and z-score at baseline and 52 weeks
- Quality of life as measured by the IMPACT 3 questionnaire completed at each study visit
- Health economic evaluation at all visits (forms EQ-5D-Y proxy 1, EQ-5D-Y and EQ-5D-5L, WPAI:CD Caregiver, School Attendance start of the research and follow up visits)

Eligibility Criteria and Recruitment

Full eligibility criteria for the study are listed in Box 2. Patients are eligible if aged 6-17 years with new-onset (<6 months) treatment naïve luminally active and/or perianal fistulising CD diagnosed as per revised Porto criteria[21] receiving steroids or EEN for induction of remission with wPCDAI >40 or CRP >2 times upper limit of normal at diagnosis. Eligible definitions of disease behaviour were derived from the Paris classification. [22] Informed consent from must be obtained prior to participation in the study. Patients are excluded in cases of previous use of IBD related medications, pregnancy or refusal to use contraceptives; disease requiring surgery, contraindications to study medication, exposure to live vaccine within 3 weeks, oral anticoagulant or anti-malarial use, current or previous malignancy, significant infection or significant comorbidity.

The planned start date for the study is 01.2017 with planned end 06/2022.

For peer review only

Box 2: Eligibility criteria

Inclusion Criteria

- Patients aged 6-17 years with new-onset (<6 months) treatment naïve active luminal and/or perianal fistulising Crohn's disease diagnosed using established criteria[21]requiring steroids or EEN for induction of remission
- wPCDAI >40 or CRP >2 times upper limit of normal at diagnosis
- Luminal active Crohn's disease (B1) with or without B2 and/or B3 disease behaviour as per Paris classification [22]
- Signed informed consent

Exclusion Criteria

- wPCDAI <42.5 at diagnosis, except where CRP >2 times upper normal limit
- Lack of induction therapy with steroids or EEN
- Previous therapy with any IBD-related medication other than induction therapy as detailed within this protocol with the exception of 5-aminosalicylic acid (5ASA) preparations
- Pregnancy or refusal to use contraceptives during the study period in pubertal patients unless absolute abstinence is confirmed at each study visit
- Lactating mothers
- Perianal fistulising disease requiring surgical therapy
- Patients homozygous for thiopurine methyltransferase (TPMT) mutations or those with TPMT activity <6 nmol/h/ml erythrocytes or <9nmol 6MTG/g Hb/h, unless they qualify as high-risk patients
- Evidence of un-drained and un-controlled abscess/phlegmon
- Contraindication to any drugs used in the trial (including intolerance/hypersensitivity or allergy to study drugs (thiopurines, methotrexate or adalimumab))
- Current or previous malignancy
- Serious comorbidities (e.g. renal insufficiency, hepatitis, respiratory insufficiency) which may interfere with drug therapy or interpretation of outcome parameters or will make it unlikely that the patient will complete the trial.
- Infection with mycobacterium tuberculosis, hepatitis B or C, human immunodeficiency virus (HIV)
- Moderate to severe heart failure (New York Heart Association class III/IV)
- Oral anticoagulant therapy, anti-malarial therapy
- Live vaccine exposure (including yellow fever) less than 3 weeks prior to inclusion

<u>Screening Visit (Visit 0)</u>

The screening visit allows for assessment of eligibility for inclusion in the study, evaluation of the patient's response to induction therapy if already commenced,

commencement of induction therapy where not commenced, and acquisition of consent and assent.

Induction Therapy

All enrolled patients receive either corticosteroids or exclusive enteral nutrition (EEN) as induction as determined by the clinical team and the patient/caregiver. For EEN any balanced formula (polymeric or elemental) administered orally or via nasogastric tube is permitted and should be prescribed for 6-8 weeks. Tapering of steroids is at the discretion of the prescribing clinician. Adaptation of induction therapy (e.g. dose increase of steroids or return to EEN) or crossover from one induction therapy to the other is permitted in order to achieve remission, however patients must have discontinued their induction therapy by week 12. If induction therapy is not discontinued by week 12 the patient is considered a treatment failure, with the protocol for this detailed below.

<u>Inclusion Visit and Risk Group Allocation (week 5 +/- 3 weeks; visit 1)</u>

In order to incorporate response to initial induction therapy within the risk stratification criteria, inclusion and risk group allocation is performed at week 5 +/- 3 weeks of induction therapy. Data from the screening visit is reviewed with ineligible patients excluded and patients are then stratified into the high or low risk group (Table 1) based upon the ECCO/ESPGHAN consensus guidelines[1]. Patients with

For peer review only

perianal fistulising disease at diagnosis are auto-allocated to the high-risk group regardless of other factors at inclusion visit. All other patients are allocated to the low risk group. Patients with low thiopurine methyltransferase (TPMT) activity or homozygous mutations are excluded should they be categorised as low risk.

**DEFINING HIGH RISK CROHN'S DISEASE PATIENTS**	
**ECCO/ESPGHAN CONSENSUS GUIDELINES**	**MODIFIED STUDY CRITERIA**
Severe perianal disease	Complex perianal fistulising disease phenotype
Extensive (pan-enteric) disease; deep colonic ulcers on endoscopy	Panenteric disease phenotype (defined as L3 with L4b as per Paris classification[23] or L3 with deep ulcers in the duodenum, stomach or oesophagus not related to non-steroidal anti-inflammatory medications or Helicobacter pylori)
	Overall cumulative disease extent of >/=60cm
Stricturing and penetrating disease at onset	B2, B3 or B2B3 disease behaviour [20]
Marked growth retardation >-2.5 height Z scores	Severe growth impairment (height z-score <-2 or crossing >/= 2 centiles) likely related to Crohn's disease
Persistent severe disease despite adequate induction therapy	Hypoalbuminemia (<30g/L), elevated CRP (at least 2 times upper limit of normal range), or wPCDAI>12.5 despite at least 3 weeks of optimized induction therapy with steroids or EEN
Severe osteoporosis	Not included

Table 1 – Definition of high-risk patients based upon ECCO/ESPGHAN consensus guidelines (ECCO – European Crohn's and Colitis Organisation; ESPGHAN – European Society for Paediatric Gastroenterology, Hepatology and Nutrition; CRP – C-reactive protein; wPCDAI – weighted Paediatric Crohn's Disease Activity Index; EEN – exclusive enteral nutrition)

For peer review only - http://bmjopen.bmj.com/site/about/guidelines.xhtml

Randomisation and Treatment Allocation

Randomisation is undertaken following allocation to high or low risk group at week 5 +/- 3 weeks. This process utilises an integrated module within the electronic case report form (CRF) system. Within both the high and low risk groups patients are 1:1 randomised to MTX versus ADA or AZA/6MP respectively in blocks of four stratified by EEN or steroid induction therapy. Code for randomisation is prepared and held by the central coordinating site and site co-ordinators are then informed of the results. Immunomodulator or biologic therapy should be commenced within 2 weeks of randomisation as per the protocol outlined in Table 2.

AZA/6MP and MTX are prescribed and dispensed according to local guidelines. ADA (Humira ®) is provided by AbbVie. Co-interventions are prohibited.

For peer review only

	Therapy	Route	Dose	Notes
**LOW RISK PROTOCOL**	Methotrexate	SC	15mg/m^2 body surface area weekly (max dose 25mg)	Ondansetron 4-8mg orally 1 hour pre injection and folic acid 15mg (5mg in patients <20kg) 3 days post injection are recommended for all patients
	**VERSUS**			
	Azathioprine	PO	2.5mg/kg (rounded down to nearest 12.5mg)	Half calculated dose for TPMT heterozygotes/activity 6-9nmol/h/ml
	**OR**			
	6-Mercaptopurine	PO	1.5mg/kg (rounded down to nearest 12.5mg)	Half calculated dose for TPMT heterozygotes/activity 6-9nmol/h/ml
**HIGH RISK PROTOCOL**	Methotrexate	SC	15mg/m^2 body surface area weekly (max dose 25mg)	Ondansetron 4-8mg orally 1 hour pre injection and folic acid 15mg (5mg in patients <20kg) 3 days post injection are recommended for all patients
	**VERSUS**			
	Adalimumab<br><br>(Humira ®)	SC	160mg then 80mg after 2 weeks then 40mg every 2 weeks thereafter (patients >35kg)<br><br>120mg then 80mg after 2 weeks then 40mg every 2 weeks thereafter (patients 25-35kg)<br><br>80mg then 40mg after 2 weeks and 20mg every 2 weeks thereafter (patients <25kg)	

Table 2: Medication protocol for low and high-risk patients following randomisation

(TPMT – thiopurine methytransferase)

For peer review only

Follow Up Visits (Visit 2, 3, 4, 5 and 6)

Patients are followed up at pre-specified intervals (Figure 1) with a window of +/- 2 weeks. A telephone call is undertaken at week 4 following initiation of induction in order to support patient compliance with induction regime and advise weaning where appropriate. Data as described in Box 3 are collected at each consultation. Patients' compliance with therapy is determined at each face-to-face follow up visit by pill and vial counts plus by patients' reporting.

Box 3: Standard requirements for each study visit

- An explicit history of illness since last visit, including review of symptoms, medications (including compliance check) and adverse events.
- Physical examination
- wPCDAI, PGA and PCDAI scoring
- Anthropometrics (height measured using a calibrated wall mounted stadiometer)
- Blood tests
  - White blood cells
  - Absolute neutrophil count
  - Haemoglobin
  - Haematocrit
  - Platelet count
  - Erythrocyte sedimentation rate (ESR)
  - C-reactive protein (CRP)
  - Amylase
  - Albumin
  - Aspartate transaminase (AST)
  - Alanine transaminase (ALT)
  - Conjugated bilirubin
  - Gamma glutamyl transferase (GGT)
- Stool samples for faecal calprotectin and microbiome analysis
- Health economic parameters (EQ-5D-Y proxy 1; EQ-5D-Y; EQ-5D-5L; WPAI:CD; school attendance questionnaire)
- Quality of life evaluation (IMPACT 3)
- Urine human chorionic gonadotropin (hCG) in all female patients of child-bearing potential
- Confirmation of contraception use or of absolute abstinence in all patients

Remission is defined as wPCDAI</=12.5, normal CRP (</= 1.5 times upper normal range) and being free of steroids or EEN. Once remission is achieved and induction therapy is discontinued, a patient is considered to be failing treatment or experiencing a relapse in the following circumstances:

- wPCDAI >40
- CRP >2 times upper normal limit in the absence of any clear infectious process
- wPCDAI >12.5 but <40 and/or CRP >1.5 times but <2 times over upper normal limit at 2 consecutive visits within 2-8 weeks
- Development of CD related complications e.g. fistulisation
- Requirement for additional CD-specific medication/surgery since last study visit

A patient will also be considered a treatment failure should induction therapy be continued at week 12. In addition, the treating clinician may escalate treatment at any time point independent of wPCDAI score if it is felt that the patient is experiencing a relapse.

For peer review only

Dose Optimisation and Therapeutic Drug Monitoring

Drug monitoring is undertaken as detailed below. In addition to this, samples for drug monitoring should be collected at the time of medication cessation in the event of drug discontinuance due to adverse effect or loss of response. Potential adaptations to therapies which may be made at specific follow up visits are detailed in Box 4.

Box 4 – Potential adaptations to therapies at follow up visits

Month 2 (Visit 2)

- Failure to discontinue induction therapy by week 12
    - Offer switch to the ancillary study (ADA STEP-up) to those prescribed MTX or AZA/6MP, or an increase in dose frequency to weekly in those prescribed ADA
    - Alternatively, the patient may leave the study and receive therapies as per the discretion of the treating clinician.

Months 4, 6, 9 and 12 (Visits 3, 4, 5 and 6)

- Thiopurine non response
    - Protocol as per metabolite levels (detailed in Table 3)
- Thiopurine intolerance (except pancreatitis)
    - Switch to alternate thiopurine (AZA to 6MP or vice versa) or split dose to provide twice daily (BD) dosing
- Thiopurine failure (any exacerbation despite dose optimisation/pancreatitis/cytopaenia)
    - Offer switch to ancillary study (ADA STEP-up) or exit study
- MTX intolerance or failure (any exacerbation or elevation of liver enzymes as detailed below)
    - Offer switch to ancillary study (ADA STEP-up) or exit study
- ADA failure (any exacerbation)
    - Increase frequency to weekly dosing

Azathioprine

RESULT	ACTION
6-TG <150	Consider non-compliance; repeat sample at subsequent visit and increase dose if low 6-TG confirmed (+25mg or +12.5mg if dose <50mg)
6-TG 150-800	No adaptation
6-TG >800	Decrease dose if repeat sample at subsequent visit confirms high 6-TG (-25mg or -12.5mg if dose <50mg)
6-MMP >8000 or signs of hepatotoxicity	Stop medication – switch to ancillary study<br><br>Erythrocyte lysate sample frozen at -80C and shipped to central lab at end of study for thiopurine nucleotides

Table 3 – Azathioprine dose adjustments based upon metabolite levels

TPMT genotype or phenotype at screening determines the initial dose of AZA/6MP; and measurement of thiopurine metabolites (6-thioguanine (6-TG) and 6-methylmercaptopurine (6-MMP)) at visit 2 determines requirement for subsequent dose adjustment performed according to the recommendations in Table 3. Where possible thiopurine metabolites are measured locally; central lab measurements are provided for centres where this is unavailable.

At visit 2 a urine sample for TPMT metabolite determination and an erythrocyte lysate sample for quantification of Thiopurine Nucleotides by Liquid Chromatography-Tandem Mass Spectrometry should be frozen at -80°C and shipped on dried ice to the central lab at the end of the study. At each visit from visit

For peer review only

2 to 6, an additional EDTA blood sample will be collected for further 6-TG and 6-MMP testing and frozen at -80C.

Methotrexate

Washed erythrocyte for MTX levels will be obtained at visits 2, 4 and 6 and stored frozen at local centres. These samples will be sent on dry ice for central analysis to evaluate response to therapy and adverse effects in relation to drug levels.

Adalimumab

Adalimumab trough levels are measured after 3 injections of maintenance therapy (e.g. at visit 2) within the local laboratory (central lab testing available if local lab testing is unavailable). Dosing interval may be shortened to weekly in the event of low ADA levels (<8 mcg/ml) and negative ADA antibodies. Further samples should be obtained at visits 3, 4, 5 and 6 and should be frozen for later analysis within the central lab.

Pharmacogenetics

DNA for pharmacogenetics should be taken from patients randomised to MTX or AZA/6MP for multiplex genotyping of polymorphism related to drug metabolism to evaluate safety and response to therapy. Analysis will be performed at the end of the study, or earlier in those patients showing toxicity.

For peer review only

Ancillary Study

Patients unable to discontinue induction therapy or those randomised to thiopurine or MTX therapy who experience treatment failure may be invited to participate in the ancillary study (STEP-up ADA) until visit 5. Any initial maintenance therapy will be stopped and induction and maintenance regime for ADA as previously described will be commenced. Up to 3 additional study visits at 3-month intervals will be offered to these patients in order to obtain 12 months of follow up. A maximum of 68 patients can participate in this ancillary study allowing a 1:1 comparison of TOP-down ADA to STEP-up ADA therapy.

Unscheduled Visits

Unscheduled visits may be arranged based upon clinical requirements. As for scheduled visits per protocol treatment adaptions are possible if intolerance or failure of the study drug is detected. Subsequent scheduled visits will not be changed after an unscheduled visit.

Treatment Discontinuation

Patients who discontinue treatment before completing 12 months of study drug within either the main study or the ancillary study will receive a single follow-up visit. This will be either 12 months after the commencement of study treatment or at the point of inclusion in the ancillary study.

Modifications to the protocol while the study is being conducted will be relayed to all site staff by email and then onto their relevant ethical and regulatory boards. The current manuscript is based on protocol 5.1 last modified 28th May 2019.

For peer review only

Allocation Concealment and Blinding

For ethical reasons we decided against a double dummy design for blinding the patient, parents and care givers. Due to the differences in medication administration route and the significant nausea commonly associated with MTX blinding of the allocation to the patients, their families or their physicians is not possible. Where possible however, blinding of an alternative clinician to score the wPCDAI, PCDAI and PGA at each study visit should occur (prospective randomized open blind end-point (PROBE) evaluation).

Safety

The external and independent Advisory Board of PIBDSETQuality serves as an independent Data and Safety Monitoring Board which meets at pre-specified intervals with access to all data within the study. The principal investigator at each site is responsible for reporting any safety issues (adverse events, serious adverse events (SAEs), suspected unexpected serious adverse reactions), drop-outs, or any new information which may impact the study in any way. The principal investigator shall report to the sponsor all SAEs experienced by a study subject receiving an Adalimumab (Humira) within 24 hours of learning of the event regardless of the relationship of the event to the product. All SAEs are immediately sent to AbbVie pharmacovigilance by the sponsor. SAEs will be followed from the date of patient's signature of informed consent, until complete resolution or 30 days after the end of the study/patient's final study visit.

For peer review only

Box 5 – Criteria for premature termination of study treatment or participation

- Pregnancy at any stage
- Treatment failure as per protocol
- Failure to tolerate allocated treatment or alternatives as listed within the protocol
- Significant drug related side effects manifesting as significantly abnormal bloods results or adverse effects based upon the clinical judgement of the treating physician
- Request of participant to be withdrawn from treatment
- The judgement of the treating physician being that it is in the best interests of the participant to withdraw from study treatment
- Loss of participant to follow up
- Patient death

For peer review only

Participants may withdraw consent for further participation or data collection at any time without giving reason and without prejudicing further care or treatment. Patients will be permanently withdrawn from study treatment in the event of any of the situations outlined in Box 5. Patients should be provided with a study alert card for use in the event of an emergency.

Biochemical markers are monitored with a clearly defined protocol for adjustments to therapy based on abnormal results (e.g. neutropenia, pancreatitis, elevated liver enzymes).

Data Collection, Management and Monitoring

Patient CRFs are completed in a prospective manner using an electronic web-based system designed specifically by PIBDnet for this trial. In order to maintain data

security and integrity, the web-based data entry will be linked to a password secured Microsoft Access database, where data will be stored until time of analysis. Files will be saved on a code secured net-drive and backed-up following each data entry on a disk locked in a cabinet. Patients will be identified only by a study code assigned at the point of enrolment. Code of patient identifiers will be kept at each participating site. Handling of patient-identifiable is compliant with the legislation of each participating centre and the European General Data Protection Regulation (GDPR). Investigators will be invited to fax or email the paper source document to the coordinating site on a random basis to allow appropriate monitoring. Access to data with detailed information on study outcomes will be made available to other research groups on request and at the discretion of the principal investigators.

Monitoring arrangements are in place for all sites after initial site initiation. The monitoring visits will occur regularly partly dependant on recruitment rate at individual sites. The monitoring is perfomed usually by someone external to the clinical team.

Analysis and Statistical Methods

Descriptive statistics (mean, median, standard deviation, standard error, quartiles, minimum, maximum, and two-sided 95% confidence limits of mean and median) will be presented for each treatment of the low and high risk paediatric CD groups and where applicable, for the paired difference of each patient. Frequency tables will be presented where applicable.

Primary Analysis

For peer review only

Difference in the 12-month steroid/EEN free sustained remission rates between the treatment groups will be undertaken using Chi square test. Mantel Haenzel test will be used to combine data from all participating sites.

Secondary Analyses

Chi-square tests or Fisher's exact tests will be used to compare rates of remission, steroid intake, dropout and serious adverse events between the two arms of each risk group and between the low and high risk MTX groups. Logistic regression analyses may be performed to adjust for any imbalances in baseline covariates. To compare time to disease flare between the arms of each risk group and between high and low risk MTX groups, a Kaplan–Meier survival estimate will be used and the log-rank test of equality over strata. A Cox proportional hazard model will be constructed to obtain a hazard ratio after validation of the proportionality assumption and adjusting for possible confounding variables (including age and disease duration). Student's t tests or Wilcoxon rank sum tests will be used to compare growth, steroid dose, adverse events, changes in quality of life and patient reported outcomes between the two arms of each risk group and between the high and low risk MTX groups. The predictive value of faecal calprotectin levels, CRP, serum tests or other clinical predictors for response (including genomic and serological markers) will be assessed for each arm of the study using sensitivity, specificity, negative and positive predictive values or area under the ROC curve. Multivariate logistic regression analyses will then be performed.

For peer review only

Analyses will be performed using the R software (http://cran.r-project.org). All comparisons will be made using a 2-sided significance level of 0.05.

Sample Size Considerations

Estimated remission rates are based on recent analysis from the RISK study[18], indicating an advantage of early anti-TNF introduction over immunomodulator therapy. For the low risk group, it was hypothesized that 48% of children will be in remission at 12 months for the AZA/6MP arm versus 70% for the MTX arm. On the basis of this data with an alpha risk of 5% and a power of 80% a sample requirement of 88 patients per arm was calculated assuming a 10% loss of follow up. For the high-risk group, it was hypothesised that 40% of children will be in remission at 12 months for the MTX arm versus 65% for the adalimumab arm. To detect this difference with an alpha risk of 5% and a power of 80%, a sample size of 68 participants is necessary, again assuming a 10% loss of follow-up. In total 312 participants will be included in the study (176 low-risk group; 136 high risk group).

Patient and Public Involvement

Patients were not involved in the development of this study; however, the French patient charity AFA Crohn, RCH, France was involved in study design and critically reviewed and commented upon all aspects of the trial.

For peer review only

Discussion

REDUCE-RISK in CD is the first multicentre international RCT aiming to compare three different medication strategies for maintenance of remission in newly diagnosed CD based upon a risk stratification protocol. During the 12-month follow up period the effects of the differing management strategies will be assessed via data collected and outcome measures as defined above in order to analyse the efficacy and safety of each medication and better define the most appropriate first-line maintenance immunomodulators to be used in specific subsets of CD patients. As a group we speculatively hypothesise that MTX will be superior to thiopurines for maintaining remission in CD in the low risk group although in the absence of head to head studies prior to this one this study will provide randomised data to address this. Additionally, from our own work and others we know response to induction therapy is an important prognostic marker and we wanted to allow the induction treatment to have a chance to work before we assigned high or low risk status.[24,25] Thus it was a pragmatic compromise with the timing of introduction of the maintenance treatment to give the induction treatment long enough to show its effect while recognising both treatments have a “lag period” of a few weeks before they become fully effective.

We also hypothesise that ADA will be superior to MTX in the high-risk group based upon the results from the RISK study.[18] Of note Adalimumab (Humira) was chosen to allow delivery of the study out of hospital, to reduce drug costs and it allowed single therapies to be compared with each other. Practically if we had used

For peer review only

Infliximab (Remicade) then we would have needed to use combination therapy which we did do not want to do as it would have further complicated the trial design.

In addition to this, the ancillary study will compare outcomes in ADA treated patients from inclusion (Top-down) versus patients switched to ADA due to failure of immunomodulators (Step-up), with the potential to stratify which patients might benefit from such a top-down treatment strategy. We acknowledge that comparison of the ancillary group with the group randomised from baseline to ADA is not randomised and may be subject to selection bias noting the ancillary group have failed or been intolerant to initial therapy. However we feel it is important to include this to allow us to compare the trial with studies which have allocated patients directly to anti-tnf (RISK, TISKids) and to see how many patients benefit from "rescue therapy" after failure of their initial allocation.

The design and completion of interventional studies in PIBD is a recognised challenge between rigorous study design methodology and pragmatic considerations around feasibility and completion within a paediatric dataset.[23] This particular study is limited by the inability to blind the treatment allocation to the patients, their families or their treating physician due to the differences in medication administration route and the side effects commonly associated with the study medication. Although the protocol advises that where possible blinding of an alternative clinician to score disease assessment at each study visit should occur in order to obtain prospective randomized open blind end-point (PROBE) evaluation this may be practically difficult in smaller centres where staff are familiar with the majority of their patient cohort.

For peer review only

ETHICS AND DISSEMINATION

The study is being conducted according to the principles of the Declarations of Helsinki and to date has been approved by all participating sites as listed within supplementary Table 1. Clinical trials authorisation and ethics approval has been obtained from the local ethics review committees of these participating nations and centres. The Standard Protocol Items: Recommendations for Interventional Trials (SPIRIT) guidelines[26] were adhered to in the production of the protocol for this trial (see uploaded material for details).

Consent

Patients and their caregivers are provided with study-specific information including an explicit description of the study outline and alternatives for participation. It is made clear to all patients approached that declining to participate in the study will not jeopardize the quality of subsequent care received. After a period of consideration, if agreeable, the patient’s parent or caregiver is asked to sign consent forms with age-appropriate assent obtained from the child where relevant (see appendix 1 for model consent forms). The signed forms are filed within the patient’s medical record with a copy provided to the participant and their caregiver. Consent will be obtained by site staff with the relevant training and who are identified as assigned on the delegation log. Participants taking part in the ancillary study will not be re-consented.

Dissemination

For peer review only

Results of the study will be submitted for publication within a peer-reviewed journal. In accordance with the H2020 general grant agreement, the dissemination process will ensure open access to the scientific publications resulting from this project. Journal authorship guidelines will be adhered to and there are no plans to use professional writers.

AUTHOR CONTRIBUTIONS

RH prepared the draft manuscript with comments and review from all authors. RKR, MA, LR, NC, SK, AL, DT, GV, MN and LB and FMR were involved in the conception, design, planning and then drafting of the original research protocol and RKR, RH, MA, LR, NC, SK, AL, DT, GV, MN and LB and FMR provided critical review of the manuscript and approved the final uploaded draft**.**

As sponsor PIBDnet has full responsibility and control for the original study design, collection, management, analysis, and interpretation of data, including writing of the report and the decision where to submit the report for publication.

FUNDING

This work was supported as part of the PIBD-SETQuality (Paediatric Inflammatory Bowel Diseases Network for Safety, Efficacy, Treatment and Quality improvement of care) project funded by the European Commission Horizon 2020 - Research and Innovation Framework Programme (grant number 668023). ADA (Humira ®) is provided by AbbVie.

The main study sponsor is PIBDNet. PIBDNet is the EU legal representative for the study. The specific contact for the sponsor is Frank Ruemmele (Service de Gastro-entéroloegie, Hôpital Necker Enfants Maldes, 149 rue de Sèvres, 75015 Paris, France).

For peer review only

COMPETING INTERESTS

RKR is supported by an NHS Research Scotland Senior Research Fellowship, and has received speaker's fees, travel support, and/or participated in medical board meetings with Nestle, MSD Immunology, AbbVie, Dr Falk, Takeda, Napp, Mead Johnson, Nutricia & 4D Pharma. FMR has received speaker fees from Shering-Plough, Nestlé, MeadJohnson, Ferring, MSD, Johnson & Johnson, Centocor, AbbVie; has served as a board member for SAC:DEVELOP (Johnson & Johnson), CAPE (AbbVie), LEA (AbbVie); and has been invited to MSD France, Nestlé Nutrition Institute, Nestlé Health Science, Danone, MeadJohnson, Takeda, Celgene, Biogen, Shire, Pfizer, and Therakos. DT received consultation fee, research grant, royalties, or honorarium from Janssen, Pfizer, Hospital for Sick Children, Ferring, Abbvie, Takeda, Biogen, Atlantic Health, Shire, Celgene, Lilly, Neopharm, Roche. LdR received consultation fee, research grant, or honorarium from ZonMw, ECCO, Shire, Malinckrodt, Nestlé, Celltrion, Abbvie and Pfizer. MA received consultation fee and honorarium from Abbvie. SK received consultation fee, research grant, or honorarium from Danone, Nestec-Nutrition, Abbvie, Takeda, Celgene, Shire, Pfizer, Biogaia, Janssen, Berlin-Chemie; Mead Johnson, Vifor, Pharmacosmos, ThermoFisher

Remaining authors: nil competing interests declared.

FIGURE LEGENDS

Figure 1 – Study Design of the REDUCE-RISK in CD trial

M2 = Month 2, V2 = visit 2.

For peer review only

REFERENCES

1. Ruemmele FM, Veres G, Kolho KL et al. Consensus guidelines of ECCO/ESPGHAN on the medical management of pediatric Crohn's disease. *J Crohns Colitis*. 2014;8(10):1179-207.

2. Escher J. Budesonide versus prednisolone for the treatment of active Crohn's disease in children. *Eur J Gastroenterol Hepatol*. 2004;16(1):47-54.

3. Heuschkel R, Menache C, Megerian J et al. Enteral Nutrition and Corticosteroids in the Treatment of Acute Crohn's Disease in Children. *J Pediatr Gastroenterol Nutr.* 2000;31(1):8-15.

4. Chande N, Patton P, Tsoulis D et al. Azathioprine or 6-mercaptopurine for maintenance of remission in Crohn's disease. *Cochrane Database Syst Rev*. 2015;(10):CD000067

5. Markowitz J, Grancher K, Kohn N et al. A multicenter trial of 6-mercaptopurine and prednisone in children with newly diagnosed Crohn's disease. *Gastroenterology*. 2000;119(4):895-902.

6. D'Haens G, Geboes K, Rutgeerts P. Endoscopic and histologic healing of Crohn's (ileo-) colitis with azathioprine. *Gastrointest Endosc*. 1999;50(5):667-71.

For peer review only

7. Feagan BG, Fedorak RN, Irvine EJ et al. A comparison of methotrexate with placebo for the maintenance of remission in Crohn's disease. North American Crohn's Study Group Investigators. *N Engl J Med*. 2000;342(22):1627-32.

8. Feagan BG, Rochon J, Fedorak RN et al. Methotrexate for the treatment of Crohn's disease. The North American Crohn's Study Group Investigators. *N Engl J Med.* 1995;332(5):292-7.

9. Willot S, Noble A, Deslandres C. Methotrexate in the treatment of inflammatory bowel disease: An 8-year retrospective study in a Canadian pediatric IBD center. *Inflamm Bowel Dis*. 2011;17(12):2521-2526.

10. Patel V, Wang Y, MacDonald J et al. Methotrexate for maintenance of remission in Crohn's disease. *Cochrane Database Syst Rev*. 2014;(8):CD006884

11. Hyams J, Crandall W, Kugathasan S et al. Induction and maintenance infliximab therapy for the treatment of moderate-to-severe Crohn's disease in children. *Gastroenterology*. 2007;132(3):863-73.

12. Ruemmele F, Lachaux A, Cézard J et al. Efficacy of Infliximab in Pediatric Crohn’s Disease: A Randomized Multicenter Open-Label Trial Comparing Scheduled to On Demand Maintenance Therapy. *Inflamm Bowel Dis*. 2009;15(3):388-394.

For peer review only

13. Hyams JS, Griffiths A, Markowitz J et al. Safety and efficacy of adalimumab for moderate to severe Crohn's disease in children. *Gastroenterology*. 2012;143(2):365-74.e2.

14. Russell R, Wilson M, Loganathan S et al. A British Society of Paediatric Gastroenterology, Hepatology and Nutrition survey of the effectiveness and safety of adalimumab in children with inflammatory bowel disease. *Aliment Pharmacol Ther*. 2011;33(8):946-953.

15. Ardizzone S, Bollani S, Manzionna G et al. Comparison between methotrexate and azathioprine in the treatment of chronic active Crohn's disease: a randomised, investigator-blind study. *Dig Liver Dis*. 2003;35(9):619-27.

16. Oren R, Moshkowitz M, Odes S et al. Methotrexate in chronic active Crohn's disease: a double-blind, randomized, Israeli multicenter trial. *Am J Gastroenterol*. 1997;92(12):2203-9.

17. Maté-Jiménez J, Hermida C, Cantero-Perona J et al. 6-Mercaptopurine or methotrexate added to prednisone induces and maintains remission in steroid-dependent inflammatory bowel disease. *Eur J Gastroenterol Hepatol.* 2000;12(11):1227-1233.

For peer review only

18. Walters TD, Kim MO, Denson LA et al. Increased effectiveness of early therapy with anti-tumor necrosis factor-alpha vs an immunomodulator in children with Crohn's disease. *Gastroenterology*. 2014;146(2):383-91.

19. Torres J, Caprioli F, Katsanos KH et al. Predicting outcomes to optimize disease management in inflammatory bowel diseases. *J Crohns Colitis*. 2016;10(12):1385-1394

20. Cozijnsen M, van Pieterson M, Samsom J et al. Top-down Infliximab Study in Kids with Crohn's disease (TISKids): an international multicentre randomised controlled trial. *BMJ Open Gastroenterol*. 2016;3(1):e000123.

21. Levine A, Koletzko S, Turner D et al. The ESPGHAN Revised Porto Criteria for the Diagnosis of Inflammatory Bowel Disease in Children and Adolescents. *J Pediatr Gastroenterol Nutr*. 2013;58(6):795-806.

22. Levine A, Griffiths A, Markowitz J et al. Pediatric modification of the Montreal classification for inflammatory bowel disease. *Inflamm Bowel Dis.* 2011;17(6):1314-1321.

23. Turner D, Griffiths AM, Wilson D et al. Designing clinical trials in paediatric inflammatory bowel diseases: a PIBDnet commentary. *Gut*. Published online first: 12 April 2019 Apr. doi:10.1136/gutjnl-2018-317987

For peer review only

24. Ziv-Baran T, Hussey S, Sladek M, et al. Response to treatment is more important than disease severity at diagnosis for prediction of early relapse in new-onset paediatric Crohn's disease. *Aliment Pharmacol Ther* 2018; **48**(11-12): 1242-50.

25. Levine A, Chanchlani N, Hussey S, et al. Complicated Disease and Response to Initial Therapy Predicts Early Surgery in Paediatric Crohn's Disease: Results From the Porto Group GROWTH Study. *Journal of Crohn's & colitis* 2020; **14**(1): 71-78.

26. Chan A, Tetzlaff J, Altman D et al. SPIRIT 2013 Statement: Defining Standard Protocol Items for Clinical Trials. *Ann Intern Med*. 2013;158(3):200-207.

For peer review only

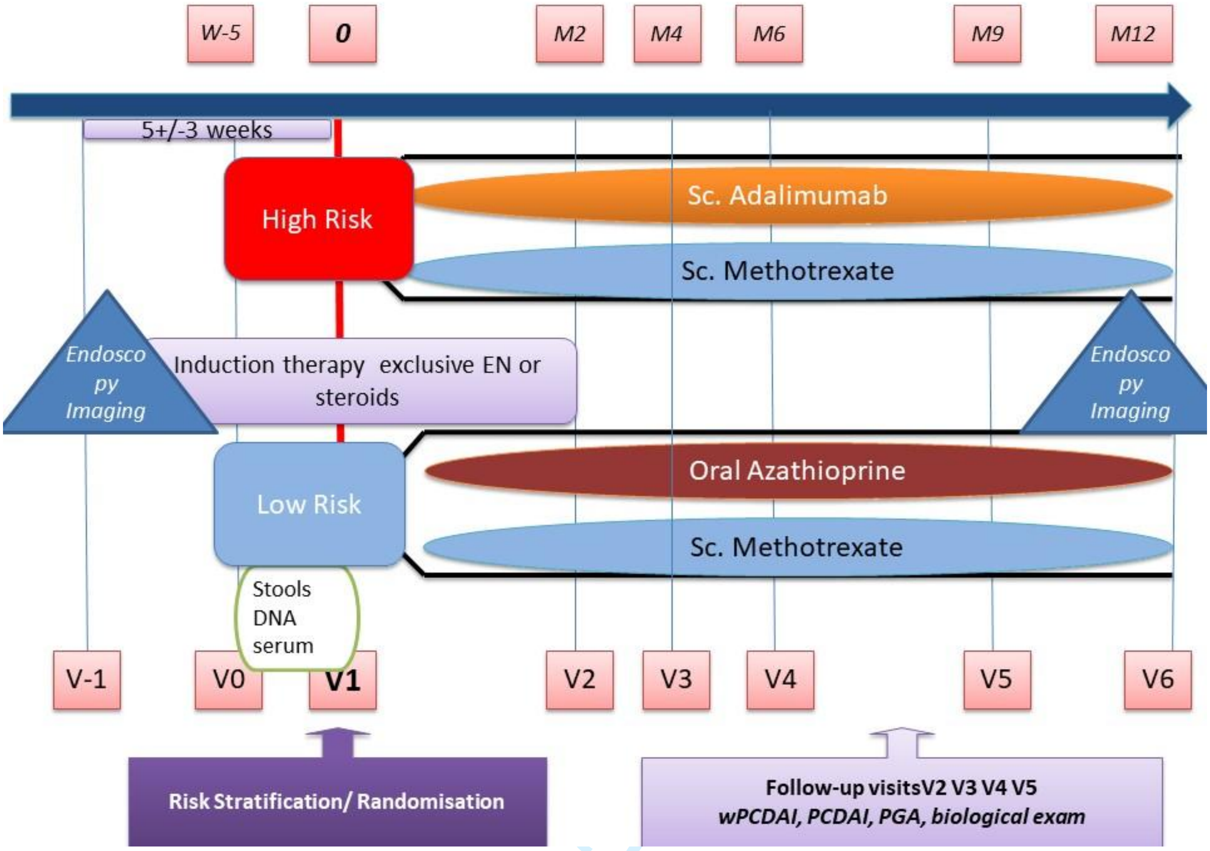

Figure 1 – Study Design of the REDUCE-RISK in CD trial

Participating Sites – REDUCE-RISK in CD Study			
**Country**	**City**	**Site**	**Ethics committee**
**Belgium**	Brussels	Universitair Ziekenhuis	Commissie Ethiek, UZ **Brussel**
	Brussels	Clinique Saint Luc UCL	
	Liège	Clinique de l'Espérance	
	Brussels	HUDERF	
**Canada**	Toronto	SickKids	SickKids Research Ethics Board, **Toronto**
**Czech Republic**	Prague	FN Motol	Eticka Komise, **Plzen**<br>Eticka Komise, VFN **Praha**<br>Eticka Komise, FN Motol **Praha**
	Plzeň	FN Plzeň	
	Prague	First Medical Faculty	
**France**	Paris	Hôpital Necker Enfants Malades	CPP Hôpital Necker, **Paris**
	Paris	Hôpital Robert Debré	
	Paris	Hôpital Armand Trousseau	
	Le Havre	Hôpital Jacques Monod	
	Nancy	Hôpitaux de Brabois	
	Toulouse	Hôpital des Enfants	
	Tours	Hôpital Clocheville	
	Caen	CHU Caen Côte de Nacre	
	Marseille	Hôpital de la Timone	
**Germany**	Munich	Childrens Hospital	Ethikkommission LMU, **München**
	Ulm	Universitätsklinikum	
	Hannover	MHH Kinderklinik	
	Giessen	UKGM	
	Berlin	Charite Hospital	
**Greece**	Athens	Children's Hospital "AGIA SOFIA"	Ethics Committee, **Athens**
**Israel**	Jerusalem	Shaare Zedek Medical Center	Helsinki Committee, Schneider Medical Center, **Petah Tikva**<br>Ethics and Research Committe, Wolfson Medical Center, **Tel Aviv**<br>Institutional Review Board, SZMC, **Jerusalem**
	Tel Aviv	Wolfson Medical Center	
	Petah Tikva	Schneider Children's Medical Center	
	Ramat Gan	Sheba Medical Center	
	Haifa	Rambam Medical Center	
**Italy**	Rome	Università degli Studi di Roma La Sapienza	Comitato Etico dell'Universita' "SAPIENZA", **Roma**<br>Comitato Etico Regionale per la Sperimentazione Clinica della Regione Toscana Sezione, **Firenze**<br>Comitato Etico, Servizio Saintario Regionale, **Bologna**
	Bologna	Maggiore Hospital	
	Florence	Azienda Ospedaliero Universitaria	
	Parma	Azienda Ospedaliero	
	Rome	Opsedale Pediatrico Bambino Gesù	
**Netherlands**	Rotterdam	Erasmus Medical Center	Medische Etische Toetsings Commissie, Erasmus MC,

For peer review only

			**Rotterdam**
**Poland**	Warsaw	Centrum Zdrowia MDM	Bioethical Commission at the Institute of Polish Mother's Health Center, **Lodz**
	Białystok	Uniwersytecki Dziecięcy Szpital Kliniczny	
	Łódź	Instytut Centrum Zdrowia Matki Polki	
**United Kingdom**	Glasgow	The Royal Hospital for Children	North West - Liverpool East Research Ethics Committee, NHS, **Manchester**
	London	Royal London Children's Hospital, Barts Health NHS Trust	
	Edinburgh	Sick Children's Hospital	
	Birmingham	Children's Hospital	
	Oxford	John Radcliffe Hospital	

Supplementary Table 1 – Sites participating in REDUCE-RISK in CD

For peer review only

PIBD-SETQuality

INFORMED CONSENT FORM

Parents/Guardian Informed Consent Form for Participation of a Minor in a Clinical Trial

Risk-stratified randomized controlled trial in paediatric Crohn's Disease: Methotrexate versus azathioprine or adalimumab for maintaining remission in patients at low or at high risk for aggressive disease course, respectively – a treatment strategy.

Dear parents,

Your child's doctor, Dr…………………………, working at ………………………………….. Hospital, propose your child to participate in a clinical trial related to its disease.

It is important to read this note carefully before taking any decision. Do not hesitate to ask the physician all the questions you may have about it.

The participation of your child is based on volunteering. Therefore, your child can refuse to participate or stop its participation in the trial at any time, all of this without prejudice to the patient's right to receive the standard treatment.

If you refuse your child to participate, he/she will still receive the best medical support.

Purpose of the research and trial's objectives

Your child has just been diagnosed with Crohn's disease. The disease is caracterised by chronic inflammation of the digestive track (bowel/colon). This disease changes over between remission period and relapse period. There are efficient drugs able to prevent relapse and to maintain remission. In order to reduce the likelihood of long-term complications, induction treatment has already been prescribed to your child. This first treatment has to be followed up by a maintenance treatment that will be introduced to avoid the inflammation from returning. Consensus guidelines of ECCO / ESPGHAN (french and european IBD specialized organizations) recommend 3 efficient treatments: either immunosuppressive treatment with Thiopurines (azathioprine and 6-mercaptopurine), or Methotrexate or anti TNF (adalimumab).

So far, no clinical trial has been conducted to compare those 3 treatments in children with Crohn's disease and to answer the following question: "Which treatment is the most efficient, for which patient and/or in which situation?"

Progression of Crohn's disease is not the same for all patients. That's why this study will first classified all children in high and low risk groups based on more or less severe course of Crohn's disease. The lower risk group will be randomized (which is like tossing a coin) to receive either thiopurines or methotrexate as maintenance treatment. The high risk group will be randomized to receive either methotrexate or adalimumab. Results will show whether there is different efficiency between the 3 drugs for patients with a more or less severe disease.

Sponsor

PIBD-Net (www.pibd-net.org) is a global, international and non-profit organisation gathering physicians and researchers specialised in inflammatory bowel diseases. The acronym stands for Pediatric Inflammatory Bowel Diseases Network and it is present in 31 countries (Europe, North America, Australia and Japan). This organisation is dedicated in improving the medical care of children with inflammatory bowel disease through the establishment of clinical researches.

PIBD-Net and partners received funding from European Commission for Horizon 2020 program (project no.668023) in order to perform this research.

The approximate number of participants and duration of follow-up

A total of 312 new-onset children with Crohn's disease (136 in that high-risk group and 176 in the low risk) will be enrolled in many sites around the world. The period of recruitment is 45 months and your child will be followed up for 12 months after enrolment.

For peer review only - http://bmjopen.bmj.com/site/about/guidelines.xhtml

PIBD-SETQuality

European Commission

What will happen to your child during the trial?

If you agree to have your child participating in this study, your child will be first directed into one of two groups based on certain predictors of its disease (such as its location and severity). You will know which group your child is in. Next, your child will be randomized to receive maintenance treatment namely:

- **METHOTREXATE or AZATHIOPRINE for the low risk group** ;
- **METHOTREXATE orADALIMUMABfor the high risk group** ;

You cannot choose the treatment group but you will know which drug your child will receive.

You will not be asked to come to clinic just because of this study which is designed to mirror regular follow-up in clinic. After signing the informed consent allowing your child to be part of this research, your child will have clinic visit every 2 months during the first 6 months and then every 3 months during the last 6 months. At each visit, a clinic examination is performed and blood, urine and stool samples are collected (this process follows our standard clinical practices of our patients not involved in this protocole).

Your doctor, your child and yourself will be asked to complete short questionnaires to evaluate the quality of life of your child. Most of the recorded data for this study is needed anyway as part of a regular visit but there might be a few more questions we will ask you for this study.

We will also contact you over the phone at week 4 to ask how your child feels regarding its Crohn's disease, whether your child has any bad reactions to the medications your child will receive and check your child compliance to the treatment.

To optimize the medications we prescribe, we will draw 12 ml (3-4 teaspoons) of blood at inclusion visit, 10ml at visit V2, and then 5ml at each next study visits to measure the level of the medications your child will receive. A urine sample will be collected at inclusion visit and a DNA sample (either 5ml of blood or buccal swab) will be collected at the beginning of the study, and also in case of drug intolerance.

We will also collect your child stool (poop) six times during the year to measure the amount of inflammation in its bowel as well as bacteria flora in its intestine.

We will evaluate whether the drugs work based on

- completed questionnaires
- clinic examination
- results of biological samples

Optional Ancillary study (« ADA STEP-up »)

In case of failing (intolerance or relapse) of your child immunomodulator therapy (: either azathioprine/6MP or methotrexate), your child will be invitated to participate in the ancillary study. If you agree, your child will be prescribed adalimumab during 12 months.

This adalimumab treatment can increase the study duration by a maximum of 9 months, meaning a maximum of 3 additional visits. Those visits are identical to the regular follow up study visits.

The expected benefits to the participant or to others because of the trial

The medications your child will receive in this study are not experimental and thus there are no direct benefit for using these drugs that are available outside of the study. However, your child will be monitored closely to ensure optimization of the treatment by adapting drug amounts based on new analyses (urine, DNA, blood and stool samples). In addition, patients involved in this study have access to molecular analyses in order to better understand why a patient is less responsive than expected. That might result in a more tight control of the disease and better monitoring of the treatment of your child.

For peer review only

For peer review only - http://bmjopen.bmj.com/site/about/guidelines.xhtml

PIBD-SETQuality

European Commission

After study completion, we will have the required data to recommend how to use these medications in new children who develop Crohn's disease.

Risks added by the research

As previously mentionned, all medications used in this trial are not experimental and are being used very often in clinical practice in children/adolescents and adults with Crohn's disease. There is no additional risk compares to regular clinical practices. The known risks and discomfort that may be anticipated are listed below.

Known risks and discomfort that may be anticipated

The medications yourchild will receive in this study are not experimental but used in regular practices. They can also be associated with side effects (all described in the corresponding drug information sheet).

- ✓ **METHOTREXATE: weekly subcutaneous (under the skin)** This drug may be associated with side effects mainly in the day of the injection including flu-like symptoms, nausea, vomiting, headache or fatigue. Your child will be asked to take a vitamin called folic acid which will reduce these non-dangerous side effects. This injection may cause slight discomfort.METHOTREXATE may cause reduced blood counts, especially white blood cells and elevated liver enzymes. Thoses parameters will be checked regularly. In this study, molecular analyses will be performed to screen patients who can't tolerate METHOTREXATE..This drug causes an unusual sensitiveness to the sun (however, there is no data stating that it increases the risk of cancer or lymphoma). METHOTREXATE can cause foetal abdormalities so pregnancy is not allowed and efficient contraceptive is essential (for both male and female).
- ✓ **AZATHIOPRINE (or 6MP): to be taken orally.** THIOPURINES may cause reduced blood counts, especially white blood cells and elevated liver enzymes. Thoses parameters will be checked regurlarlyIn this study, molecular analyses will be done in order to screen patients who can't tolerate those drugs. In some rare cases (<3%), the drug may cause inflammation in the pancreas which is usually mild and not dangerous. As all drugs, THIOPURINES can't be tolerated by some patients due to allergy. Approximately 10% of children will not tolerate the drug because of nausea, vomiting, tummy pain, diarrhea, headaches or fever..THIOPURINES are associated with an increased infectious risk (about 1%). Infections are likely cause by viruses. In rare cases, THIOPURINES may increase risk for blood cancer called lymphoma (especially for patients > 65 year old).–This drug causes an unusual sensitiveness to the sun and can be associated with skin cancer in case of significant sun exposure.

ADALIMUMAB: subcutaneous (under the skin) injections every 2 weeks Adalimumab is associated with minor pain during the injection and local reactions could appear with minimal significance..ADALIMUMAB is associated with an increased infectious risk. However, serious infections are uncommon. Before starting ADALIMUMAB treatment, tuberculosis must be excluded. With time, the effect of adalimumab may wain as a result of the development of antibodies against the drug. Skin inflammatory damages (such as « psoriasis ») were observed in some patients. Anti TNF drugs have been closely monitored since their use as standard treatment. Those drugs may be responsible of heart failure for patients with severe heart disease, hepatitis, decreasing blood cells, demyelinating neurologic disease, or lupus (without affecting main organs). In addition, some cases of cancer have been notified in patient treated by ADALIMUMAB, but risks of cancer is slightly increased only for melanoma. Number of cancer seems not to be increased compared to patients with Crohn's diseases and without being treated with those drugs.

Circumstances under which participation in the medical trial may be discontinued in accordance with the decision of the investigator or the Sponsor:

a. The doctor has the right to take your child out of the study at any time. This will be made after clinical considerations, your child's side effects from the drugs, intolerance to the drugs or lose of response.

b. Regulatory authorities (Ministery of Health or Ethics committee), may stop your child participating in the study.

For peer review only - http://bmjopen.bmj.com/site/about/guidelines.xhtml

PIBD-SETQuality

European Commission

An explanation of alternative treatments, their advantages and disadvantages, if any, for the participant:

The current standard therapy for maintenance therapy in Crohn's disease is either METHOTREXATE or thiopurines or anti-TNF biologics for the more severe Crohn diseaes. This is exactly the medications given also as part of this study. The difference is that instead letting you and the doctor choose between the options, the choice is standardized based on predictive variables of your disease and randomization. If you choose not to have your child participating in the study, your child will likely receive anyway one or more of these three drugs. The only exception would be that if anti-TNF is prescribed, either adalimumab or infliximab can be given and as part of this study your child will receive adalimumab only. However, your child will not have access to molecular analyses described in this protocole with a close monitoring of drug safety. Indeed, those 'new" analyses are not done in the standardclinical practise of Crohn's disease.

If you participate in this study, what will you have to do more than usual ?

If you agree to have your child participating in this study, please make sure to follow the listed points belowPlease come at your appointements with your child. If not possible, please inform its physician as soon as possible

- Please ensure that your child takes the treatment as instructed by its doctor
- Please inform the physician involved in the study of any event happening during the research (such as hospitalization,…)
- Your child must not participate in any other clinical trial that involves the use of an investigational product throughout the course of this trial. It is to avoid accidents such as possible interactions between medicines.

Biological samples collected during this research project

If you agree to have your child participating in this research, additional blood, urine and stool samples will be collected at the same time as our standard clinic samplings. Please see below:

- ✓ 10ml of blood during inclusion visit (on randomisation day).
- ✓ 5ml of blood (PAX tube) during inclusion visit for RNA analyses
- ✓ 10ml of blood at follow up visits M2 (2 months after inclusion)
- ✓ 5 ml of blood at follow up visits M4, M6, M9 and M12 (4, 6, 9, 12 months after inclusion)
- ✓ Stool sample at inclusion visit and follow up visits M2, M4, M6, M9 and M12 (2, 4, 6, 9, 12 months after inclusion visit).
- ✓ A DNA sample will be collected at inclusion visit and in case of intolerance of one of the drugs for DNA analyses.
- ✓
- ✓ Urine sample (15ml) will be collected at M2 visit (2 months after inclusion).

Those samples will be sent to specialized laboratories in order to be used to perform specific studies such as adalimumab, methotrexate, thiopurine analyses and serology, genetic (both DNA and RA), microbiology studies. They will also be re-used for further testing on Crohn's disease, its diagnosis and its treatment as well as efficacy and tolerance by molecular ("omic") analyses.

At any time, you can request to your clinician to have those biological samples destroyed or not to be used for further researches.

Confidentiality

As part of biomedical research in which PIBD-Net sponsor proposes your child's participation, treatment of personal data will be set up to analyse results of this research based on its aim. Therefore, your child medical data and quality of life will be transferred to PIBD-Net sponsor. Those data will be anonymous and identified by a coded number and its initials. Those confidential data could be transferred to local and foreign authorities. If your child has to be withdrawn for any reasons, collected data prior its withdrawal will be used unless you do not want

For peer review only

For peer review only - http://bmjopen.bmj.com/site/about/guidelines.xhtml

PIBD-SETQuality

European Commission

them to. Then, you will have to inform the physician accordingly.

According to the EU General Data Protection Regulation (GDPR) dated on 26May2018, you have the right to access to your child and your personal data, modify them and oppose the use of your child and yourdata. You have also the right to request that your child and your personal data are erased, are limited in use, and to ask for a complete copy of all data collected from you and your child for the study. You can contact the Data Privacy Officer (DPO) of the sponsor at any time at dpo@pibd-net.org for any request regarding your child and your personal data.

Data collected for the study are transferred outside of the EU, as our database is based in Israel. However, we guarantee that data protection will be as strict as requested by GDPR.

Voluntary participation

Your participation in this research is entirely voluntary. It is your choice whether to have your child participating or not, all the services your child receives at this hospital will continue and nothing will change. If you choose not to participate in this research project, your child will be offered the treatment that is routinely offered in this hospital for Crohn’s disease. You may change your mind later and stop participating even if you agreed earlier.

Right to refuse or withdraw

Your child does not have to take part in this research if you do not wish to do so and refusing to participate will not affect its treatment in any way. Your child will still have all the benefits that it would otherwise have at this hospital. You may stop participating in the research at any time that you wish without losing any of its rights as a patient here. Its treatment at this hospital will not be affected in any way.

Alternatives to participating

If you do not wish that your child takes part in the research, your child will be provided with the established standard treatment available at this hospital.

Reimbursement

There is no reimbursement for participating in this study. There are no special visits to the hospital excepted during this study. All DNA, blood, urine and stool samples will be taken at the time of a routine clinic visit.

This proposal has been reviewed and approved by [name of the local IRB], which is a committee whose task it is to make sure that research participants are protected from harm.

For peer review only

For peer review only - http://bmjopen.bmj.com/site/about/guidelines.xhtml

PIBD-SETQuality

European Commission

Informed consent form

We, the undersigned :

M, Miss, *(name, first name of parent/legal guardian)* …………………………………………………………..

M, Miss, *(name, first name of parent/legal guardian)* …………………………………………………………..

I, M, Miss, ……………………………………………………………………..*(name, first name of parent)* hereby declare that my consent below has been given voluntarily and that I have understood all of the above. I undertake to also inform the child's father/mother *(please cross off as appropriate)* of my consent for the participation of our child in the clinical trial. If the child's father/mother *(please cross off as appropriate)* does not agree to affix his/her consent to mine, I undertake to inform the physician in-charge and to withdraw my consent for the participation of my child in the clinical trial. I have also received a lawfully and dated copy of this informed consent form

I agree that my child (name, first name of the child)…………………………………………………….**takes part of the study named "***Risk-stratified randomized controlled trial in paediatric Crohn's Disease : Methotrexate versus azathioprine or adalimumab for maintaining remission in patients at low or at high risk for aggressive disease course, respectively – a treatment strategy* " , managed by PIBD Net. It has been explained to us by *(name, first name of explaining investigator /sub investigator, phone,)*…………………………………………. ……… ……………………………………………………………………………………physician in this clinical trial.

- We hereby declare that we agree for our child to participate in the clinical trial as detailed in this document
- Our child has been informed and agreed to take part of this clinical trial.
- We had the opportunity to ask all the questions we had to the physician who explained potential risks and constraints linked to our child participation in this clinical trial.
- We received appropriate answers to all our questions
- We hereby declare that at the time of signing this document, our child is not participating in another clinical trial that involves the use of any investigational product, and that we undertake that our child will not participate in any other clinical that involves the use of an investigational product throughout the course of this trial.
- We declare that our child has a health insurance.
- .We hereby declare that we are free to choose that our child will not participate in the clinical trial, and that we are free to stop our child participation in the trial at any time, and all of this without prejudice to our child's right to receive the standard treatment. Then, we will inform the physician whether data collected prior our decision can be used or not.
- We have been informed that the doctor has the right to take our child out of the study at any time, if needed.
- That in case of completing a questionnaire – we are entitled not to answer all or some of the questions in the questionnaire.
- We are informed that samples collected during this clinical trial will be kept and used for further testing on Crohn's disease. We can decide at any time not to have those samples used by informing our child physician.
- That we are guaranteed confidentiality concerning the identity of the patient and that of the parents/guardians. This confidentiality will be kept by all those concerned with and involved in the clinical trial, and their identity will not be disclosed in any publication.
- That the Medical Institution has arranged for appropriate insurance coverage of the investigators, physicians and medical staff involved in the clinical trial, against claims filed by clinical trial participants and/or third party claims related to the clinical trial, either during the course of the trial or thereafter. This is

For peer review only

For peer review only - http://bmjopen.bmj.com/site/about/guidelines.xhtml

PIBD-SETQuality

European Commission

without prejudice to our rights under the law.

- That in case of pregnancy during the course of the clinical trial, the girl/woman will be counselled (by the principal investigator) concerning the possible effects on the foetus and the fate of the pregnancy, including the possibility of discontinuing the pregnancy.
- We hereby declare that our below consent has been given voluntarily and that we have understood all of the above mentioned. We also received a lawfully signed and dated copy of this informed consent.
- By signing this consent form, we authorize the sponsor of the clinical trial, the Institutional Helsinki Committee, the auditing entity at the Medical Institute and the Ministry of Health direct access to the patient's medical file, to verify the clinical trial methods and the clinical data. This access to our child medical information will be performed with confidentiality maintained, according to the laws and procedures of maintaining confidentiality.
- We declare that we are informed and give our approval to receive all information related to our child participation in this clinical trial. We know that data will only be used for treatment and follow up cares
- We hereby declare that we know and agree to have the information on our child's participation in the clinical trial provided to his/her attending physician at the HMO/Health care Services with which our child is insured, in case the clinical trial involved the provision of services : performing medical examinations or supplying devices or products or implants. We know that the HMO will not use this information for purposes other than medical treatment and follow up

I agree to have my child participating in the ancillary study (« ADA STEP-up »)

Yes ☐ No ☐ *[please tick]*

**Signature of parents or guardians/representatives of the patient**	**Signature of the child**
Name, First Name :________________________<br>Date : Signature :<br><br>Name, First Name :________________________<br>Date : Signature :	Name, First Name :________________________<br>Date : Signature :

Declaration of the Investigator/Sub-Investigator :This consent was obtained by me after I have explained all the above mentioned to the parents (or guardians) of the clinical trial participant and ensure that all my explanations were understood by them.

**Investigator/Sub-investigator' Signature :**
Name, First Name: __________________________________
Date: Signature :

For peer review only

For peer review only - http://bmjopen.bmj.com/site/about/guidelines.xhtml

PIBD-SETQuality

European Commission

This is a triplicate document. First / original copy to be kept by the investigator for 15 years, second copy to be given to parents or legal guardians, third copy to be kept in Investigator files (under sealed envelope).

For peer review only

For peer review only - http://bmjopen.bmj.com/site/about/guidelines.xhtml

PIBD-SETQuality

European Commission

Informed consent for Genetic Analyses

Hereby declare that we agree for genetic examinations of our child to study genes involved in tolerance / non tolerance of the drugs by molecular ("omic") analyses and analyses of drug efficacy in Crohn disease's patients.

Hereby declare that we agree that all recorded data collected during this trial including genetic data can be processed by the sponsor or acting as sponsor. I understand that, as stipulated in the General Data Protection Regulation, I can access, modify, erase or ask for a copy of my child's personal data and my personal data at any time, by asking to the investigator who will contact the sponsor.

We can decide not to participate anymore in the genetic part of the trial by informing our doctor who will inform the sponsor.

Yes ☐ No ☐ *[please tick]*

Hereby declare that we agree that all biological samples collected during this trial can be used for future genetic research on Crohn's disease.

Yes ☐ No ☐ *[please tick]*

Parents/guardians Signature:

Name, First name:

Date : Signature :

Name, First name:

Date : Signature :

Investigator Signature:

Name, First name:

Date : Signature :

For peer review only

For peer review only - http://bmjopen.bmj.com/site/about/guidelines.xhtml

Reporting checklist for protocol of a clinical trial.

Based on the SPIRIT guidelines.

Instructions to authors

Complete this checklist by entering the page numbers from your manuscript where readers will find each of the items listed below.

Your article may not currently address all the items on the checklist. Please modify your text to include the missing information. If you are certain that an item does not apply, please write "n/a" and provide a short explanation.

Upload your completed checklist as an extra file when you submit to a journal.

In your methods section, say that you used the SPIRITreporting guidelines, and cite them as:

Chan A-W, Tetzlaff JM, Altman DG, Laupacis A, Gøtzsche PC, Krleža-Jerić K, Hróbjartsson A, Mann H, Dickersin K, Berlin J, Doré C, Parulekar W, Summerskill W, Groves T, Schulz K, Sox H, Rockhold FW, Rennie D, Moher D. SPIRIT 2013 Statement: Defining standard protocol items for clinical trials. Ann Intern Med. 2013;158(3):200-207

		Reporting Item	Page Number
**Administrative information**			
Title	#1	Descriptive title identifying the study design, population, interventions, and, if applicable, trial acronym	1
Trial registration	#2a	Trial identifier and registry name. If not yet registered, name of intended registry	4
Trial registration: data set	#2b	All items from the World Health Organization Trial Registration Data Set	Throughout manuscript
Protocol version	#3	Date and version identifier	21
Funding	#4	Sources and types of financial, material, and other support	30
Roles and responsibilities:	#5a	Names, affiliations, and roles of protocol contributors	1,2,30

For peer review only

contributorship			
Roles and responsibilities: sponsor contact information	#5b	Name and contact information for the trial sponsor	29
Roles and responsibilities: sponsor and funder	#5c	Role of study sponsor and funders, if any, in study design; collection, management, analysis, and interpretation of data; writing of the report; and the decision to submit the report for publication, including whether they will have ultimate authority over any of these activities	30
Roles and responsibilities: committees	#5d	Composition, roles, and responsibilities of the coordinating centre, steering committee, endpoint adjudication committee, data management team, and other individuals or groups overseeing the trial, if applicable (see Item 21a for data monitoring committee)	23,30
**Introduction**			
Background and rationale	#6a	Description of research question and justification for undertaking the trial, including summary of relevant studies (published and unpublished) examining benefits and harms for each intervention	6-8
Background and rationale: choice of comparators	#6b	Explanation for choice of comparators	6-7
Objectives	#7	Specific objectives or hypotheses	8-10
Trial design	#8	Description of trial design including type of trial (eg, parallel group, crossover, factorial, single group), allocation ratio, and framework (eg, superiority, equivalence, non-inferiority, exploratory)	
**Methods: Participants, interventions, and outcomes**			

For peer review only

Study setting	#9	Description of study settings (eg, community clinic, academic hospital) and list of countries where data will be collected. Reference to where list of study sites can be obtained	Supplemental table 1
Eligibility criteria	#10	Inclusion and exclusion criteria for participants. If applicable, eligibility criteria for study centres and individuals who will perform the interventions (eg, surgeons, psychotherapists)	10-12
Interventions: description	#11a	Interventions for each group with sufficient detail to allow replication, including how and when they will be administered	15-16
Interventions: modifications	#11b	Criteria for discontinuing or modifying allocated interventions for a given trial participant (eg, drug dose change in response to harms, participant request, or improving / worsening disease)	18, 19,22
Interventions: adherance	#11c	Strategies to improve adherence to intervention protocols, and any procedures for monitoring adherence (eg, drug tablet return; laboratory tests)	19-21
Interventions: concomitant care	#11d	Relevant concomitant care and interventions that are permitted or prohibited during the trial	13,23
Outcomes	#12	Primary, secondary, and other outcomes, including the specific measurement variable (eg, systolic blood pressure), analysis metric (eg, change from baseline, final value, time to event), method of aggregation (eg, median, proportion), and time point for each outcome. Explanation of the clinical relevance of chosen efficacy and harm outcomes is strongly recommended	9-10
Participant timeline	#13	Time schedule of enrolment, interventions (including any run-ins and washouts), assessments, and visits for participants. A schematic diagram is highly recommended (see Figure)	See figure 1<br>8,12,15,16-19
Sample size	#14	Estimated number of participants needed to achieve study objectives and how it was determined, including clinical and statistical assumptions	27

For peer review only

		supporting any sample size calculations	
Recruitment	#15	Strategies for achieving adequate participant enrolment to reach target sample size	Not listed
**Methods: Assignment of interventions (for controlled trials)**			
Allocation: sequence generation	#16a	Method of generating the allocation sequence (eg, computer-generated random numbers), and list of any factors for stratification. To reduce predictability of a random sequence, details of any planned restriction (eg, blocking) should be provided in a separate document that is unavailable to those who enrol participants or assign interventions	15,16,22
Allocation concealment mechanism	#16b	Mechanism of implementing the allocation sequence (eg, central telephone; sequentially numbered, opaque, sealed envelopes), describing any steps to conceal the sequence until interventions are assigned	22
Allocation: implementation	#16c	Who will generate the allocation sequence, who will enrol participants, and who will assign participants to interventions	15
Blinding (masking)	#17a	Who will be blinded after assignment to interventions (eg, trial participants, care providers, outcome assessors, data analysts), and how	22
Blinding (masking): emergency unblinding	#17b	If blinded, circumstances under which unblinding is permissible, and procedure for revealing a participant's allocated intervention during the trial	N/A
**Methods: Data collection, management, and analysis**			
Data collection plan	#18a	Plans for assessment and collection of outcome, baseline, and other trial data, including any related processes to promote data quality (eg, duplicate	24-25

For peer review only

		measurements, training of assessors) and a description of study instruments (eg, questionnaires, laboratory tests) along with their reliability and validity, if known. Reference to where data collection forms can be found, if not in the protocol	
Data collection plan: retention	#18b	Plans to promote participant retention and complete follow-up, including list of any outcome data to be collected for participants who discontinue or deviate from intervention protocols	Not listed
Data management	#19	Plans for data entry, coding, security, and storage, including any related processes to promote data quality (eg, double data entry; range checks for data values). Reference to where details of data management procedures can be found, if not in the protocol	25
Statistics: outcomes	#20a	Statistical methods for analysing primary and secondary outcomes. Reference to where other details of the statistical analysis plan can be found, if not in the protocol	25-26
Statistics: additional analyses	#20b	Methods for any additional analyses (eg, subgroup and adjusted analyses)	26-27
Statistics: analysis population and missing data	#20c	Definition of analysis population relating to protocol non-adherence (eg, as randomised analysis), and any statistical methods to handle missing data (eg, multiple imputation)	26
**Methods: Monitoring**			
Data monitoring: formal committee	#21a	Composition of data monitoring committee (DMC); summary of its role and reporting structure; statement of whether it is independent from the sponsor and competing interests; and reference to where further details about its charter can be found, if not in the protocol. Alternatively, an explanation of why a DMC is not needed	23
Data monitoring:	#21b	Description of any interim analyses and stopping	23

For peer review only

interim analysis		guidelines, including who will have access to these interim results and make the final decision to terminate the trial	
Harms	#22	Plans for collecting, assessing, reporting, and managing solicited and spontaneously reported adverse events and other unintended effects of trial interventions or trial conduct	23
Auditing	#23	Frequency and procedures for auditing trial conduct, if any, and whether the process will be independent from investigators and the sponsor	24
**Ethics and dissemination**			
Research ethics approval	#24	Plans for seeking research ethics committee / institutional review board (REC / IRB) approval	4
Protocol amendments	#25	Plans for communicating important protocol modifications (eg, changes to eligibility criteria, outcomes, analyses) to relevant parties (eg, investigators, REC / IRBs, trial participants, trial registries, journals, regulators)	22
Consent or assent	#26a	Who will obtain informed consent or assent from potential trial participants or authorised surrogates, and how (see Item 32)	29-30
Consent or assent: ancillary studies	#26b	Additional consent provisions for collection and use of participant data and biological specimens in ancillary studies, if applicable	No additional consent see page 30
Confidentiality	#27	How personal information about potential and enrolled participants will be collected, shared, and maintained in order to protect confidentiality before, during, and after the trial	24
Declaration of interests	#28	Financial and other competing interests for principal investigators for the overall trial and each study site	30
Data access	#29	Statement of who will have access to the final trial dataset, and disclosure of contractual agreements that limit such access for investigators	Not provided

For peer review only

Ancillary and post trial care	#30	Provisions, if any, for ancillary and post-trial care, and for compensation to those who suffer harm from trial participation	N/A
Dissemination policy: trial results	#31a	Plans for investigators and sponsor to communicate trial results to participants, healthcare professionals, the public, and other relevant groups (eg, via publication, reporting in results databases, or other data sharing arrangements), including any publication restrictions	29
Dissemination policy: authorship	#31b	Authorship eligibility guidelines and any intended use of professional writers	29
Dissemination policy: reproducible research	#31c	Plans, if any, for granting public access to the full protocol, participant-level dataset, and statistical code	Not planned
**Appendices**			
Informed consent materials	#32	Model consent form and other related documentation given to participants and authorised surrogates	Appendix 1
Biological specimens	#33	Plans for collection, laboratory evaluation, and storage of biological specimens for genetic or molecular analysis in the current trial and for future use in ancillary studies, if applicable	Not provided

None The SPIRIT checklist is distributed under the terms of the Creative Commons Attribution License CC-BY-ND 3.0. This checklist can be completed online using https://www.goodreports.org/, a tool made by the EQUATOR Network in collaboration with Penelope.ai

For peer review only
